# Donor-acceptor engineering of a triplet-exciton-optimized MOF photocatalyst for efficient singlet oxygen-mediated oxidation

**DOI:** 10.1093/nsr/nwaf024

**Published:** 2025-01-22

**Authors:** Kai Wang, Chang-Tai Li, Guo-Lei Zhang, Han-Yu Wang, Lin Geng, Bo Zhang, Mei-Hui Yu, Jijie Zhang, Ze Chang, Xian-He Bu

**Affiliations:** School of Materials Science and Engineering, TKL of Metal and Molecule-Based Material Chemistry, Nankai University, Tianjin 300350, China; School of Materials Science and Engineering, TKL of Metal and Molecule-Based Material Chemistry, Nankai University, Tianjin 300350, China; School of Materials Science and Engineering, TKL of Metal and Molecule-Based Material Chemistry, Nankai University, Tianjin 300350, China; School of Materials Science and Engineering, TKL of Metal and Molecule-Based Material Chemistry, Nankai University, Tianjin 300350, China; School of Materials Science and Engineering, TKL of Metal and Molecule-Based Material Chemistry, Nankai University, Tianjin 300350, China; School of Materials Science and Engineering, TKL of Metal and Molecule-Based Material Chemistry, Nankai University, Tianjin 300350, China; School of Materials Science and Engineering, TKL of Metal and Molecule-Based Material Chemistry, Nankai University, Tianjin 300350, China; School of Materials Science and Engineering, TKL of Metal and Molecule-Based Material Chemistry, Nankai University, Tianjin 300350, China; School of Materials Science and Engineering, TKL of Metal and Molecule-Based Material Chemistry, Nankai University, Tianjin 300350, China; School of Materials Science and Engineering, TKL of Metal and Molecule-Based Material Chemistry, Nankai University, Tianjin 300350, China; State Key Laboratory of Elemento-Organic Chemistry, College of Chemistry, Nankai University, Tianjin 300071, China

**Keywords:** donor-acceptor MOF, photocatalyst, triplet exciton, singlet oxygen, energy transfer

## Abstract

The exploration of photocatalysts (PCs) for efficient singlet oxygen (^1^O_2_)-based photocatalytic oxidation is critical and challenging. Herein, a new series of donor-acceptor metal-organic frameworks (D-A MOFs) are constructed through the engineering of the D-A system, and investigated as PCs for the ^1^O_2_ oxidation reaction. By regulating the intersystem crossing and reversed intersystem crossing features of the D-A system, D-A MOFs could reveal highly tunable triplet-exciton generation. Via the synergy of the enhanced electron transfer properties and the effective energy transfer to ground-state O_2_, the optimized D-A MOF (**C1**) could reveal remarkable activity toward ^1^O_2_ generation under appropriate irradiation, which is fully proven by the highly efficient oxidation and detoxification of mustard simulant 2-chloroethyl ethyl sulfide into 2-chloroethyl ethyl sulfoxide (conversion and selectivity >99% within 15 min). Moreover, the application of **C1** for the photocatalytic oxidation of dihydroartemisinic acid to artemisinin results in the highest selectivity and yield (selectivity 88% and conversion >99% at 25°C) among all reported homo- or heterogeneous PCs.

## INTRODUCTION

Organic conversion driven by the photocatalytic approach is an effective way to convert solar energy into chemical energy, providing an environmentally friendly and sustainable method for producing high-value organic compounds [1–5]. With plenty of advantages, including mild reaction conditions and better universality, photocatalysis has become one of the core technologies in the field of efficient synthetic chemistry and will be important to the sustainable development of human society [6–9]. Molecular oxygen (O_2_) is attracting attention in organic synthesis as an abundant and economical oxidizing agent [10–15]. In recent years, photoredox catalysis has moved to the forefront of organic chemistry as a powerful strategy for the activation of O_2_. As an efficient and low-cost oxidant, singlet oxygen (^1^O_2_) plays a crucial role in many reaction processes owing to its mild oxidation property [16–19]. Therefore, the efficient generation of ^1^O_2_ utilizing a proper photocatalyst (PC) has emerged as a hot topic.

In consideration of the low visible-light absorption and triplet ground state of O_2_, its activation toward ^1^O_2_ is commonly achieved through the energy transfer (EnT) from the triplet excitons of PCs [20–25]. Therefore, the generation of triplet excitons requires the intersystem crossing (ISC) process from the lowest singlet excited state (S_1_) to the lowest triplet excited state (T_1_), then the EnT from triplet excitons to ground-state oxygen (O_2_) will be preferred in order to produce ^1^O_2_ for photocatalysis. PCs with efficient ^1^O_2_ production generally require intense light absorption, good photostability and effective ISC. Most PCs have large n-π* transitions and small spin-orbit coupling (SOC), resulting in large energy barrier band gaps (∆*E*_ST_) for the conversion of singlet excitons to triplet state excitons on the PC [26–32]. On the other hand, the PCs based on phthalocyanines, porphyrins and precious metal complexes feature a large magnitude of triplet excitons, while the unmatched redox potential of the active moieties will induce the formation of reactive oxygen species (ROS) and corresponding by-products [33–38]. In this regard, the excited-state engineering of PCs toward optimized triplet-exciton generation and utilization could be critical and challenging for the optimization of ^1^O_2_ generation under photocatalytic conditions [39–41].

With regard to triplet-exciton utilization, compounds featuring thermally activated delayed fluorescence (TADF) have been wildly investigated as next-generation emitters with optimized quantum efficiency. The TADF materials are commonly composed of electron donor (D) and electron acceptor (A) moieties that interact through chemical bonding or with a through-space manner. The resultant donor-acceptor (D-A) system can reveal minimal ∆*E*_ST_ (<0.2 eV) based on the proper choice and manipulation of D and A moieties, then the charge transfer (CT)-based emissions of the D-A systems would reveal delayed fluorescence (FL) originating from the reverse intersystem crossover (RISC) of T_1_ to S_1_ [42,43]. In this way, the triplet excitons could be utilized for the optimization of emission performance. A more detailed analysis of the characteristics of TADF materials indicates that the redox potential of the TADF materials could be readily tuned through the rational tuning of the D and A components, and the relatively long lifetime of the delayed FL emission (commonly at the microsecond scale) indicates a long excited lifetime [44–46]. Furthermore, the inherent difference in electron affinity between D and A will lead to a large dipole distance in the backbone. The dipole distance will trigger the electric charge transfer from D to A under the illumination, resulting in an increase of photogenerated electron density in the receptor, so as to realize rapid separation and migration of the photogenerated charge carriers. All these factors could facilitate their performance as PCs [47,48].

For the investigation of advanced PCs, metal-organic frameworks (MOFs) have emerged as a class of promising materials. The highly tunable components and structural features of MOFs allow the rational design and integration of photosensitizing moieties and catalytic active sites for optimized performance, and the highly crystalline property of MOFs can benefit heterogeneous photocatalysis reactions. Accordingly, MOF-based PCs have been widely investigated for various kinds of reactions. Recently, a ligand with TADF features has been utilized for the construction of MOFs for photocatalysis, and the results indicate the potential of the MOFs for CO_2_ photoreduction based on the long excited-state lifetime [49]. However, the targeted construction and tuning of MOFs for triplet-exciton utilization has not been reported. Our group has achieved the rational tuning of TADF in MOFs through a host-guest manner. By utilizing the **guest@NKU-111** MOF (guest@[Cd_3_(tpt)_2_(TPA)_3_(H_2_O)_3_] · 2H_2_O}_n_, tpt = 2,4,6-tri-4-pyridinyl-1,3,5-triazine and TPA = phthalic acid) as a platform for the spatial alignment manipulation of D and A components, a series of MOFs featuring highly tunable TADF have been constructed based on systematic regulation of ∆*E*_ST_ and the ISC/RISC process of the D-A system [50,51]. This achievement indicates the potential of D-A MOFs for efficient triplet-exciton generation and for utilization as PCs for ^1^O_2_-based photocatalytic oxidation reactions.

**Figure 1.**
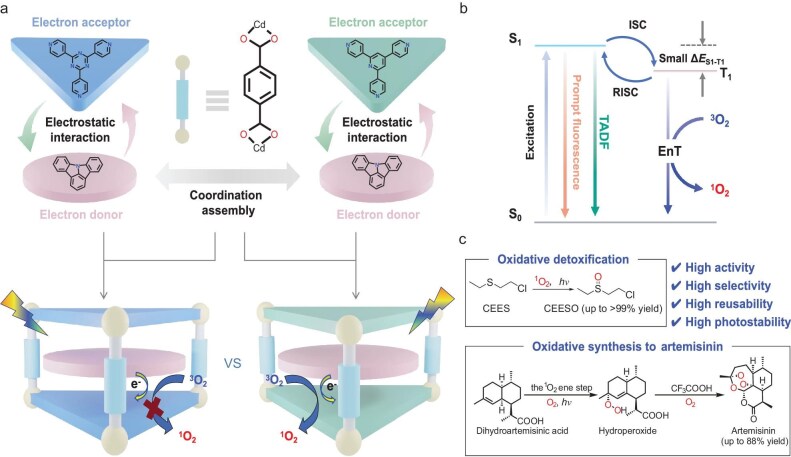
(a) Engineering of the D-A system in MOF PCs for photocatalytic singlet oxygen-mediated oxidations. (b) Schematic mechanism of the generation of ^1^O_2_ based on PCs featuring modulable triplet-exciton generation and the EnT process. (c) Schematic illustration of our D-A MOF PCs for ^1^O_2_-mediated oxidations under visible-light irradiation.

Herein, we report the engineering of D-A systems in MOFs aiming at triplet-exciton optimization for PC application. By utilizing the highly tunable components of the **NKU-111**-type D-A MOFs, an optimized combination of D and A components, with the 2,4,6-tris(4-pyridyl)pyridine (tpp) as the acceptor and indolo[3,2,1-jk]carbazole (Icz) as the donor, has been investigated. The resultant Icz@[Cd_3_(tpp)_2_(TPA)_3_(H_2_O)_3_] · 2H_2_O}_n_ (**C1**), isoreticular to the well-investigated **guest@NKU-111** MOFs, was found to reveal delayed FL and highly improved photogenerated electron transfer (ET) performance compared with the **Icz@NKU-111** (Fig. 1a and b). More detailed systematic investigation indicates that the heavy atom functionalization of the donor (**D1** with 2-bromoIndolo[3,2,1-jk]carbazole, 2-BrIcz), the TPA linker (**E1**, with 2-bromoterephthalic acid, 2-BrTPA) and their synergy (**F1**, with both 2-BrIcz and 2-BrTPA) could effectively regulate the redox potential and electron transport performance of the materials, which is evidenced by their distinctive photophysical properties and photocatalytic performance. Accordingly, **C1** was found to be a highly efficient PC to generate ^1^O_2_ under visible-light irradiation, enabling green oxidation catalysis of a series of substrates, realizing sulfoxidation with high efficiency and selectivity (Fig. 1c). Moreover, the highly active **C1** could be applied to the photocatalytic oxidation of dihydroartemisinic acid (DHAA) to artemisinin to give remarkable activities and selectivity (up to 88% for the ^1^O_2_ ene step). In addition to this being the first report regarding the application of D-A MOFs in photocatalysis, and in addition to the achievement of highly efficient photocatalytic oxidation with the PCs, this work also proves the potential of D-A MOFs as PCs through D-A engineering. This systematic investigation also illustrates principles for the targeted construction and optimization of photocatalytically active D-A MOFs.

## RESULTS AND DISCUSSION

### Engineering of the D-A system in MOFs for optimized photoelectric properties

**Figure 2.**
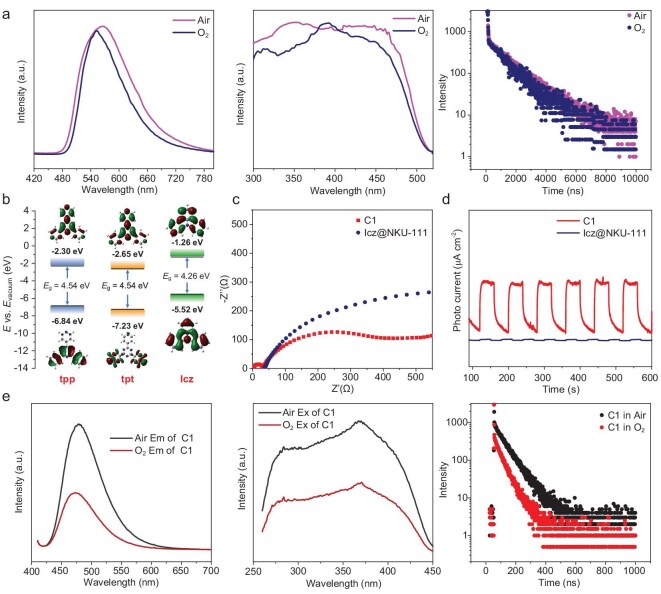
(a) Photophysical properties of **Icz@NKU-111** in air and O_2_, respectively. Emission spectra (left); excitation spectra (middle); PL decay profiles (right). (b) HOMO/LUMO distributions of tpp, tpt and Icz. (c) EIS Nyquist plots of **C1** and **Icz@NKU-111**. (d) Photocurrent tests of **C1** and **Icz@NKU-111**. (e) Photophysical properties of **C1** in air and O_2_, respectively. Emission spectra (left); excitation spectra (middle); PL decay profiles (right).

On the basis of our previous construction of D-A MOFs featuring TADF emissions, the D-A systems in MOFs were engineered with the aim of achieving ^1^O_2_ generation. First, Icz was utilized as the donor to replace the commonly used *N*-phenylcarbazole (Phcz) donor to achieve TADF, since the more rigid skeleton of Icz can reduce non-radiative relaxation. We prepared **Icz@NKU-111** as a prototype by introducing Icz as the donor guest into the acceptor host **NKU-111** (Fig. S1). Bearing in mind the highest occupied molecular orbital (HOMO) of the donor and lowest unoccupied molecular orbital (LUMO) energy level of tpt and Icz (Fig. 2b), **Icz@NKU-111** should be a typical D-A MOF, and its photoluminescence (PL) emission of around 565 nm (Table 1) should be assigned to CT-based emission. It shows a prompt FL with a lifetime of 33.82 ns (τ_p_) and a longer delayed FL with a lifetime up to 1.41 μs (τ_d_) (Table 1), which is typical TADF, as expected [51]. Then the potential of **Icz@NKU-111** for the photosensitizing of O_2_ toward ROS is preliminarily investigated based on the quenching of emission under O_2_ atmosphere. Commonly, O_2_^•−^ can be generated through the ET process, while ^1^O_2_ is produced from the EnT process of triplet excitons. As shown in Fig. 2a, the emission intensity of **Icz@NKU-111** is well retained under O_2_ atmosphere, accompanied by a similar FL decay trend under both air and O_2_ (Fig. 2a). In spite of the long-lived triplet excitons in **Icz@NKU-111** based on the delayed FL mechanism, the well-retained emission in O_2_ atmosphere indicates its insufficient photogenerated ET capacity to conduct effective EnT or ET processes with O_2_, which is not suitable for PC applications.

**Table 1.** Photophysical properties of **Icz@NKU-111, C1, D1, E1** and **F1**^a^.

		**Fluorescence data**					
**Compound** ^a^	**UV-Vis spectra *λ*_max_ (nm)**	***λ*_em_ (nm)**	***φ* (%)**	***τ*_p_ (ns)**	***τ*_d_ (μs)**	***R*_d_ (%)**	**Δ*E*_ST_ (eV)**
**Icz@NKU-111**	350	565	73.18	33.82	1.41	96.03	—
**C1**	395	482	41.28	17	0.116	98.31	0.049
**D1**	370	480	3.01	5.51	2.18	67.76	0.027
**E1**	367	480	30.65	9.66	0.079	94.85	0.027
**F1**	389	470	2.16	1.63	—	—	—

a All the relative measurements were conducted in solid state.

For the improvement of the photogenerated ET capacity of the D-A MOF, the acceptor moiety should also be optimized. In this regard, the tpp molecule was introduced to replace the tpt acceptor. The HOMO/LUMO energy levels of tpp and Icz indicate that a D-A system could also be constructed with tpp as the acceptor (Fig. 2b). Accordingly, Icz@[Cd_3_(tpp)_2_(TPA)_3_(H_2_O)_3_] · 2H_2_O}_n_ (**C1**) was synthesized. The coordination of tpp and TPA linkers with Cd^2+^ ions results in the host framework (Fig. S2). Then, Icz as the guest donor component was incorporated into the cage-based host framework, which is promoted by the confined space and D-A interactions (detailed structural discussion provided in the following section). The steady-state PL spectrum of **C1** at 298 K shows the maximum emission around 482 nm, and the blue shifted emission compared with **Icz@NKU-111** is consistent with the HOMO/LUMO results. The lifetime test of **C1** indicates the occurrence of delayed FL as expected (this will be discussed in detail in the following section). More importantly, different from that of **Icz@NKU-111**, the emission of **C1** could be significantly quenched under O_2_ atmosphere, accompanied by more rapid FL decay (Fig. 2e). These results suggest the occurrence of an EnT or ET process from **C1** to O_2_ to generate ROS. Considering the non-porous nature of the crystal structure of **C1**, the process could only occur at the surface of the crystal. Then the highly O_2_ sensitive emission quenching should be attributed to the synergy of the enhanced photogenerated ET of the MOF and the effective EnT or ET process from the D-A MOF to O_2_. The optimized ET performance of **C1** was also proven by preliminary transient photocurrent measurements and electrochemical impedance spectroscopy (EIS). As shown in Fig. 2c and d, **C1** reveals lower resistance for CT and much higher photocurrent than **Icz@NKU-111** under visible-light irradiation, which indicates that the photogenerated carrier transport capacity of **C1** is far stronger than that of **Icz@NKU-111**. The tpt ligand, with the electron-deficient triazine moiety as a backbone, has a stronger electron-withdrawing ability and weaker photogenerated ET capacity in the framework. In contrast, the weaker electron-withdrawing ability of tpp can promote the transfer of photogenerated electrons compared to that of tpt with the triazine composition. Accordingly, the photoelectric behavior of **C1** (tpp as the acceptor) is superior to that of **Icz@NKU-111** (tpt as the acceptor), which also promotes the quenching of emission by O_2_ exposure due to the more effective ET. All these results indicate that the **C1** based on the Icz-tpp D-A system has potential for effective ROS generation.

### Systematic construction and characterization of D-A MOFs

Based on the preliminary investigation of **C1**, more systematic investigations of the new series of D-A MOFs were performed. Through the coordination-directed assembly of guest, metallic ion and linker species under solvothermal conditions (see Supplementary Data for details), products **C1**, 2-BrIcz@[Cd_3_(tpp)_2_(TPA)_3_(H_2_O)_3_] · 2H_2_O}*_n_* (**D1**), Icz@[Cd_3_(tpp)_2_(2-BrTPA)_3_(H_2_O)_3_] · 2H_2_O}*_n_* (**E1**) and 2-BrIcz@[Cd_3_(tpp)_2_(2-BrTPA)_3_(H_2_O)_3_] · 2H_2_O}*_n_* (**F1**) were obtained, respectively (Fig. S3). Powder X-ray diffraction (PXRD) revealed that **C1, D1, E1** and **F1** are highly crystalline (Fig. S4a and b). Through-space charge transfer (TSCT) interactions can be accessed using these D-A moieties. The successful insertion of the donor guests could be well characterized through the single crystal X-ray diffraction (SCXRD) and ^1^H liquid nuclear magnetic resonance (NMR) spectroscopy tests of the digested crystal samples (Table S1, Figs S5–S8). The relatively high guest loading (98.28% to 99.75%) indicates the strong interaction between the donor and acceptor molecules during the assembly process, which could also benefit the catalytic performance of the materials.

Detailed structural analysis shows that the unit cell parameters of the compounds match the classical **NKU-111** well [52], deriving isostructural 3,4-connected tfz topology when considering the tpp ligands and Cd^2+^ metal centers as three- and four-coordinated nodes, respectively (Figs S10 and S11). The framework consists of a 3-fold interpenetrated network, including a hexagonal prism cage formed by interlocking two triangular prism cages from different networks (Figs S12a and b, and S13). Icz or 2-BrIcz resides in cages of the host framework in a disordered manner while only one molecule can be allowed in each cage due to the restricted inner space of the cage (Fig. S12c). The Icz guest and the tpp ligand exhibit the face-to-face stacking mode induced by the confined interspace of the cage. For example, the distance between the centers of the donors and acceptors of **C1** is in the range of 3.47–3.54 Å (Fig. S14). The columnar packing of the cages perpendicular to the plane of the guests could result in face-to-face DAADAAD packing of the donor and acceptor, which could result in strong intermolecular π–π interactions [53–55]. It is worth noting that the stacking of the D and A components in a highly ordered manner may create a path for the transfer of photogenerated electrons, which is highly desired for PCs. Infrared (IR) spectra of **C1** to **F1** (Fig. S15) reveal similar adsorption peaks. From the comparison of IR spectra of **C1** to **F1**, the obvious peaks around 700 cm^−1^ could be attributed to the vibration of the Icz backbone, indicating the successful incorporation of the guest into the framework. The thermal stability of **C1**–**F1** was demonstrated by thermogravimetric analysis (TGA), where negligible structure decomposition was observed up to 200°C (Fig. S16).

### Excited-state features of the D-A MOFs

On the basis of the successful construction of the D-A MOFs, their excited-state features were characterized through their photophysical properties. PL spectra of **C1**–**F1** (Figs S18b, S20b, S22b and S24b) display noticeable emission peaks around 470–480 nm. The comparison of emission spectra of the guests, ligands and these compounds (**C1**–**F1)** shows that the emission of compounds is different from that of both the guests and the ligands (Figs S26–S29). Therefore, the linker- and donor-dependent emissions could be assigned to the CT-based emission based on the distinctive D-A systems. The photoluminescence quantum yield (PLQY) of the D-A MOFs were also determined (Table 1). **C1** reveals relatively high PLQY of 41.28% compared with that of **D1** (3.01%), **E1** (30.65%) and **F1** (2.16%), while D-A MOFs that reveal higher PLQY are expected to be more efficient in the generation of excitons. For a better understanding of the excited-state features of the D-A MOFs, the PL decay profile of **C1**–**F1** was investigated at room temperature (RT) in the air (Table 1). **C1** possessed a prompt FL of 17 ns (τ_p_) and a delayed FL of 0.116 μs (τ_d_) in time-resolved luminescence probing (Fig. S19). A prompt FL of 5.51 ns (τ_p_) and a delayed FL of 2.18 μs (τ_d_) were observed for **D1** (Fig. S21). **E1** had a prompt FL of 9.66 ns (τ_p_) and a delayed FL of 0.079 μs (τ_d_) (Fig. S23). **F1** only has a short lifetime of 1.63 ns (τ_p_) (Fig. S25). The observation of delayed FL for **C1, D1** and **E1** indicates the participation of a triplet-excited state during the emission process, as expected. The extended delayed lifetime of **D1** compared with that of **C1** should be attributed to the internal heavy atom effect (HAE) of the –Br group from 2-BrIcz, which could effectively enhance the SOC and the corresponding ISC and RISC processes. For **E1** and **F1**, the 2-BrTPA was introduced, aiming at the further enhancement of external HAE to benefit the delayed FL, while the results indicate that the presence of 2-BrTPA just decreases (for **E1**) or inhibits (for **F1**) the occurrence of delayed FL. These results indicate that the internal HAE should be more effective toward the tuning of the delayed FL and the corresponding triplet-exciton generation of the D-A systems.

**Figure 3.**
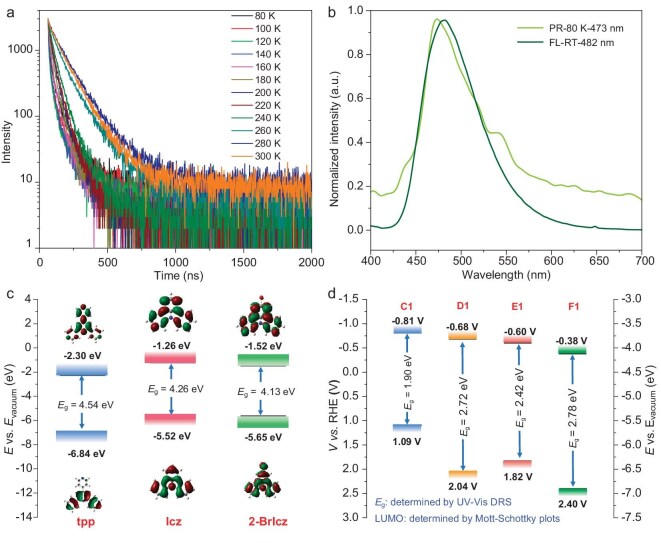
(a) Temperature-dependent PL decay profiles of **C1**. (b) Phosphorescence (PR) spectrum at 77 K and fluorescence (FL) spectrum at room temperature (RT) of **C1**. (c) HOMO/LUMO distributions of planar tpp and donors. (d) Schematic diagrams of the optical band gaps of the four D-A MOFs.

For a more detailed understanding of the ISC and RISC features of the D-A MOFs featuring delayed FL, their emission spectra and decay profiles were further determined under different temperatures. It should be noted that the profiles show much longer lifetimes than the common nanosecond-scale FL emission, which indicates the participation of triplet excitons. For **C1**, the decay profiles (Fig. 3a) show a decreasing trend when the temperature increases from 80 to 120 K, which could be attributed to the enhanced deactivation of triplet excitons through the non-radiative relaxation process at higher temperatures. An increasing trend was observed from 120 to 280 K, which might be attributed to the enhanced delayed FL emission that originated from the high-temperature-promoted RISC process of triplet excitons. The decreasing trend from 280 to 300 K should also be attributed to the enhanced non-radiative relaxation process. For the emission spectra (Fig. S30), the change of emission intensity should be assigned to the equilibrium between the emission process and the non-radiative relaxation process, both of which are temperature-sensitive. The decreased emission intensity from 80 to 160 K could be attributed to the enhanced non-radiative relaxation process in response to the increased temperature. As the temperature further increased (from 160 to 240 K), the enhanced RISC process might have overcome the non-radiative relaxation process to enhance the emission intensity. At a higher temperature (from 240 to 300 K), the non-radiative relaxation process could be dominant again compared with the RISC process, therefore the emission intensity shows a decreasing trend [51,56–58]. The prompt emission spectra recorded at room temperature and the delayed spectra (delayed for 1 ms) recorded at 80 K (Fig. 3b) show very similar profiles, indicating the nearly identical excited energy levels of S_1_ and T_1_. The extended emission lifetime at a higher temperature suggests that the triplet excitons are preferred due to the enhanced ISC process, which is highly desired for the effective generation and utilization of triplet excitons under ambient conditions.

In contrast to **C1, D1** reveals typical TADF behavior (Figs S31–S33), as indicated by the decreased total lifetime but increased proportion of delayed lifetime. The *ΔE_ST_* was determined to be 0.027 eV based on the FL recorded at room temperature and the PR recorded at 80 K. The emission intensity decreased along with the increased temperature from 90 to 160 K, while an increasing trend was observed from 160 to 280 K. These results indicate that the functionalization of the –Br group to the donor species (2-BrIcz) could affect the excited state by regulating the HOMO of the donor. On the other hand, the increased molecular weight could suppress the vibration to enhance the emission intensity at an even higher temperature.

The photophysical properties of **E1** were found to be similar to those of **C1**. The emission spectra (Fig. S34) of **E1** show increased intensity from 80 to 150 K, and the intensity decreased along with the increase in temperature from 150 to 300 K. For the decay profiles (Fig. S35), a decreasing trend was observed in the 80 to 120 K range, while an extended trend was observed from 120 to 280 K. All these results show that the triplet excitons are also preferred for **E1**, indicating a further enhanced ISC than RISC process at a higher temperature. The similar performances of **E1** and **C1** indicate that the D-A system composed of Icz and tpp could result in a minimized energy gap between the S_1_ and T_1_. Generally, the distinctive photophysical properties of the four MOFs indicate the potential and versatility of D-A MOFs as a platform for excited-state engineering.

### Photoelectronic and electrochemical properties of the D-A MOFs

As shown in Fig. 3c, the tendency of the gap between the HOMO of the donor and LUMO of the acceptor could roughly predict the emission of the D-A MOFs and matched well with their experimental emission maximums, indicating the D-A CT origin of the emissions. To find out the photoelectronic properties of the D-A MOFs, Ultraviolet–visible (UV–Vis) absorption spectroscopy measurements and Mott-Schottky tests were performed. Based on the Tauc plots (Fig. S38), the band gap values (*E*_g_) were calculated to be 1.90, 2.72, 2.42 and 2.78 eV for **C1, D1, E1** and **F1**, respectively. It is noteworthy that the optical band gap of **C1** is narrower than many of the reported MOFs [59–61]. The LUMO levels were determined from the flat-band potential (*E*_fb_) obtained from Mott-Schottky plots. As shown in Fig. S39, the positive slope of the fitted line indicates that **C1, D1, E1** and **F1** are *n*-type semiconductors, and their *E*_fb_ values were estimated to be −1.01, −0.88, −0.80 and −0.58 V vs. Ag/AgCl (−0.81, −0.68, −0.60 and −0.38 V vs. reversible hydrogen electrode (RHE)), respectively. The corresponding HOMO positions therefore were calculated to be 1.09 (**C1**), 2.04 (**D1**), 1.82 (**E1**) and 2.40 (**F1**) V vs. RHE by using the band gap energy equation (*E*_HOMO_ = *E*_LUMO_ − *E*_g_), and the corresponding data are summarized in Fig. 3d. **C1** has the lowest HOMO and LUMO levels, compared with the other three MOFs. Introduction of Br atoms in **D1** to **F1** rendered them a selective raised LUMO level. A comparison of LUMO/HOMO energy among **C1** to **F1** indicated that the band gaps of materials are highly tunable based on D-A component manipulation.

To further valuate the electrochemical properties of the D-A MOFs, their charge carrier mobility and separation efficiency were evaluated through transient photocurrent and EIS measurements. As shown in Fig. S40, the EIS tests reveal that **C1** exhibits relatively small radii, indicative of lower resistance for CT. Meanwhile, **C1** reveals a much higher photocurrent than the other three D-A MOFs (**D1** to **F1**) under visible-light irradiation (Fig. S41), suggesting that the introduction of strong electron acceptors into the system is indeed beneficial for charge separation efficiency. The charge separation efficiency can be regulated in the D-A systems by tuning the electron-donating capacity of the donors. Valence band X-ray photoelectron spectroscopy (VB-XPS) measurements were employed to assess the validity of the above band gap energy analysis. As shown in Fig. S43, the VB edges (HOMO positions) of **C1** were consistent with the values obtained from UV-vis diffuse reflectance spectroscopy (DRS) and Mott-Schottky techniques.

### ROS generation

On the basis of the determination of the photophysical and photoelectronic properties of the D-A MOFs, **C1** has been recognized as a potential PC, featuring more efficient triplet-exciton generation and utilization compared with **D1** and **E1**. Therefore, the performance of the D-A MOFs with regard to the generation of ROS under photoexcitation conditions was investigated. Direct evidence for the generation of ROS may be obtained from electron paramagnetic resonance (EPR) measurements. The generation of ^1^O_2_ could be confirmed by the EPR spectra in the presence of ^1^O_2_ scavenger. Therefore, 2,2,6,6-tetramethylpiperidine (TEMP), a well-known ^1^O_2_ probe, is selected to trap ^1^O_2_ to generate stable nitroxide radical 2,2,6,6-tetramethylpiperidine-N-oxyl (TEMPO) for determination. For **C1, D1** and **E1**, the characteristic 1 : 1 : 1 triplet signal of TEMPO could be observed in the EPR spectra, indicating the generation of ^1^O_2_ under light irradiation (Fig. S44a). In contrast, the signal with the presence of **F1** is very weak. Further control experiment results also confirm that ^1^O_2_ was only generated with the presence of the D-A MOFs (Figs S44b and S45a), indicating the potential of **C1** as a PC for ^1^O_2_-based photooxidation reactions. While in the presence of a superoxide radical anion (O_2_^•−^) trapping agent (5,5-dimethyl-1-pyrroline N-oxide, DMPO), no signal was detected, which excluded the involvement of O_2_^•−^ (Fig. S45b). Additionally, to further assess the ability of **C1** to generate ^1^O_2_, 1,3-diphenylisobenzofuran (DPBF) was selected as an indicator to monitor the production of ^1^O_2_. Upon photoirradiation (*λ* = 500 nm) of oxygen-saturated dimethylformamide (DMF) solution (20 mL) of DPBF (1.0 mg) in the presence of **C1** (10 mg), the absorption of DPBF decreased, with <10% of DPBF remaining after photoirradiation for 400 s, according to the intensity of the peak (415 nm) in the UV-vis spectra (Fig. S46). These results are consistent with the photophysical and photoelectronic properties of the D-A MOFs, indicating the highest efficiency of **C1** for ^1^O_2_ generation.

### Photocatalytic CEES detoxification performance of the D-A MOFs

**Figure 4.**
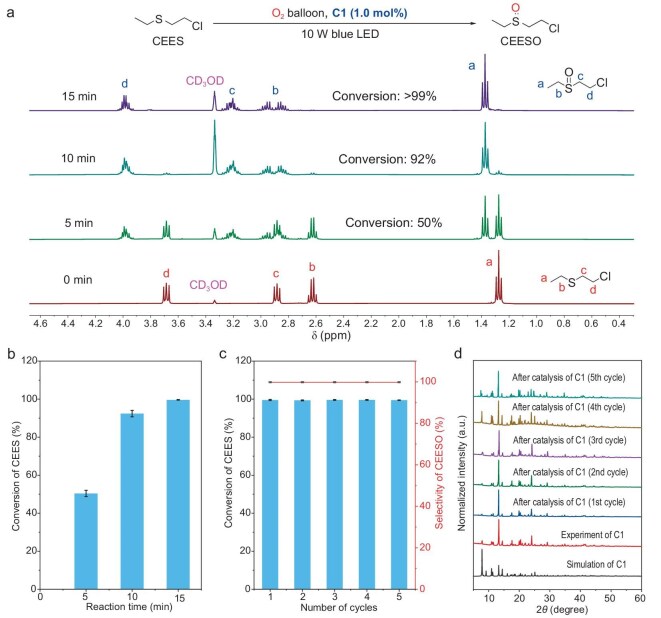
(a) Time-dependent ^1^H NMR analysis of the photooxidation of CEES in the presence of **C1** under a blue LED at 25°C. (b) Conversion of CEES to CEESO in the presence of **C1** under O_2_. (c) Reusability of **C1** over five consecutive cycles of CEES photooxidation. The reaction progress was monitored by ^1^H NMR with five recycling experiments. (d) PXRD patterns of **C1** after cycled reactions.

The above excellent photoelectric performance and ^1^O_2_ generation of **C1** prompted us to study its potential as heterogeneous PCs in ^1^O_2_ oxidation reactions, and the oxidation of 2-chloroethyl ethyl sulfide (CEES) was selected as a model reaction. Sulfur mustard (HD) is a toxic protein foaming agent that can cause skin blistering and respiratory irritation, and can even be life-threatening [62]. Selective oxidation of sulfide to non-toxic sulfoxide is one of the effective methods of HD degradation [63–66]. In this regard, CEES is commonly applied as a HD simulant for investigation. Inspired by the above results, an oxidative degradation reaction of CEES with **C1** was conducted under blue LED irradiation (425 nm, 350 mW/cm^2^). The reaction process was monitored by ^1^H NMR spectra, and the results indicated that **C1** displayed high catalytic activity, needing only 15 min for the conversion of >99% of CEES (1% **C1** loading) into the non-toxic oxidation product 2-chloroethyl ethyl sulfoxide (CEESO) under O_2_ atmosphere (Fig. 4a and b). Control experiments were performed to investigate the influence of the catalyst, atmosphere and light source. No desired product was found in the absence of **C1**, indicating its key role as a PC for the reaction (Table S3, entry 2). Control experiments in the dark or under N_2_ atmosphere result in negligible conversion, suggesting that light and O_2_ are indispensable reaction parameters (Table S3, entries 3, 4, 5). Various solvents were selected and the results indicate that polar solvents (i.e. CD_3_OD) may be beneficial for the photocatalytic aerobic oxidation of CEES. The photocatalytic performance decreased significantly when a green or red LED was used as the light source, indicating that the energy of blue light is critical for electron excitation (Table S3, entries 12 and 13). Based on its higher efficiency for ^1^O_2_ generation, **C1** was found to have the best photocatalytic performance with regard to CEES oxidation among the four D-A MOFs. The yield was decreased to 42%, 55% and 30% when **D1, E1** and **F1** were used instead as PCs under the same reaction conditions (Table S3, entries 14, 15, 16), consistent with their ^1^O_2_ generation efficiency and photoelectric properties. Moreover, upon addition of the singlet oxygen quencher, 1,4-diazabicyclo[2.2.2]octane (DABCO), the reaction was arrested (Table S3, entry 6), suggesting the ^1^O_2_ does indeed play a crucial role in the reaction. Furthermore, a variety of sulfide derivatives (with either electron-withdrawing or electron-donating groups) were also employed as substrates with **C1** as a PC under optimized conditions. With different mustard simulants (Fig. S48), the PC **C1** also showed excellent photocatalytic activity, affording the corresponding oxidation products sulfoxide with high conversion (>99%) and selectivity (>99%) with visible light. The apparent quantum yield (AQY) of **C1** at 425 nm was measured as 12%, better than most reported MOFs. The universality of **C1** toward different mustard simulants should be attributed to the high reactivity of ^1^O_2_ with regard to the homogeneous reaction in the solution, and the non-porous nature of the D-A MOFs preventing the configurational selectivity of the reactions.

Aiming at the application of the PC under practical conditions, we examined the photocatalytic activity of **C1** under solvent-free condition, where it fully transforms CEES into CEESO within 15 min. Impressively, the catalytic activity is comparable to most previously reported outstanding MOF-based PCs (Table S7). To evaluate the reusability of **C1**, we carried out recycling experiments with **C1** as the PC for the thioanisole oxidation reaction (Fig. 4c). Experimental results showed that no significant decrease of photocatalytic efficiency was observed over five cycles. The PXRD patterns of the recovered **C1** revealed no obvious changes compared to the pristine **C1**, suggesting the high stability of **C1** after the reaction (Fig. 4d). We conducted PXRD testing on **C1** from 120 to 280 K. Through PXRD results we found that the sample is stable upon cooling and heating from 120 to 280 K (Fig. S49). *In situ* variable-temperature PXRD measurements were also carried out. As shown in Fig. S50, **C1** has good thermal stability, with a decomposition temperature of ∼200°C, indicating its potential as a PC under harsh conditions.

### Photocatalytic synthesis of artemisinin with the D-A MOFs

Encouraged by the above results, the performance of **C1** for the photocatalytic synthesis of artemisinin was further evaluated. As an important antimalarial drug, the environmental and economic costs of artemisinin synthetic production are relatively high [67]. It is necessary to develop efficient methods for the synthesis of artemisinin in order to reduce operational steps and improve the yield of artemisinin. Recently, the photocatalytic semi-synthesis of artemisinin using DHAA was considered to be a cost-effective approach instead of extraction or total synthesis methods. Mechanistically, the conversion of DHAA to artemisinin generally undergoes complex oxidation/rearrangements including peroxidation via ^1^O_2_-induced Schenck ene reaction, followed by an acid-induced Hock cleavage under ^3^O_2_ [68,69]. There is no doubt that the ^1^O_2_ ene step is the key to determining reaction selectivity in the photooxidation process. It should be noted that most PCs give yields between 50% and 60% after optimization due to the inevitable formation of by-products during the ^1^O_2_ ene step [70–72]. Herein, the reaction was conducted with DHAA in CD_3_OD with 1 atm O_2_ under blue LED irradiation (425 nm, 350 mW/cm^2^) at ambient temperature (25°C). Remarkably, in comparison to the reported most effective homogeneous catalyst Ru(bpy)_3_Cl_2_, **C1** shows almost full conversion and a higher yield of targeted product (Table 2 and Table S8) [73]. The presence of ^1^O_2_ can lead to possible regioisomeric hydroperoxide **1**, which can be transformed into artemisinin [74].

**Table 2.** Photocatalytic oxidation of DHAA to artemisinin by **C1**.

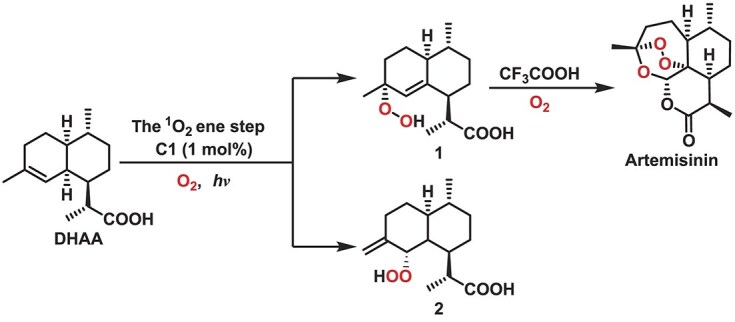							
	**Run 1**		**Run 2**		**Run 3**		
**Catalyst** *^a^*	**Conversion (%)**	**Selectivity (%)**	**Conversion (%)**	**Selectivity (%)**	**Conversion (%)**	**Selectivity (%)**	**Ref.**
Ru(bpy)_3_Cl_2_	>99*^b^*	47*^c^*	N.A.		N.A.		[73]
PCN-808-BDBR	92	42	88	51	90	49	[73]
**C1**	>99	88	>99	88	>99	88	**This work**

a Reactions were run on a 0.1 mmol scale

b Conversion

c Selectivity was determined by ^1^H NMR using biphenyl as the internal standard

The only other isomer generated in the ^1^O_2_ ene step is **2**. According to the result of ^1^H NMR spectra, a **1 **: **2** ratio of 88 : 12 has been determined with the presence of **C1** as a PC, indicating a selectivity up to 88% of the ^1^O_2_ ene step (Fig. S53). The formation of **1** was also detected in the ^1^O_2_ ene step by high-resolution mass spectrometry (HRMS) (Fig. S54). After centrifuging and filtering, the resulting filtrate containing hydroperoxide **1** is then converted to artemisinin in an acid-catalyzed rearrangement followed by a second oxygenation, and a total yield of ∼88% is achieved based on the high conversion (>99%), which is the highest value to the best of our knowledge. The **C1** has been found to exhibit high catalytic selectivity at both lower temperature (91% at 5°C) (entry 8, Table S9) and higher temperature (85% at 45°C) (entry 9, Table S9), which is superior to the reported PCs, for which high selectivity could only be achieved at low temperatures. This could be rationalized by the fact that the generation of triplet excitons and the corresponding ^1^O_2_ could be promoted at higher temperatures for **C1**. Moreover, the reaction with **C1** as the PC can tolerate a wide range of solvents, including CH_3_CN and CH_3_OH, with high selectivity of the ^1^O_2_ ene step. Screening of potential solvents indicates that CH_3_CN was the optimal choice, except for CD_3_OD (Table S10). The leaching experiments indicated its excellent durability (Fig. S55). These results indicate the critical role of **C1** for the achievement of remarkable catalytic performance. PXRD patterns were also measured after catalytic tests on **C1** to check the stability of the PC (Fig. S56). The patterns are consistent with the as-synthesized ones, suggesting the high stability of the material.

## CONCLUSIONS

In summary, with the aim of investigating new PC materials for highly selective ^1^O_2_ oxidation and their application, we have constructed a new series of novel D-A host-guest MOFs featuring a highly tunable excited state for efficient triplet-exciton generation. The introduction of 2,4,6-tris(4-pyridyl)pyridine acceptor and indolo[3,2,1-jk]carbazole derivate donors into the MOF results in D-A systems featuring delayed FL and the generation of triplet excitons can be readily modulated by HAE. Systematic characterization of the D-A MOFs reveals that **C1** has the best performance in triplet-exciton generation and ET, which makes it highly efficient for the generation of ^1^O_2_ through the EnT process with O_2_ molecules. Accordingly, the performance of **C1** as a PC for the ^1^O_2_ oxidation reaction was evaluated with the oxidation of CEES as a model reaction, and high conversion (>99%) and selectivity (>99%) could be achieved under blue LED irradiation (425 nm, 350 mW/cm^2^) in a relatively short time, indicating the remarkable photocatalytic activity of **C1**. Moreover, when **C1** is applied to the photocatalytic oxidation of DHAA to artemisinin, it reveals the highest selectivity and yield of artemisinin among all reported homo- or heterogeneous PCs for artemisinin production. Besides the discovery of the **C1** as a highly efficient PC for ^1^O_2_ oxidation reactions, the results mentioned above also indicate the potential of D-A MOFs as a platform for further advancement in the exploration of novel PCs based on flexible excited-state engineering.

## METHODS

Detailed preparation and characterization methods of materials are available in the Supplementary Data.

## Supplementary Material

nwaf024_Supplemental_File

## Data Availability

CCDC 2306707, 2308341, 2314760 and 2322732 contain the supplementary crystallographic data for this paper. These data can be obtained free of charge via https://www.ccdc.cam.ac.uk/structures/, or by emailing data_request@ccdc.cam.ac.uk, or by contacting The Cambridge Crystallographic Data Centre, 12 Union Road, Cambridge CB2 1EZ, UK; fax: +44 1223 336033.
