# Supplementary material for: Donor-acceptor engineering of a triplet-exciton-optimized MOF photocatalyst for efficient singlet oxygen-mediated oxidation
Source: Natl Sci Rev. 2025 Jan 22;12(4):nwaf024. doi: 10.1093/nsr/nwaf024 (PMC11887853; doi:10.1093/nsr/nwaf024)
Supplement: nwaf024_Supplemental_File [file nwaf024_supplemental_file.pdf]

# Supporting Information

# Donor-acceptor engineering of triplet-exciton-optimized MOF photocatalyst for efficient singlet oxygen-mediated oxidations

Kai Wang[1], Chang-Tai Li[1], Guo-Lei Zhang[1], Han-Yu Wang[1], Lin Geng[1], Bo Zhang[1], Mei-Hui Yu[1], Jijie Zhang[1], Ze Chang[1]*, Xian-He Bu[1,2]*

[1]School of Materials Science and Engineering, TKL of Metal and Molecule-Based Material Chemistry, Nankai University Tianjin 300350

[2]State Key Laboratory of Elemento-Organic Chemistry, College of Chemistry, Nankai University Tianjin 300071

*Corresponding Author(s): changze@nankai.edu.cn; buxh@nankai.edu.cn

# Table of Contents

1) General Information ........................................................................3

2) Synthesis and Characterization of D-A MOFs. ..................................5

3) Photocatalytic Studies .......................................................................31

4) NMR Spectra ......................................................................................48

5) References..........................................................................................588

## 1) General Information

All the reagents and solvents were used without any purification, except tpp ligand, which was synthesized according to previous report.[S1] Unit cell parameters of the crystals were determined with a Rigaku XtalAB Pro MM007 DW diffractometer at 100 K. Powder X-ray diffraction (PXRD) was performed on Rigaku Ultima IV diffractometer (Cu Kα radiation, λ = 1.5406 Å). UV-vis spectra were performed on a Cary 100conc spectrophotometer. Solid- and solution-state luminescence spectra were measured on a Varian Cary Eclipse fluorescence spectrophotometer. Decay curve was measured on Horiba FluoroMax-4 fluorometer with a NanoLED-365 flash lamp. Fourier transform infrared (FT-IR) spectra were collected on a Thermo Scientific Nicolet iS10 spectrophotometer in the range of 4000-400 $cm^{-1}$. Thermogravimetric analysis (TGA) curves were obtained on Mettler-Toledo (TGA/DSC) thermal analyzer from 25 ℃ to 800 ℃ with a heating rate of 10 ℃ $min^{-1}$ under a nitrogen gas atmosphere (20 mL $min^{-1}$). X-ray photoelectron spectroscopy (XPS) data were collected on a Thermofisher scientific K-Alpha+ system with an Al Kα microfocused X-ray source (Energy: 1486.6 eV). Scanning electron microscope (SEM) was performed on FEI Quanta 600 FE.

Electron paramagnetic resonance (EPR) signals were recorded on a Bruker A300 spectrometer (Germany) at room temperature under visible-light irradiation using a white LED. The electrochemical measurements were performed in a conventional three-electrode cell on a CHI-760E electrochemical workstation (Shanghai Chenhua Instrument Co., Ltd, China) with the photocatalyst-coated FTO as the working electrode, Pt plate as the counter electrode, and an Ag/AgCl as a reference electrode. The photoresponsive signals were recorded with a bias potential of 0 V using a 300 W Xe lamp as the light source. The electrolyte was a 0.5 M $Na_2SO_4$ solution. The 2 mg of catalyst was added into 1 mL of EtOH and 10 μL of Nafion mixed solution. Then a 100 μL suspension was dropped on the surface of a FTO glass and dried at room temperature for photocurrent measurements. EIS was performed with a 30 μL suspension on the working electrode in a frequency range from $10^{-1}$ to $10^5$ Hz. $^1$H and

$^{13}C$ NMR spectra were recorded on a Bruker Biospin Avance (400 MHz) equipment using tetramethylsilane (TMS) as an internal standard. The following abbreviations were used to explain the multiplicities: s = singlet, d = doublet, t = triplet, q = quartet, dd = doublet of doublet, dt = doublet of triplet, m = multiplet, Flash column chromatography was performed using Merck silica gel 60 with commercially available solvents.

**$^{1}H$ NMR liquid measurements of the digested host-guest MOFs with different feeding amounts of guest.**

Here, we provide a method to determine the ratio between tpp, the loading guest and TPA trough digested crystals by $^{1}H$ liquid NMR measurements. The crystal samples were digested with a mixture of hydrochloric acid (DCl) and DMSO-$d_6$ for test. The corresponding ligand and guest components were dissolved with the same mixture and tested as standards for quantitative analysis. In consideration of the confined inner space of the cage in ($\{[Cd_3(tpp)_2(TPA)_3(H_2O)_3]\}_n$) and the dimension of Icz molecules, each cage can accommodate only one guest molecule. When the cages were totally occupied by the guests, the loading proportion of guests could be defined as 100%. Taking account of the formula of ($\{[Cd_3(tpp)_2(TPA)_3(H_2O)_3]\}_n$), the total loading would be resulted in a guest : tpp mole ratio with 1:2. Through calculating the integral area of the characteristic peaks, the actual ratio of tpp and guest molecules involved in the crystal samples could be gained. Then the guest loading in percentage form could be obtained based on the following translation:

$$\text{Guest loading (\%)} = \frac{\text{Experimental guest to tpp mole ratio}}{\text{Theoretical guest to tpp mole ratio}} \times 100\%$$

The calculation method of the ratio between tpt, the loading guest and TPA is almost identical to the above method, the only difference is that tpt is used instead of tpp.

## 2) Synthesis and Characterization of D-A MOFs

**Preparation of C1**, **D1**, **E1** and **F1**: A mixture of $Cd(NO_3)_2•4H_2O$ (15.4 mg, 0.05 mmol), tpp (7.5 mg, 0.025 mmol), Carboxylate ligands($H_2TPA$ (8.3 mg, 0.05 mmol) or 2-Br$H_2$TPA (12.2 mg, 0.05 mmol)), guest (Icz (12.5 mg, 0.05 mmol) or 2-BrIcz (12.8 mg, 0.04 mmol)), DMF (4 mL), $H_2O$ (4 mL), and $C_2H_5OH$ (4 mL) were sealed in a 20 mL vial and heated at 95 $^oC$ for 1 day. The mixture was then cooled down to room temperature. The hexagonal rod crystals were filtered and washed with solvents (DMF, $H_2O$ and ethanol), then dried in a vacuum oven at 80 $^oC$ for 12 h. (**C1**: green color; **D1**: light-green color; **E1**: green color; **F1**: light-green color).

**Preparation of Icz@NKU-111:** A mixture of $Cd(NO_3)_2•4H_2O$ (15.4 mg, 0.05 mmol), tpt (7.7 mg, 0.025 mmol), Carboxylate ligands($H_2TPA$ (8.3 mg, 0.05 mmol), guest (Icz (12.5 mg, 0.05 mmol), DMF (4 mL), $H_2O$ (4 mL), and $C_2H_5OH$ (4 mL) were sealed in a 20 mL vial and heated at 95 $^oC$ for 1 day. The mixture was then cooled down to room temperature. The hexagonal rod crystals were filtered and washed with solvents (DMF, $H_2O$ and ethanol), then dried in a vacuum oven at 80 $^oC$ for 12 h. (**Icz@NKU-111**: yellow color).

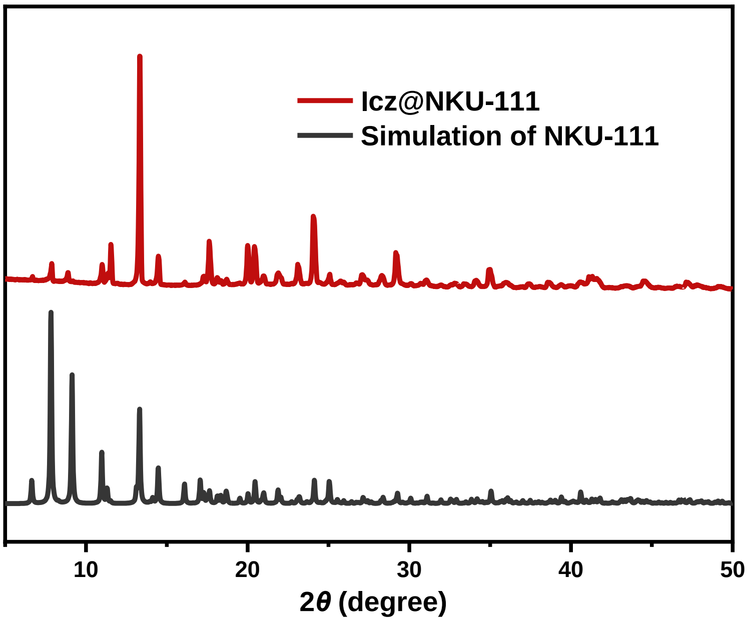


Figure S1. PXRD patterns of **Icz@NKU-111**.

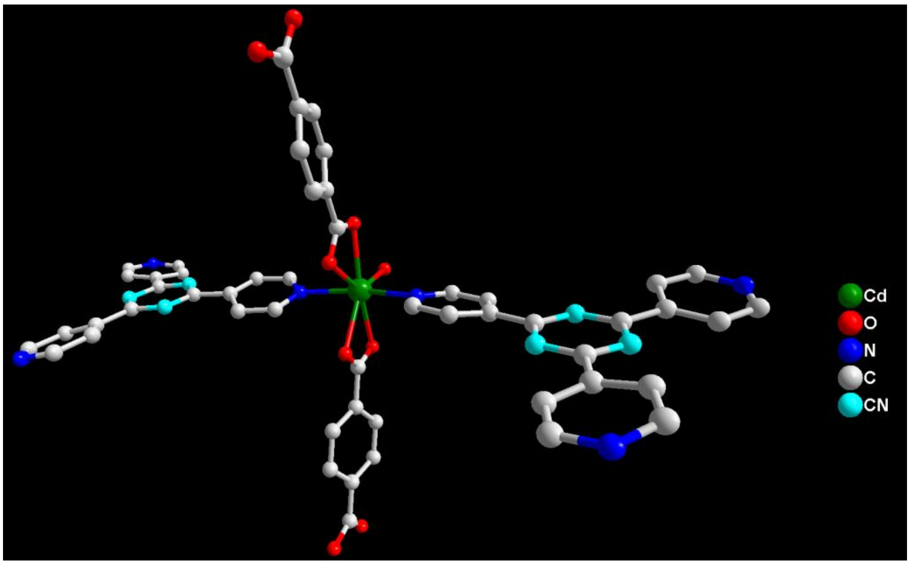


Figure S2. Coordination environment of Cd(II) ions and ligands in **C1**.

| D-A MOFs | Chemical Formula | Guest | L1 |
|---|---|---|---|
| **C1** | {[$Cd_3$(tpp)$_2$(**L1**)$_3$($H_2O$)$_3$]·2$H_2O$}$_n$ | Icz | TPA |
| **D1** | | 2-BrIcz | TPA |
| **E1** | | Icz | 2-BrTPA |
| **F1** | | 2-BrIcz | 2-BrTPA |
| **Icz@NKU-111** | {[$Cd_3$(tpt)$_2$(**L1**)$_3$($H_2O$)$_3$]·2$H_2O$}$_n$ | Icz | TPA |

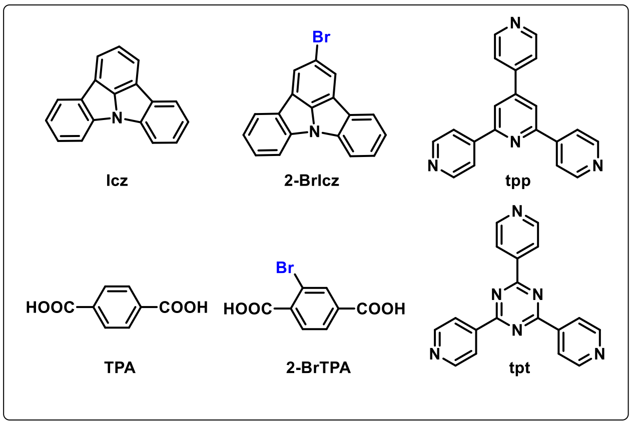


Figure S3. The composition of **C1**, **D1**, **E1** and **F1**.

**Table S1**. Crystal structures refinement details.

| C1 | |
|---|---|
| Empirical formula | $C_{438}H_{333}Cd_{18}N_{51}O_{102}$ |
| Formula weight | 9965.73 |
| Temperature (K) | 193 |
| Wavelength (Å) | 1.34139 |
| Crystal system | Trigonal |
| Space group | *P*-3*c*1 |
| a (Å) | 26.7403(6) |
| b (Å) | 26.7403(6) |
| c (Å) | 21.2619(6) |
| α (°) | 90 |
| β (°) | 90 |
| γ (°) | 120 |
| V (Å$^3$) | 13166.3(7) |
| Z | 1 |
| $D_{calc}$ (g m$^{-3}$) | 1.257 |
| μ (mm$^{-1}$) | 4.261 |
| F (000) | 4998 |
| θ (º) | 2.88 to 58.62 |
| GOF | 1.050 |
| $R_1$ (I>2sigma(I))[a] | 0.0784 |
| $wR_2$ (all data)[b] | 0.2699 |
| CCDC | 2306707 |

| | **D1** |
|---|---|
| Empirical formula | $C_{492}H_{360}Br_6Cd_{18}N_{54}O_{102}$ |
| Formula weight | 11163 |
| Temperature (K) | 193 |
| Wavelength (Å) | 0.71073 |
| Crystal system | Trigonal |
| Space group | *P*-3 |
| a (Å) | 26.7779(10) |
| b (Å) | 26.7779(10) |
| c (Å) | 21.2728(12) |
| α (°) | 90 |
| β (°) | 90 |
| γ (°) | 120 |
| V ($Å^3$) | 13210.2(12) |
| Z | 1 |
| $D_{calc}$ (g $m^{-3}$) | 1.403 |
| μ ($mm^{-1}$) | 1.238 |
| F (000) | 5580 |
| θ (º) | 2.51 to 27.10 |
| GOF | 1.032 |
| $R_1$ (I>2sigma(I))[a] | 0.0983 |
| $wR_2$ (all data)[b] | 0.1974 |
| CCDC | 2308341 |

| | E1 |
|---|---|
| Empirical formula | $C_{456}H_{326}Br_{18}Cd_{18}N_{52}O_{102}$ |
| Formula weight | 11627 |
| Temperature (K) | 193 |
| Wavelength (Å) | 0.71073 |
| Crystal system | Trigonal |
| Space group | *P*-3*c*1 |
| a (Å) | 27.070(16) |
| b (Å) | 27.070(16) |
| c (Å) | 21.526(14) |
| α (°) | 90 |
| β (°) | 90 |
| γ (°) | 120 |
| V ($Å^3$) | 13661(18) |
| Z | 1 |
| $D_{calc}$ ($g\ m^{-3}$) | 1.413 |
| μ ($mm^{-1}$) | 2.072 |
| F (000) | 5736.0 |
| θ (º) | 2.47 to 22.36 |
| GOF | 1.045 |
| $R_1$ (I>2sigma(I))[a] | 0.0878 |
| $wR_2$ (all data)[b] | 0.2759 |
| CCDC | 2314760 |

| | F1 |
|---|---|
| Empirical formula | $C_{492}H_{318}Br_{24}Cd_{18}N_{54}O_{90}$ |
| Formula weight | 12367 |
| Temperature (K) | 193 |
| Wavelength (Å) | 1.34139 |
| Crystal system | Trigonal |
| Space group | *P*-3*c*1 |
| a (Å) | 26.771(2) |
| b (Å) | 26.771(2) |
| c (Å) | 21.306(2) |
| α (°) | 90 |
| β (°) | 90 |
| γ (°) | 120 |
| V ($Å^3$) | 13224(3) |
| Z | 1 |
| $D_{calc}$ (g m$^{-3}$) | 1.553 |
| μ ($mm^{-1}$) | 5.674 |
| F (000) | 6072.0 |
| θ (º) | 2.87 to 56.82 |
| GOF | 1.022 |
| $R_1$ (I>2sigma(I))[a] | 0.0883 |
| $wR_2$ (all data)[b] | 0.2925 |
| CCDC | 2322732 |

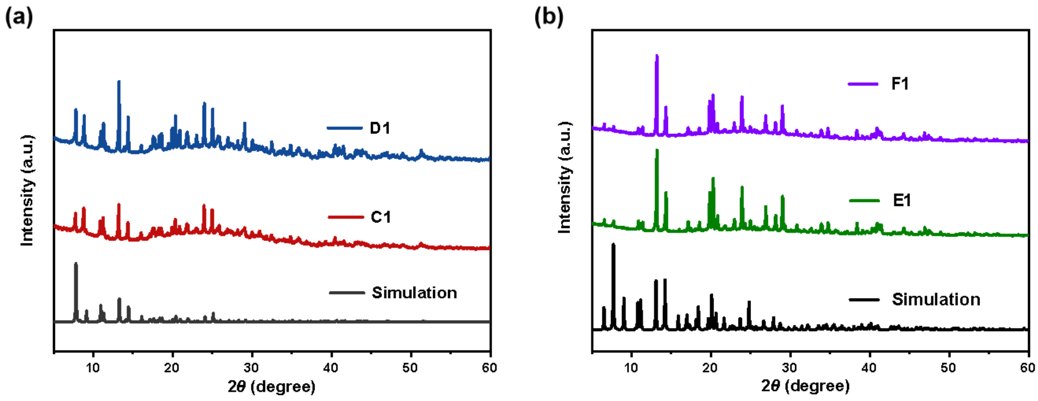


Figure S4. PXRD patterns of (a) **C1** and **D1**; (b) **E1** and **F1**.

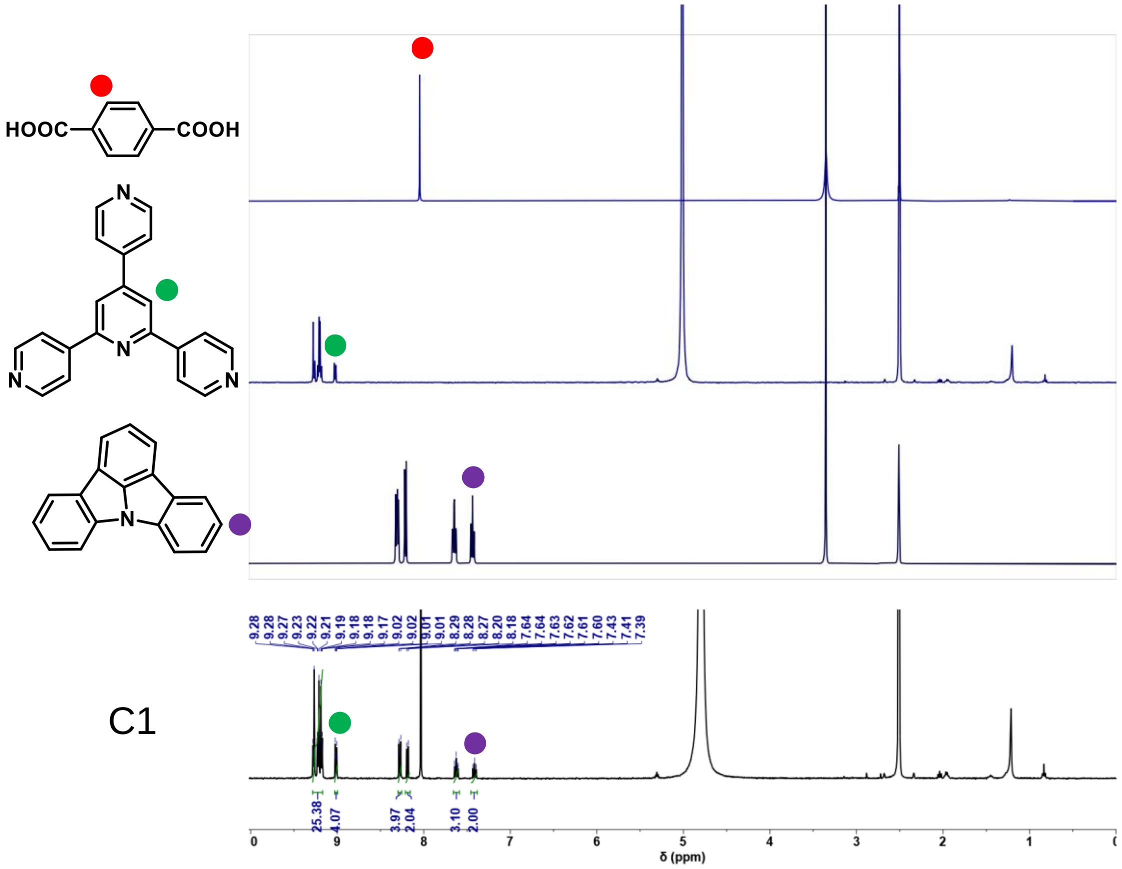


| | Feeding amount of guest | Experimental $^1$H mole ratio of guest to tpp (from $^1$H Integral area) | Experimental guest to tpp mole ratio | Loading ratio of guest |
|---|---|---|---|---|
| **C1** | 0.05 | 2.00:4.07 | 1:2.035 | 98.28% |

Figure S5. $^1$H liquid NMR spectra of **C1** and the corresponding ligands and guest.

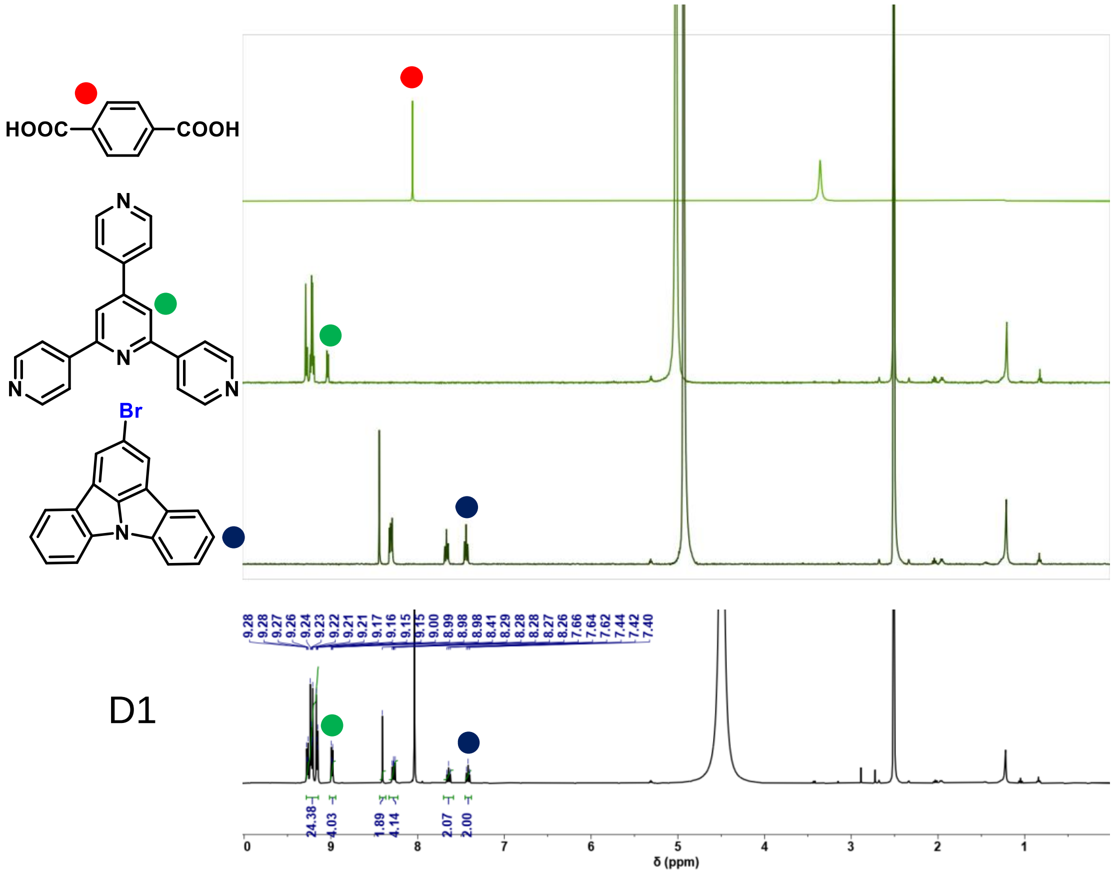


| | Feeding amount of guest | Experimental $^{1}$H mole ratio of guest to tpp (from $^{1}$H Integral area) | Experimental guest to tpp mole ratio | Loading ratio of guest |
|---|---|---|---|---|
| **D1** | 0.04 | 2.00:4.03 | 1:2.015 | 99.26% |

Figure S6. $^{1}$H liquid NMR spectra of **D1** and the corresponding ligands and guest.

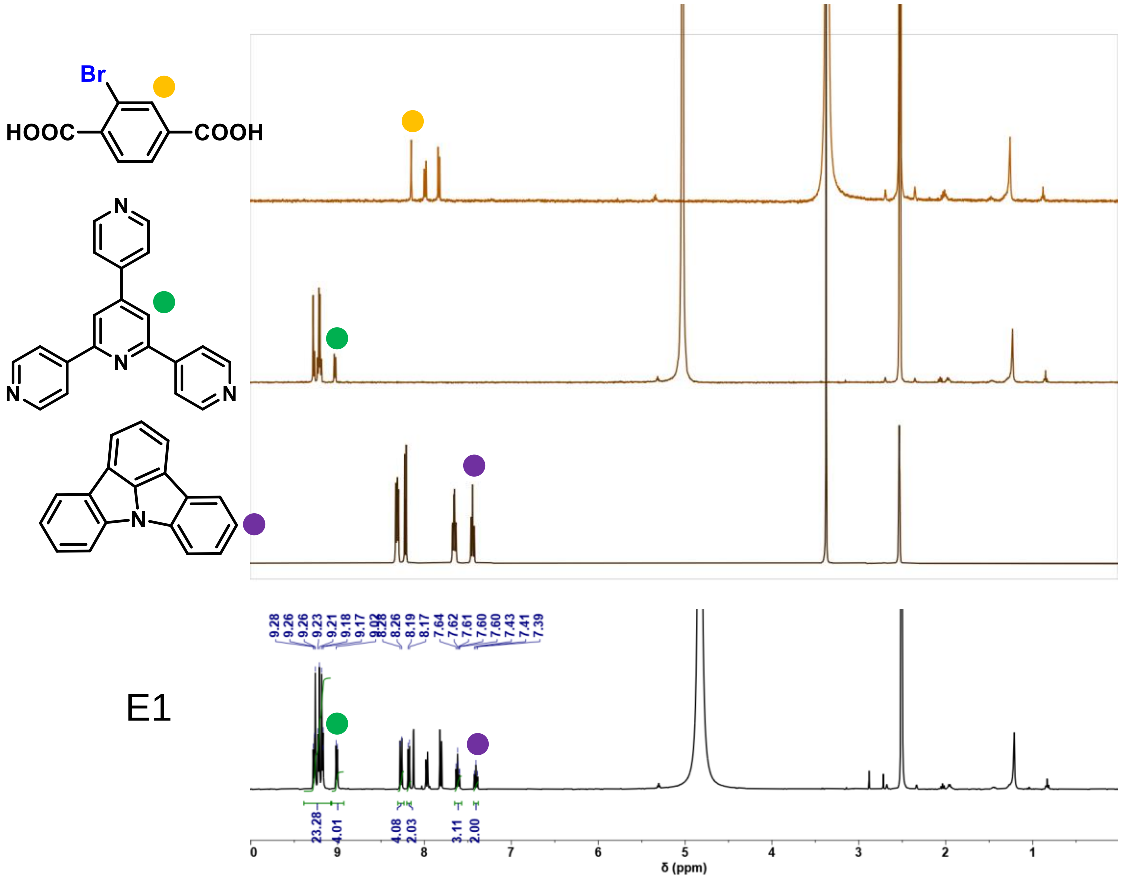


| | Feeding amount of guest | Experimental $^{1}$H mole ratio of guest to tpp (from $^{1}$H Integral area) | Experimental guest to tpp mole ratio | Loading ratio of guest |
|---|---|---|---|---|
| **E1** | 0.05 | 2.00:4.01 | 1:2.005 | 99.75% |

Figure S7. $^{1}$H liquid NMR spectra of **E1** and the corresponding ligands and guest.

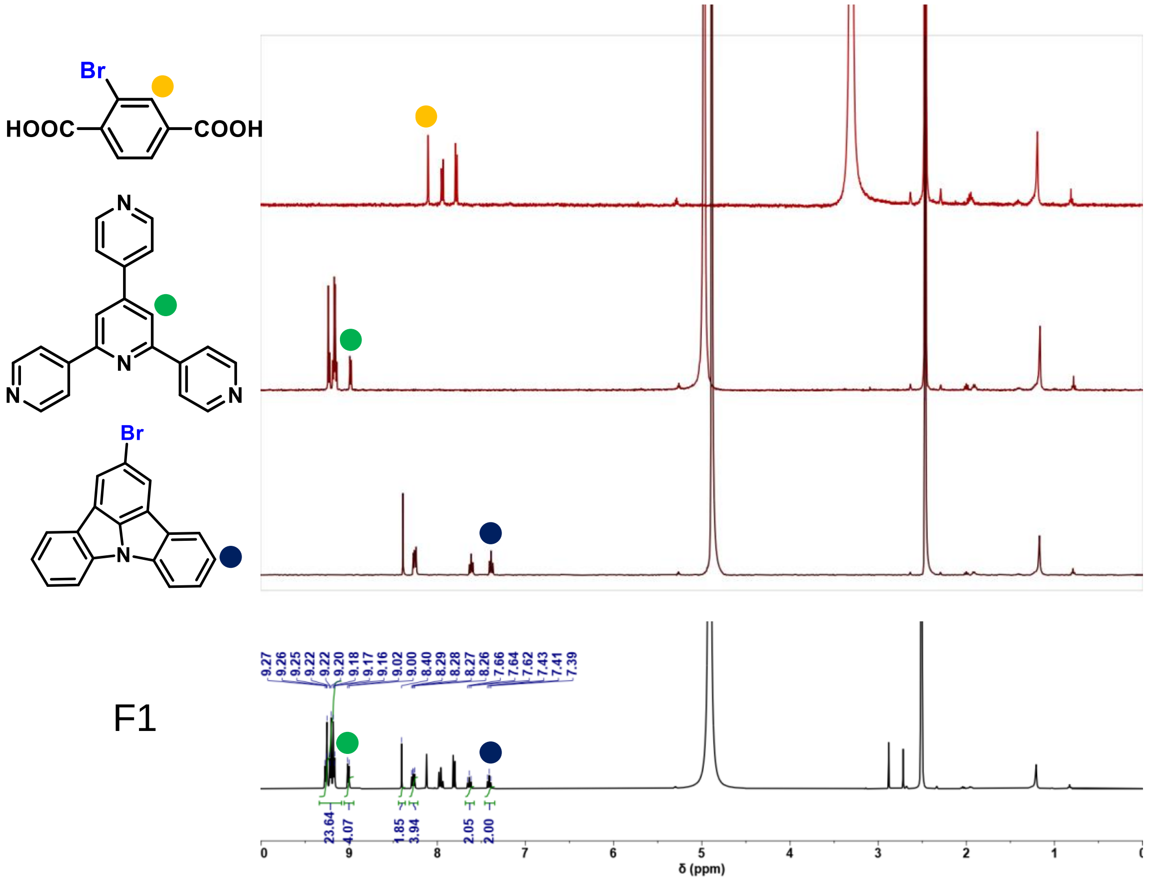


| | Feeding amount of guest | Experimental $^1$H mole ratio of guest to tpp (from $^1$H Integral area) | Experimental guest to tpp mole ratio | Loading ratio of guest |
|---|---|---|---|---|
| **F1** | 0.04 | 2.00:4.07 | 1:2.035 | 98.28% |

Figure S8. $^1$H liquid NMR spectra of **F1** and the corresponding ligands and guest.

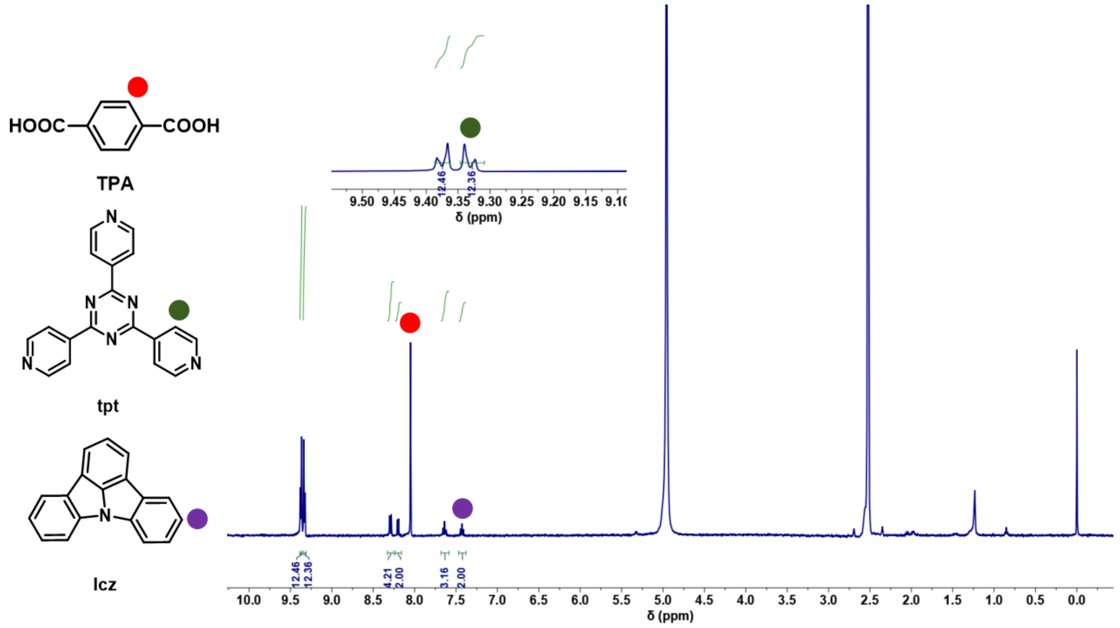


| | Feeding amount of guest | Experimental $^{1}H$ mole ratio of guest to tpt (from $^{1}H$ Integral area) | Experimental guest to tpt mole ratio | Loading ratio of guest |
|---|---|---|---|---|
| **Icz@NKU-111** | 0.05 | 2.00:12.36 | 1:2.060 | 97.09% |

Figure S9. $^{1}H$ liquid NMR spectra of **Icz@NKU-111** and the corresponding ligands and guest.

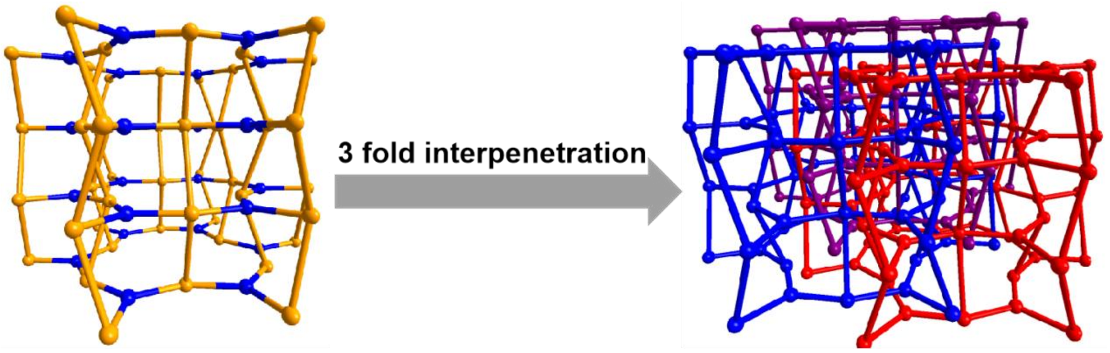


Figure S10. The 3,4-connected tfz topology of the interpenetrated network. The three and four coordinated nodes are presented in blue and yellow sphere, respectively. (b) the three-fold interpenetrated framework of **C1** to **F1**.

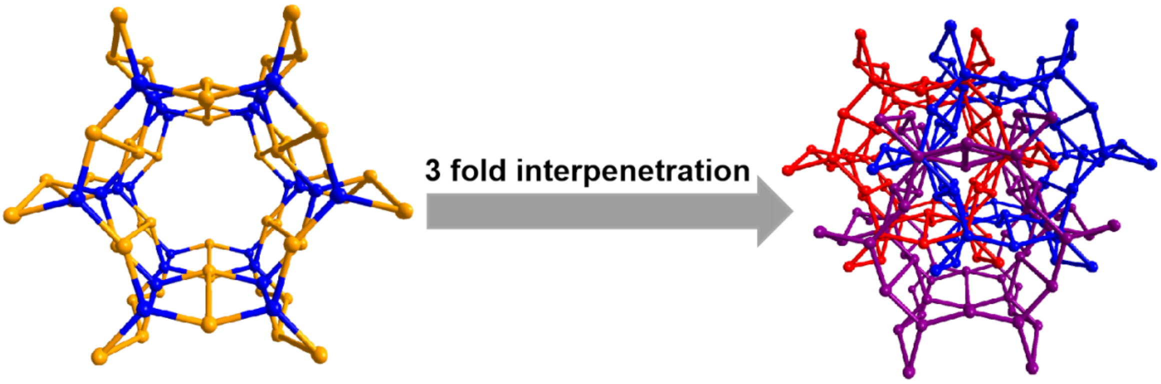


Figure S11. View of the threefold interpenetrated framework along the c-axis.

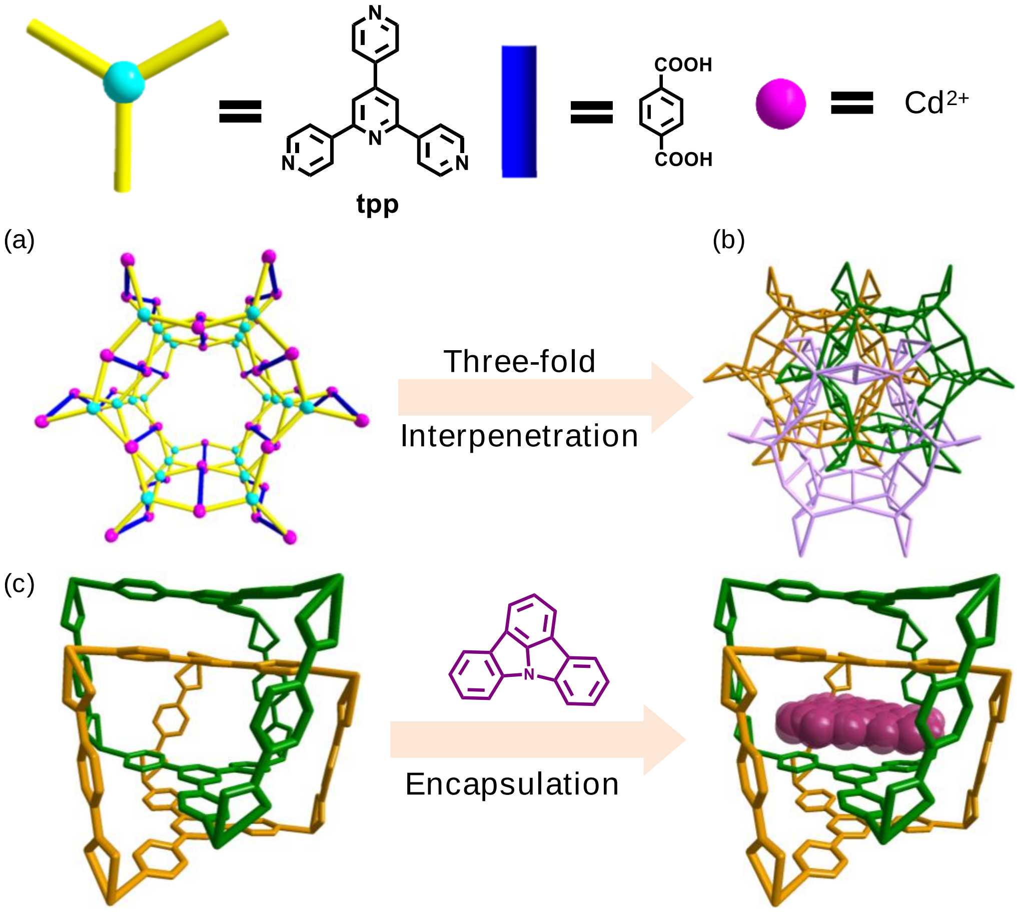


Figure S12. Structures of **C1**. (a) View of the individual network. (b) View of the three-fold interpenetrated framework. (c) The guest accessible space in the triangular prism-shaped cage from two interlocked networks of **C1**.

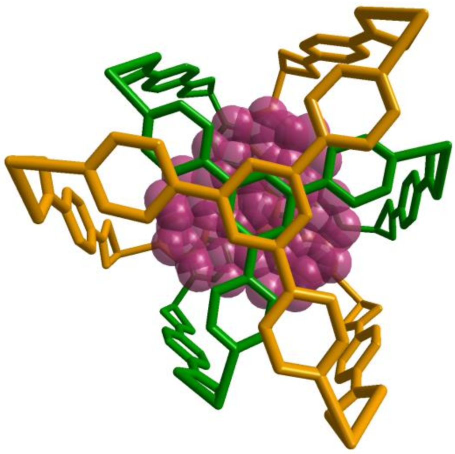

Figure S13. The hexagonal prism cages of **C1** containing the Icz molecules in view of c axis.

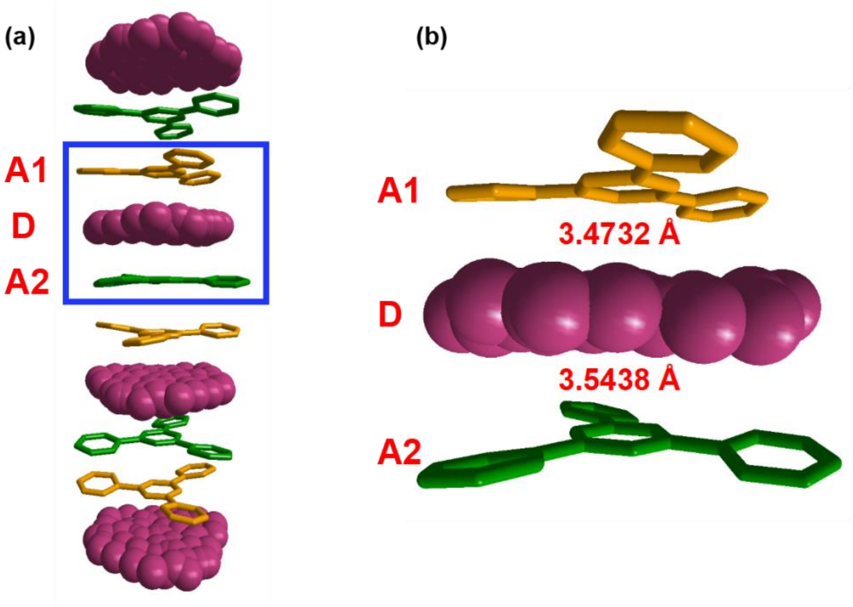


Figure S14. (a) The stacking of D (Icz) and A (tpp) in **C1**. (b) The central distances of A1-D and A2-D (bottom) in crystals **C1**.

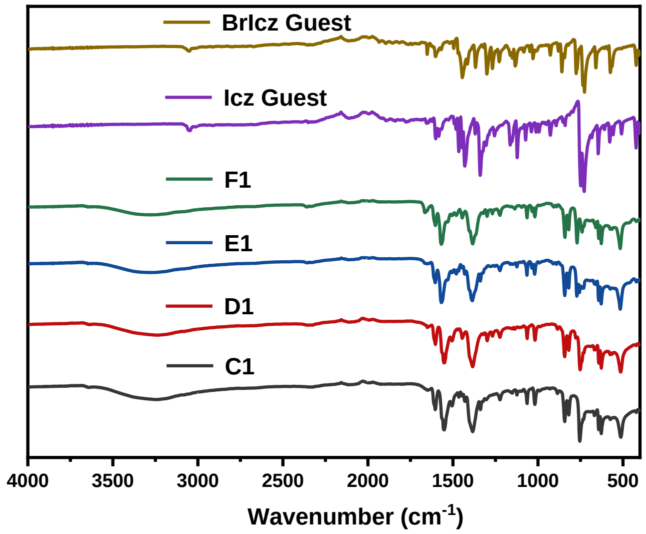


Figure S15. IR spectra of **C1 - F1** series compounds.

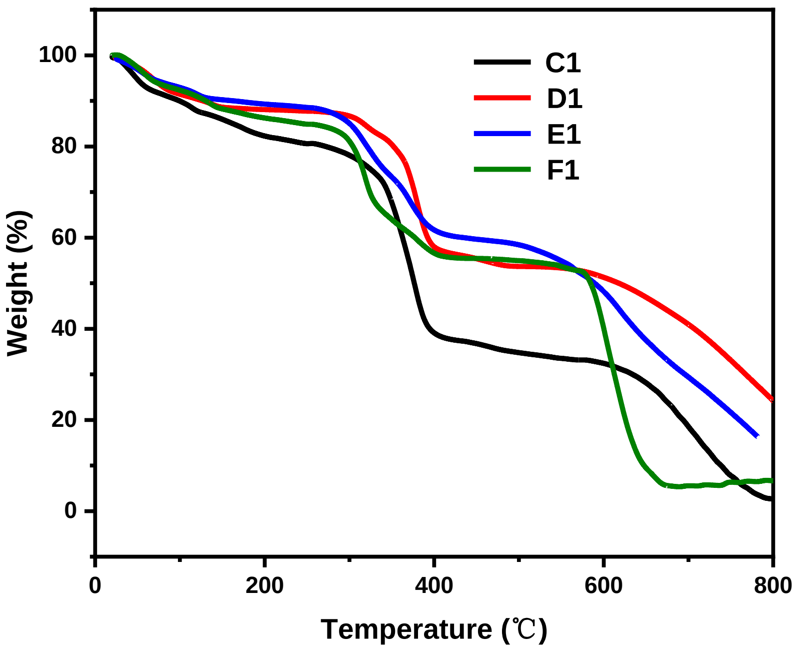


Figure S16. TGA profiles of **C1-F1** series compounds.

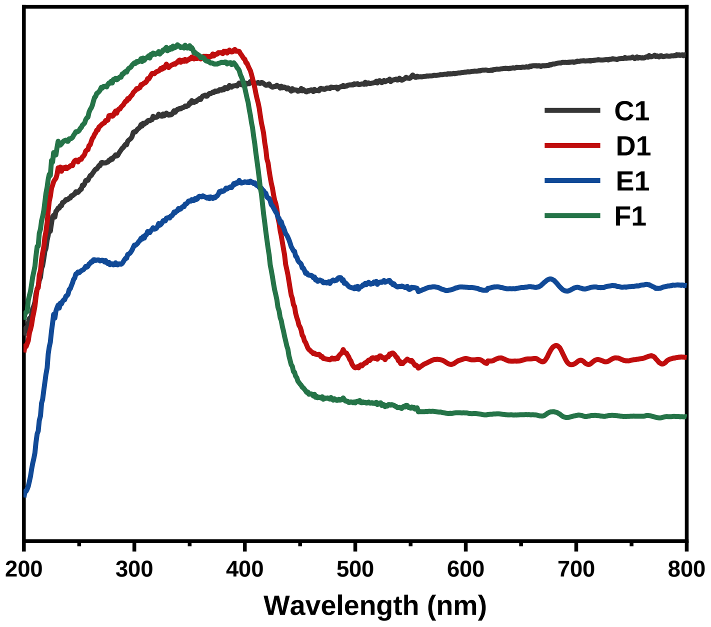


Figure S17. Solid-state UV-vis absorption spectra of **C1-F1** series compounds.

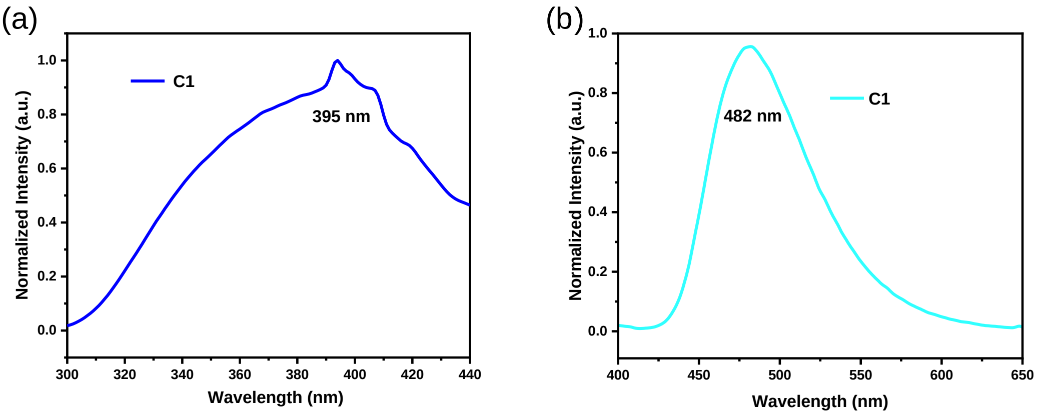


Figure S18. (a) The excitation spectra of **C1**. (b) The emission spectra of **C1**.

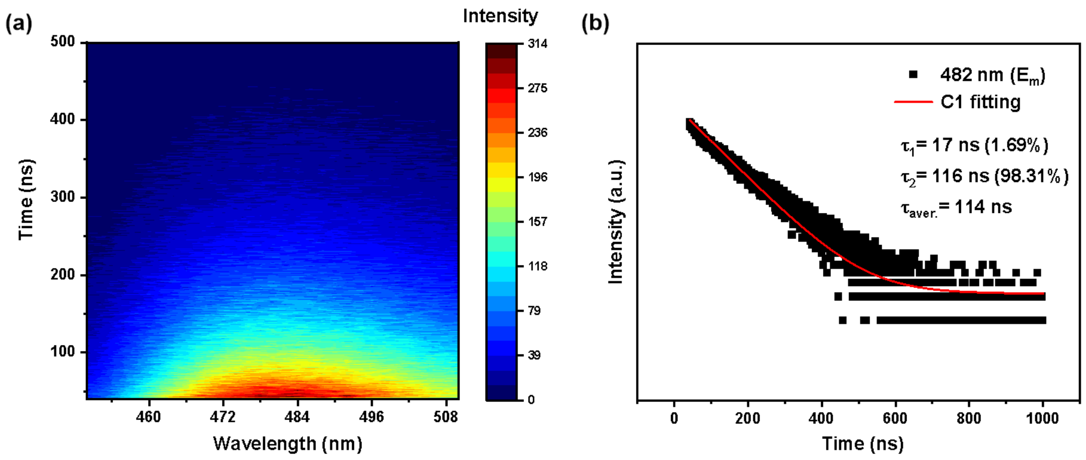


Figure S19. (a) Time-resolved emission spectra of **C1** from 450 nm to 510 nm at 298 K. (b) Fit for the PL decay curve of **C1** ($E_m$ = 482 nm).

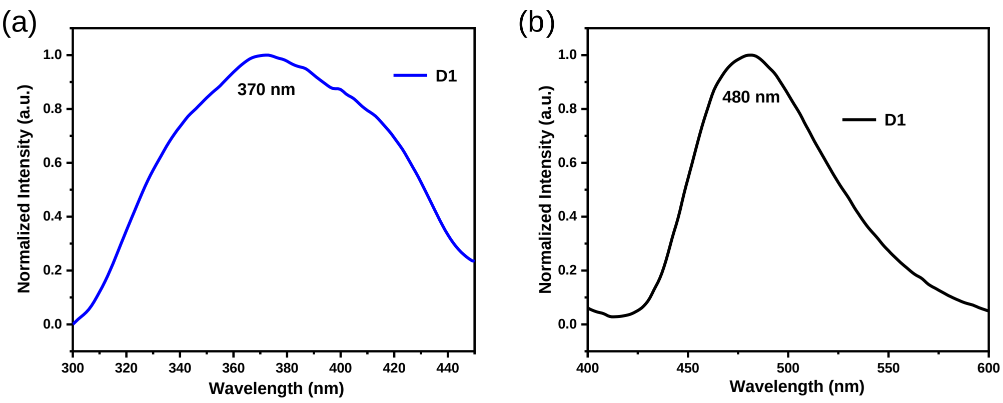


Figure S20. (a) The excitation spectra and (b) the emission spectra of **D1**.

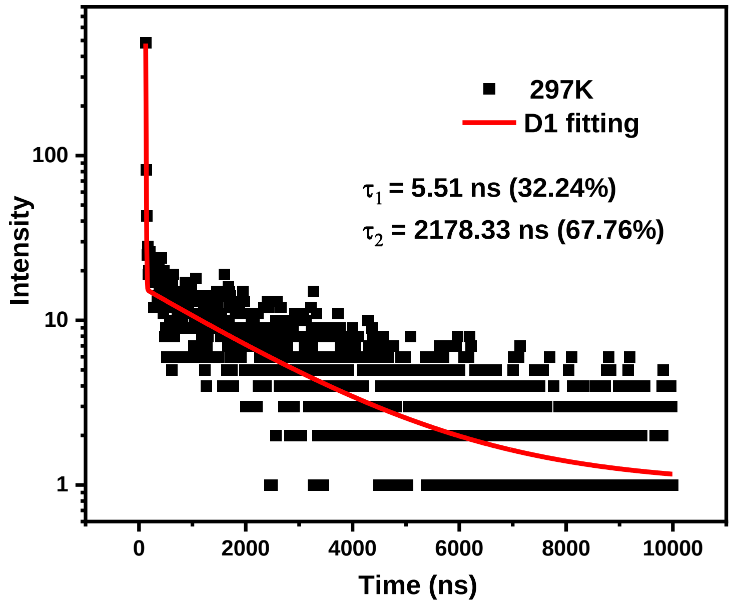


Figure S21. The PL decay curve of **D1** at room temperature (297 K).

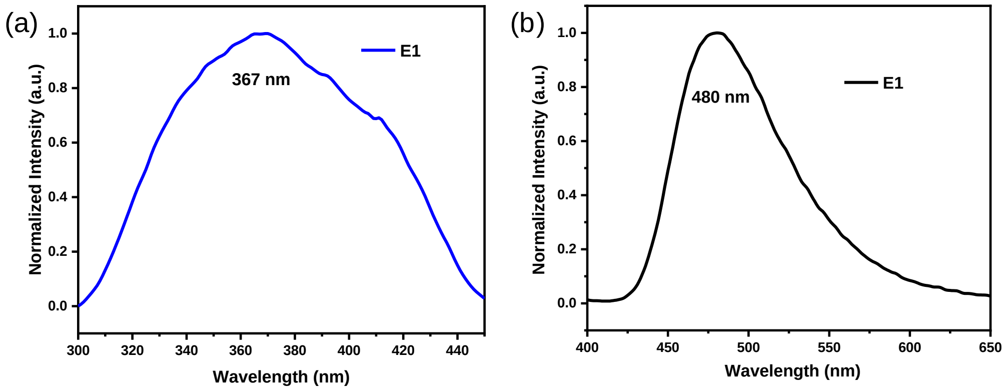


Figure S22. (a) The excitation spectra and (b) the emission spectra of **E1**.

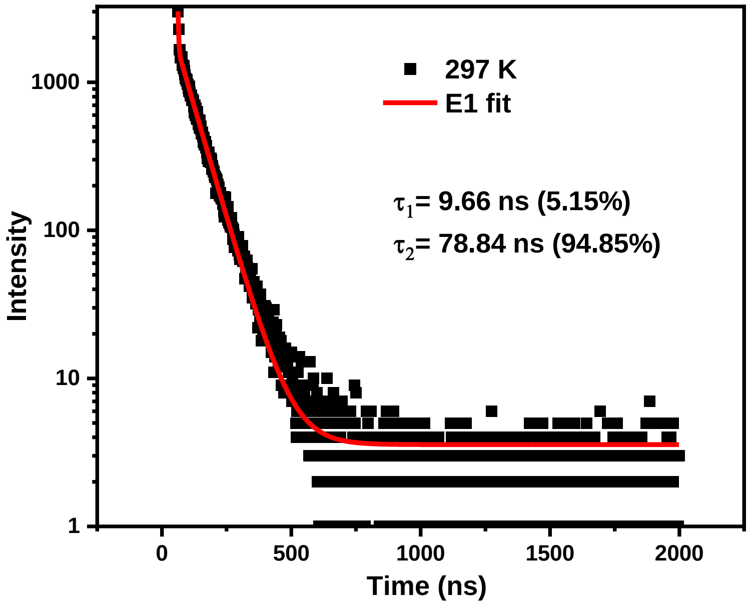


Figure S23. The PL decay curve of **E1** at room temperature (297 K).

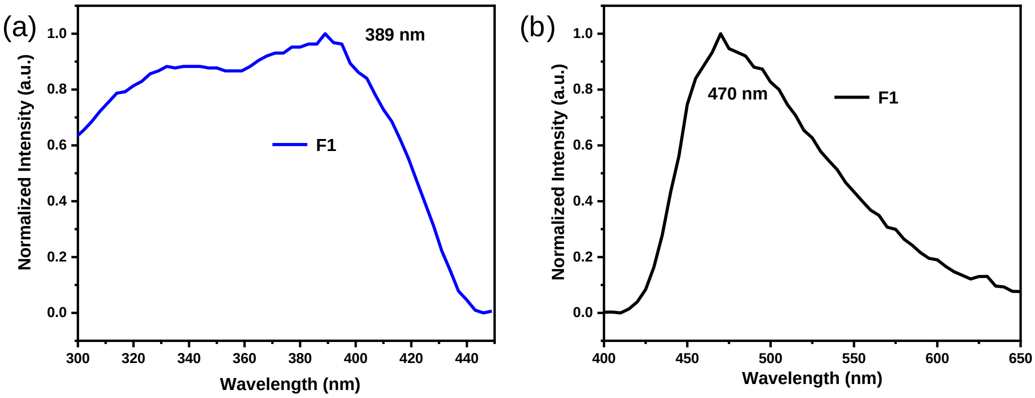


Figure S24. (a) The excitation spectra and (b) the emission spectra of **F1**.

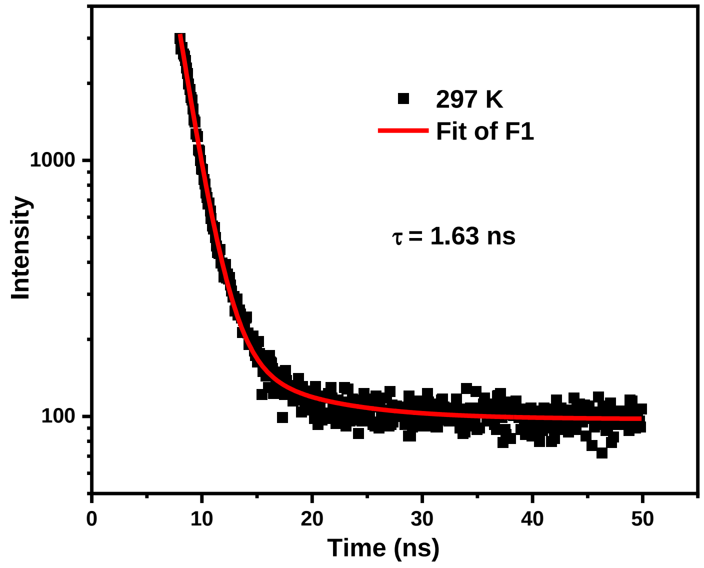


Figure S25. The PL decay curve of **F1** at room temperature (297 K).

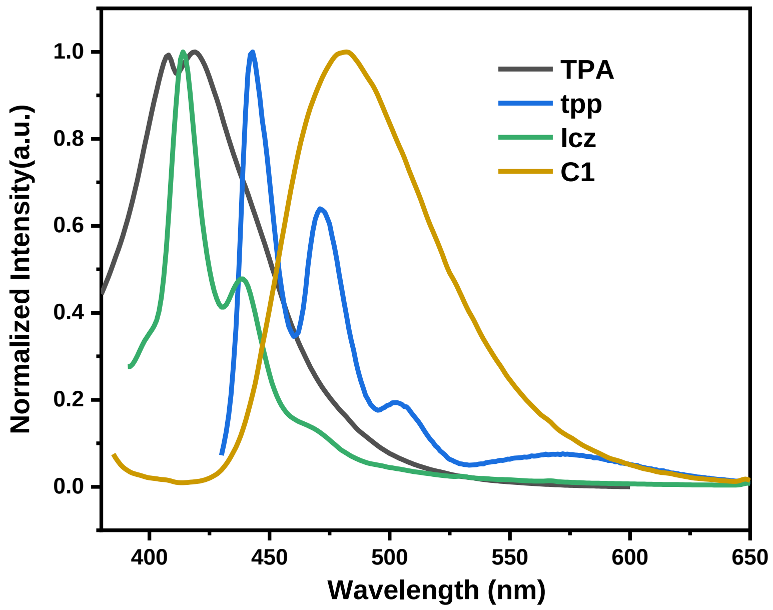


Figure S26. The PL spectra of TPA ligand, tpp ligand, Icz guest and **C1** at room temperature.

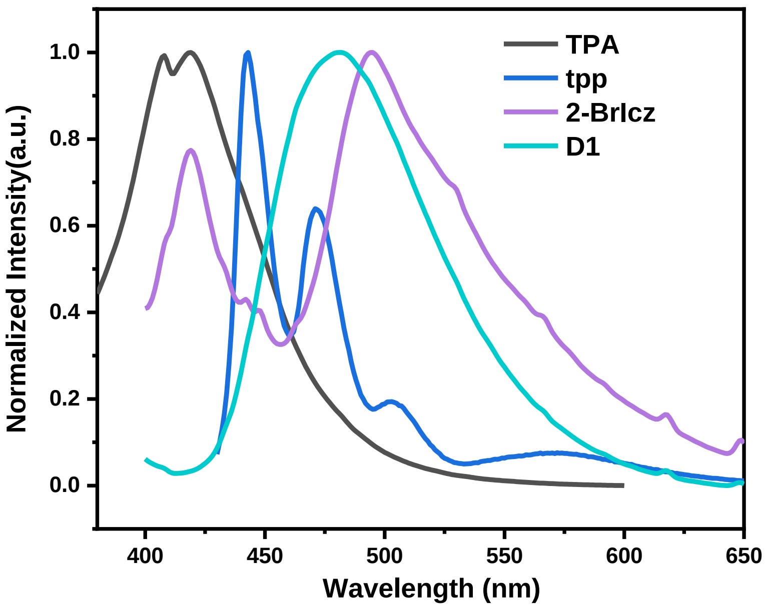


Figure S27. The PL spectra of TPA ligand, tpp ligand, 2-BrIcz guest and **D1** at room temperature.

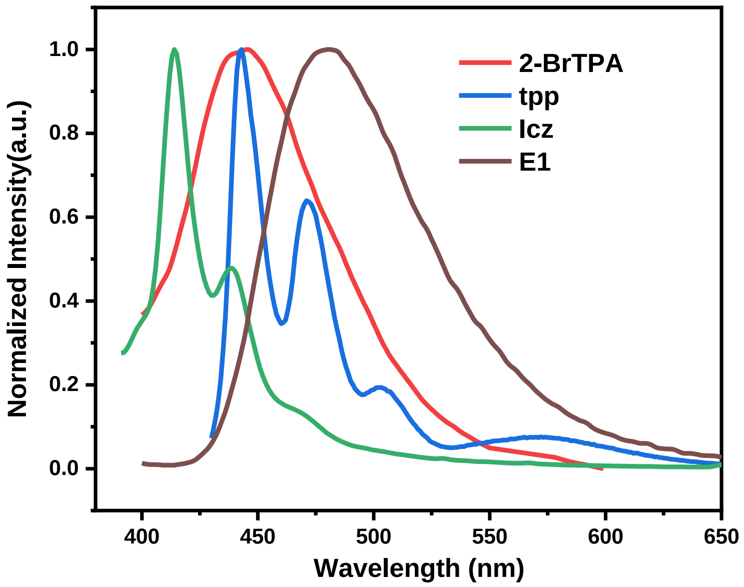


Figure S28. The PL spectra of 2-BrTPA ligand, tpp ligand, Icz guest and **E1** at room temperature.

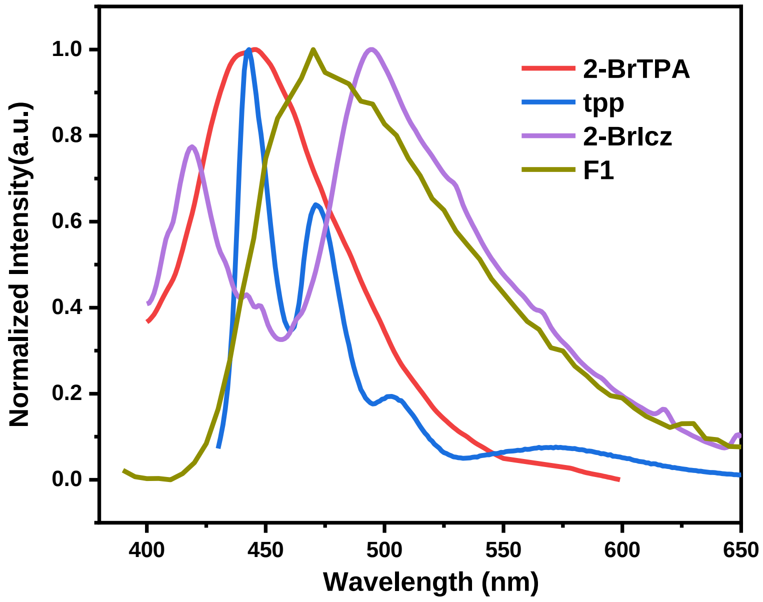


Figure S29. The PL spectra of 2-BrTPA ligand, tpp ligand, 2-BrIcz guest and **F1** at room temperature.

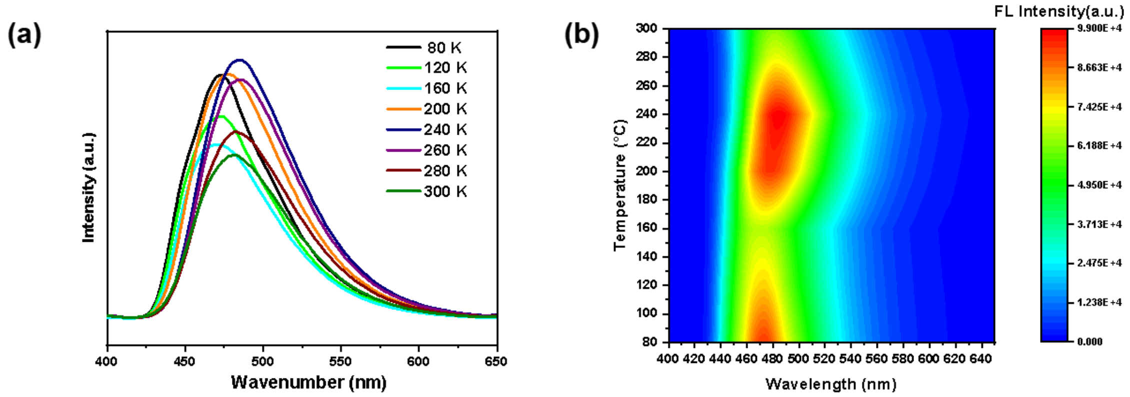


Figure S30. (a) PL spectra of **C1** at different temperatures. (b) Trend of luminescence intensity of **C1** with temperature.

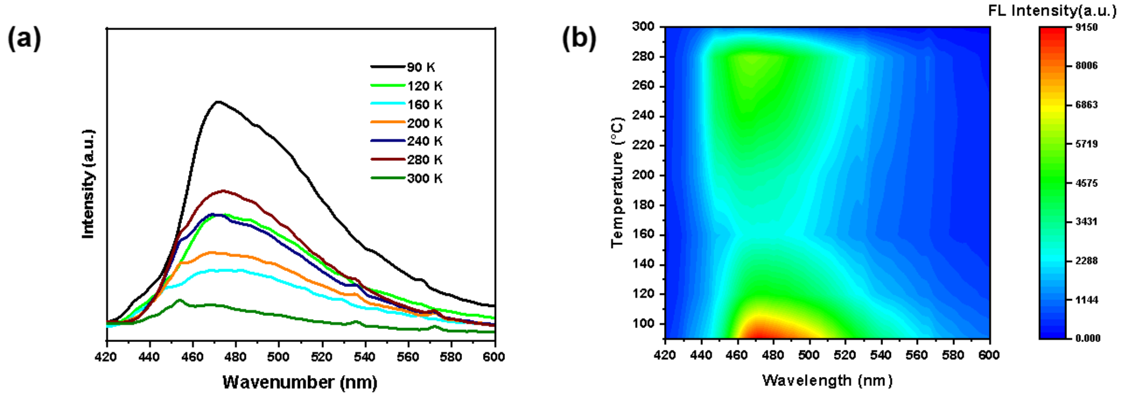


Figure S31. (a) PL spectra of **D1** at different temperatures. (b) Trend of luminescence intensity of **D1** with temperature.

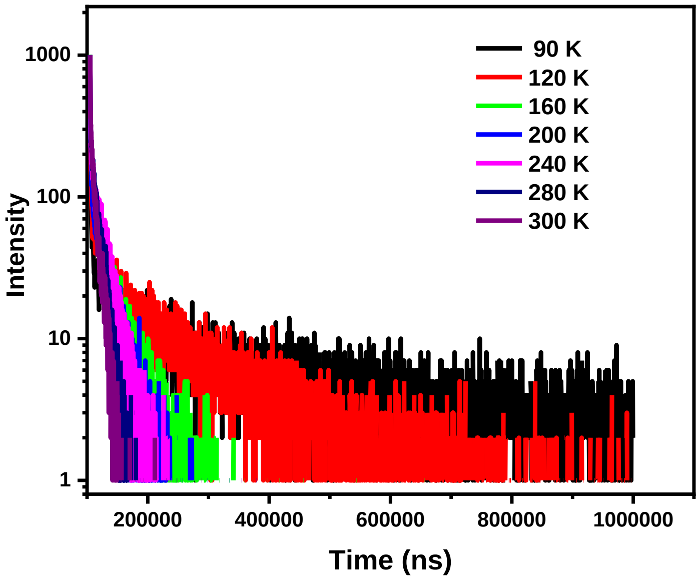


Figure S32. Temperature-dependent PL decay curves of **D1**.

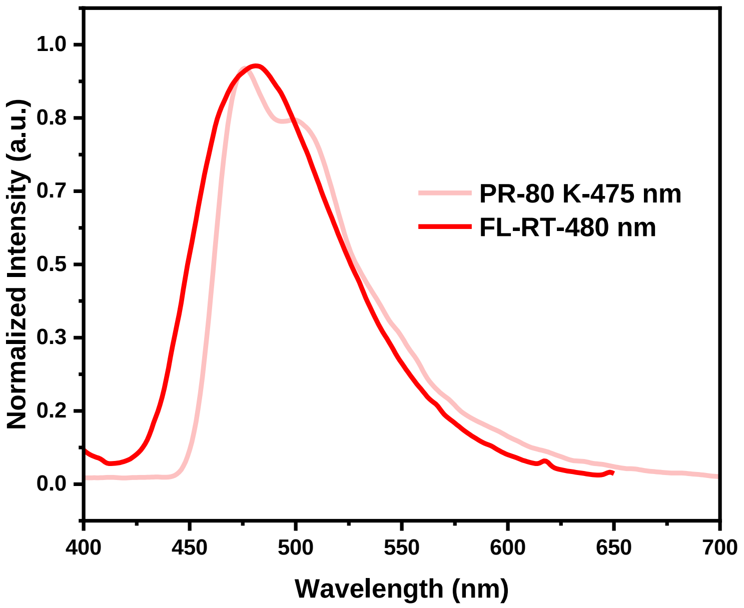


Figure S33. The phosphorescence (PR) spectra at 77 K and fluorescence (FL) spectra at room temperature (RT) of **D1**.

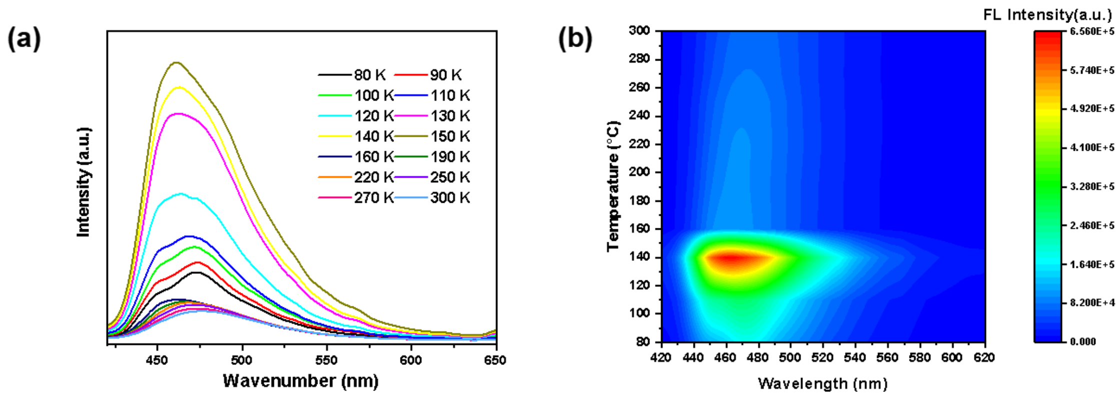


Figure S34. (a) PL spectra of **E1** at different temperatures. (b) Trend of luminescence intensity of **E1** with temperature.

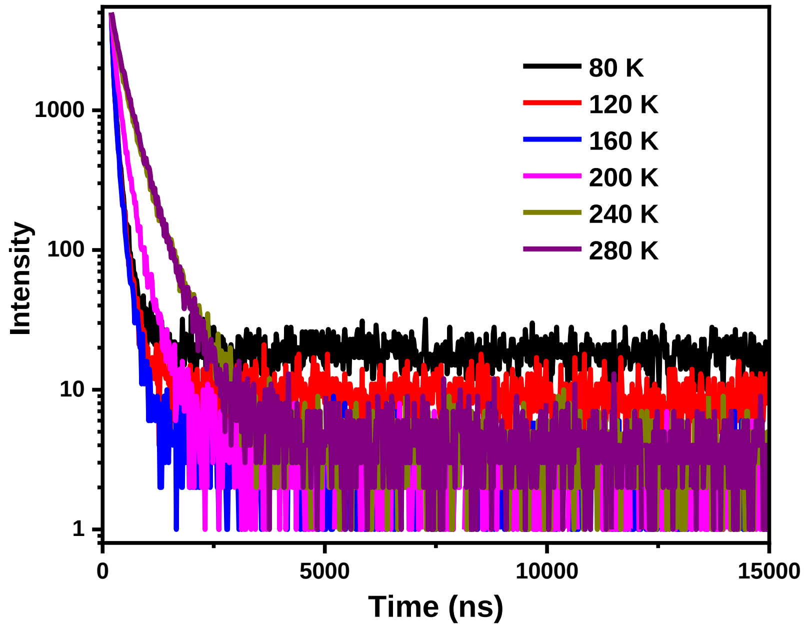


Figure S35. Temperature-dependent PL decay curves of **E1**.

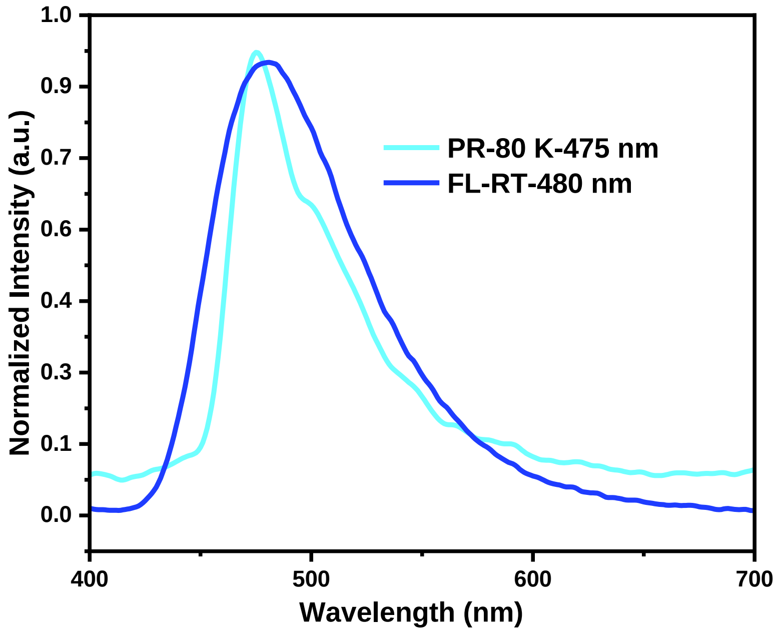


Figure S36. The phosphorescence (PR) spectra at 77 K and fluorescence (FL) spectra at room temperature (RT) of **E1**.

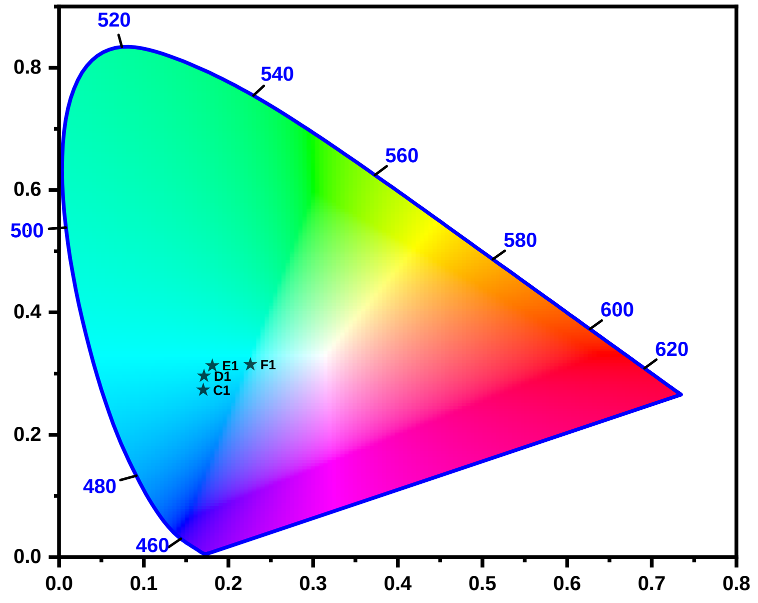


Figure S37. The CIE color coordinates of the emissions of **C1** to **F1**.

## 3) Photocatalytic Studies

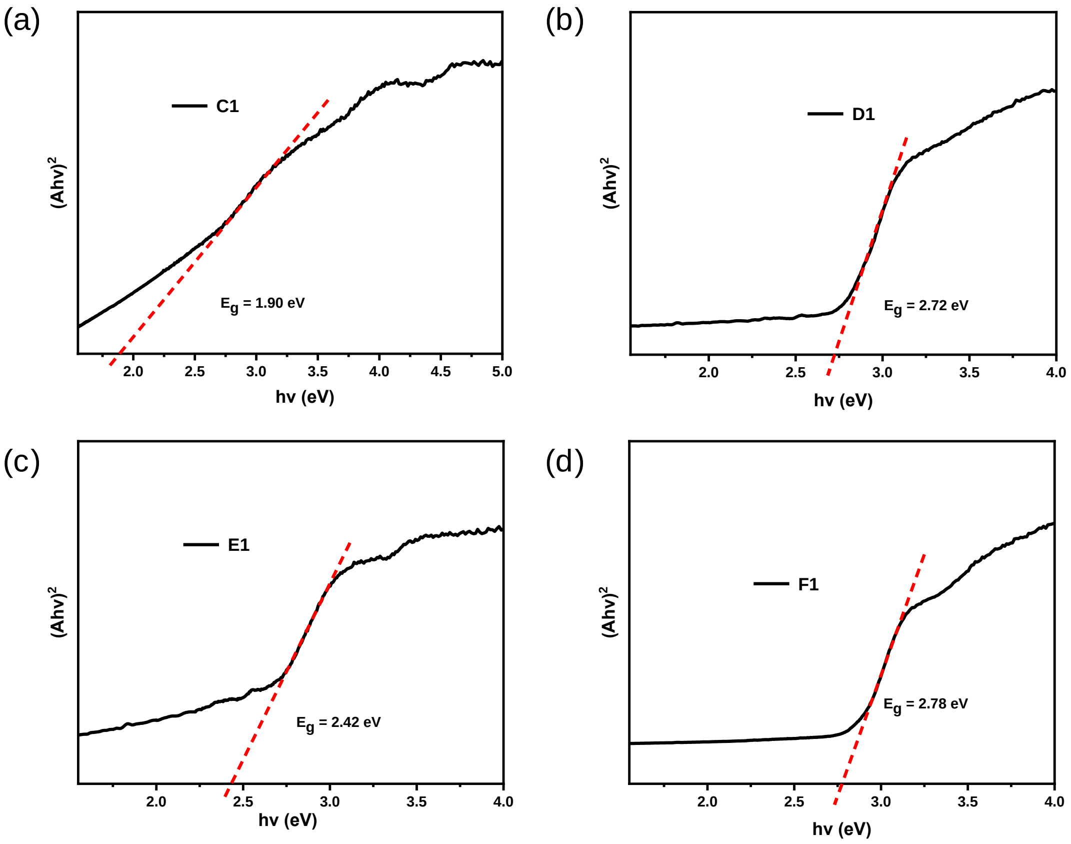


Figure S38. Tauc plot for (a) **C1**, (b) **D1**, (c) **E1** and (d) **F1**.

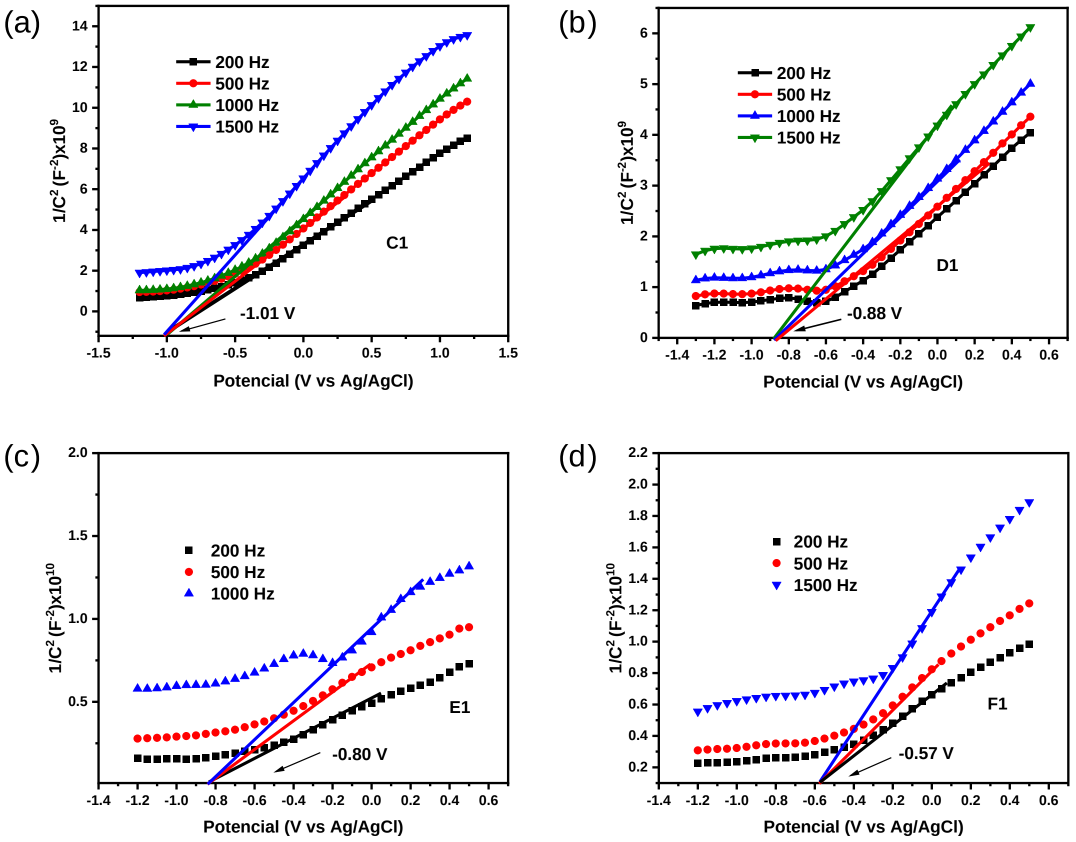


Figure S39. Mott-Schottky plots for (a) **C1**, (b) **D1**, (c) **E1** and (d) **F1**.

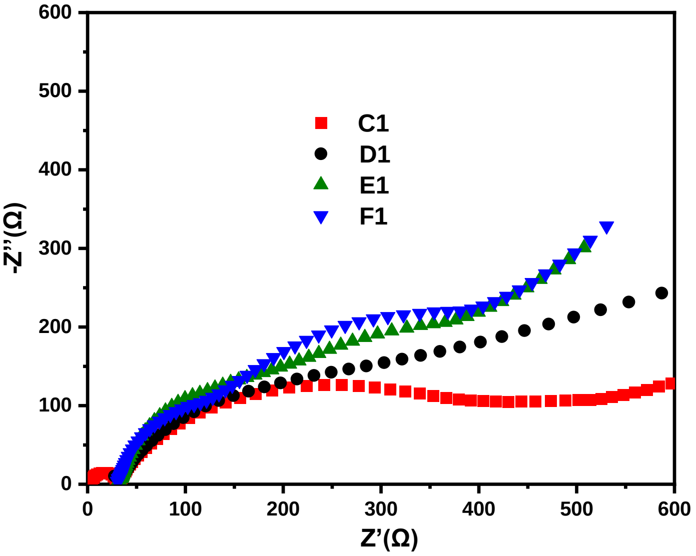


Figure S40. EIS Nyquist plots of the **C1 - F1** series compounds.

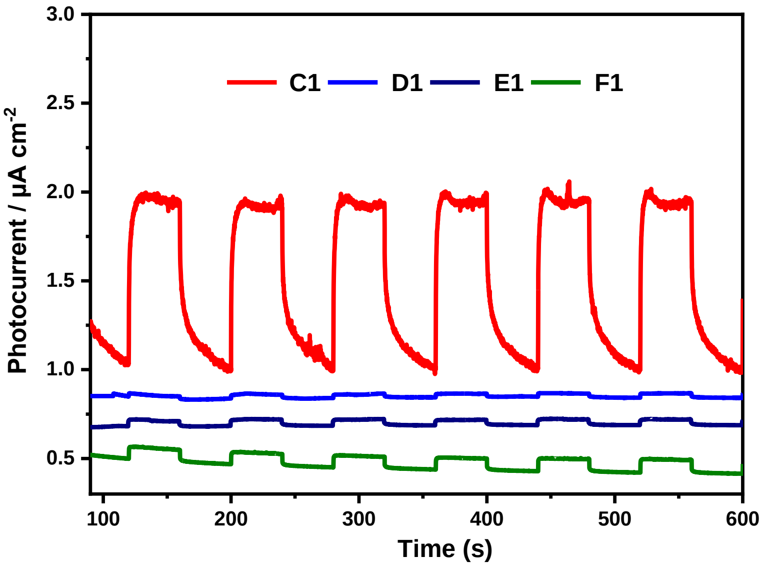


Figure S41. Photocurrent tests of the **C1 - F1** series compounds.

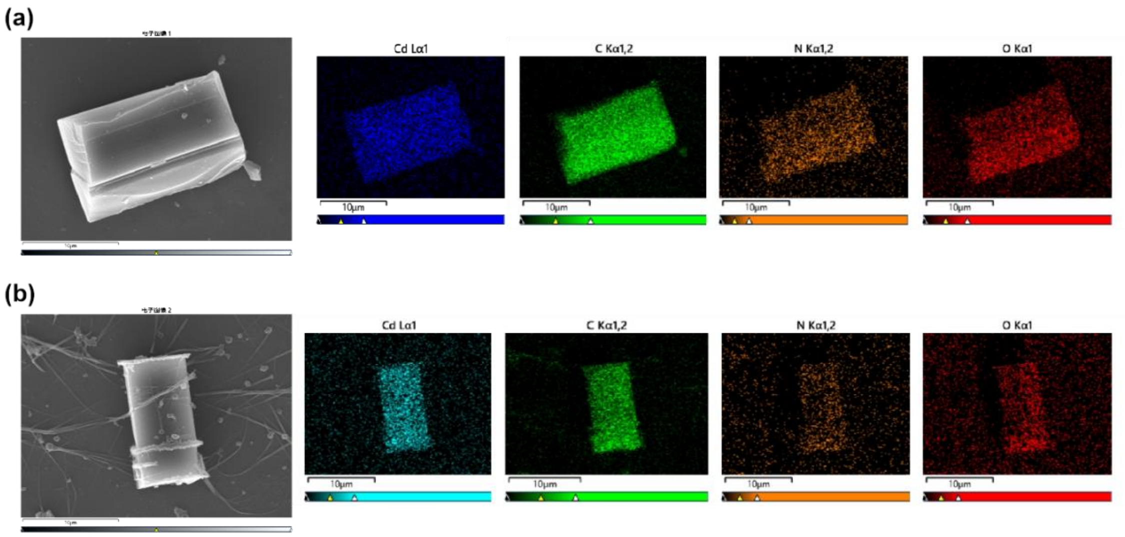


Figure S42. SEM images of **C1** obtained (a) before the CEES photooxidation reaction. (b) after the CEES photooxidation reaction.

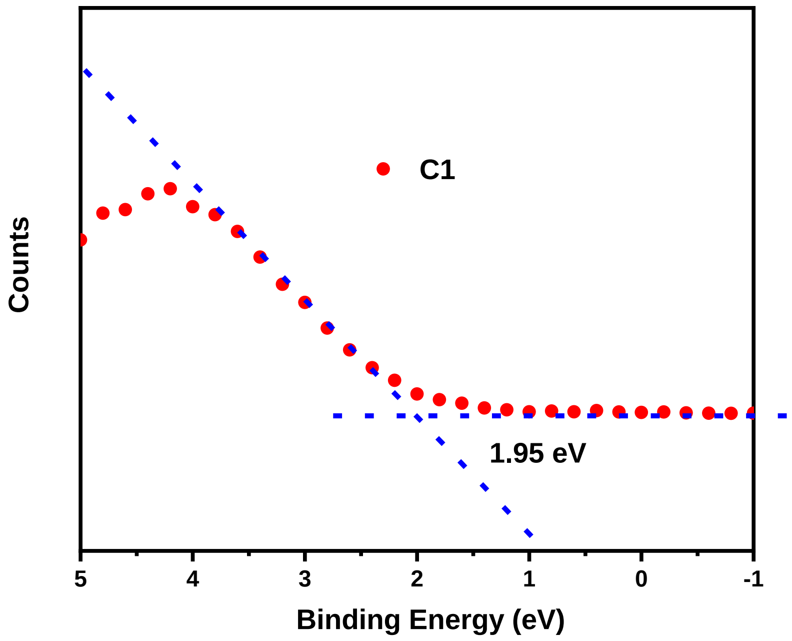


Figure S43. VB-XPS spectra of **C1**

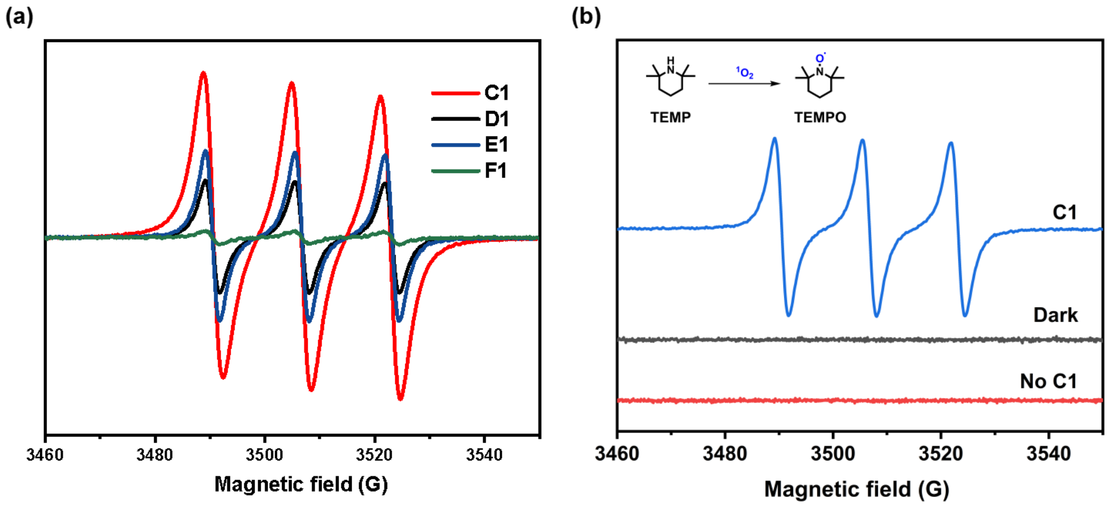


Figure S44. (a) EPR spectra of **C1**, **D1**, **E1**, and **F1** in the presence of TEMP under 425 nm visible-light irradiation for 10 min. (b) EPR spectra with TEMP as trapping agents for the confirmation of $^1O_2$ generation with **C1** under visible-light irradiation.

**EPR measurements:** EPR experiments were performed with a mixture of the 1.0 mg $mL^{-1}$ **C1** and 0.1 M TEMP or DMPO $O_2$-saturated deuterated methanol suspension in the dark or irradiated by 300 W xenon lamp (100 $mW/cm^2$).

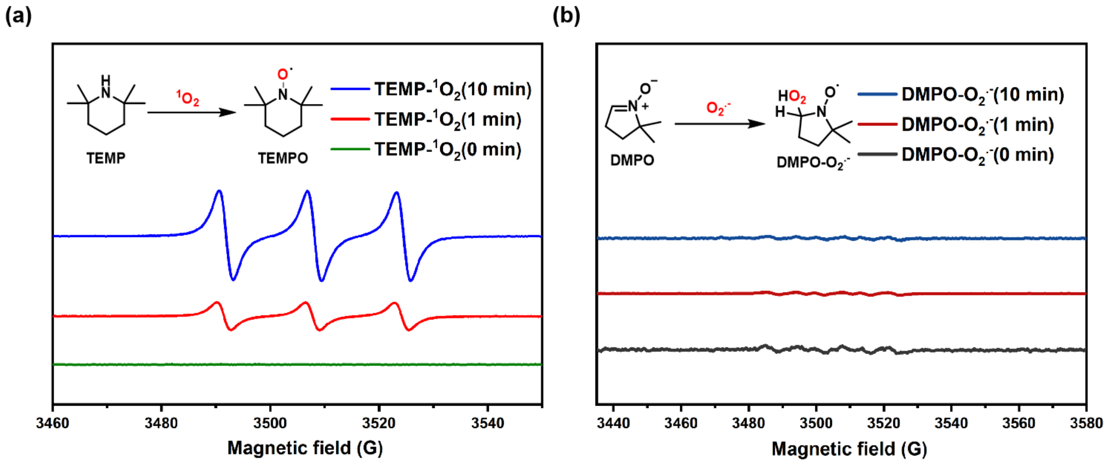


Figure S45. EPR spectra of **C1** in the presence of TEMP (a) and DMPO (b) under visible-light irradiation for 10 min.

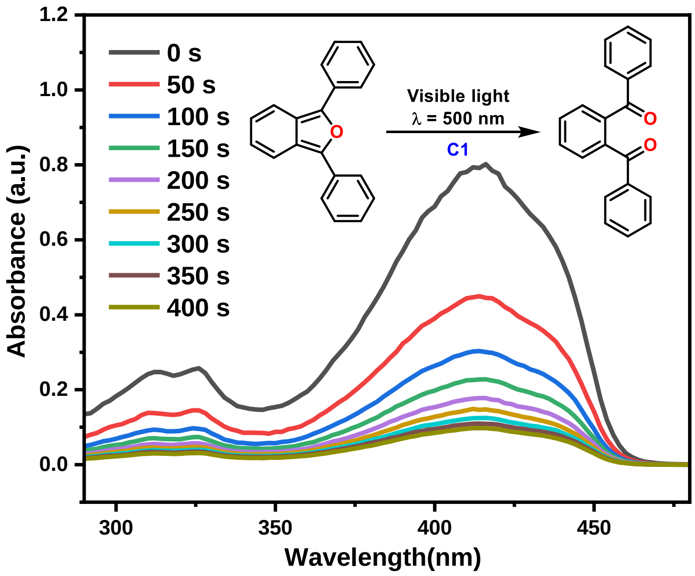


Figure S46. Time-dependent absorption spectra of DPBF in DMF upon visible light irradiation ($\lambda$ = 500 nm) in the presence of **C1**.

**Mustard Simulant Oxidation:**

As a standard procedure, **C1** (10 mg, 1 μmol) was dispersed in 1.0 mL of $CD_3OD$ in a sealed glass reactor. After purging with $O_2$ for 15 min, CEES (12.5 mg, 0.1 mmol) with 1.0 mL $CD_3OD$ was added to the glass reactor. After sealing the reactor, it was then exposed to 425 nm LED lamp (350 $mW/cm^2$) in $O_2$ at room temperature monitored by $^1H$ NMR measurement. Measurement under the atmospheric condition was conducted by purging with oxygen ($O_2$) at the beginning of the reaction.

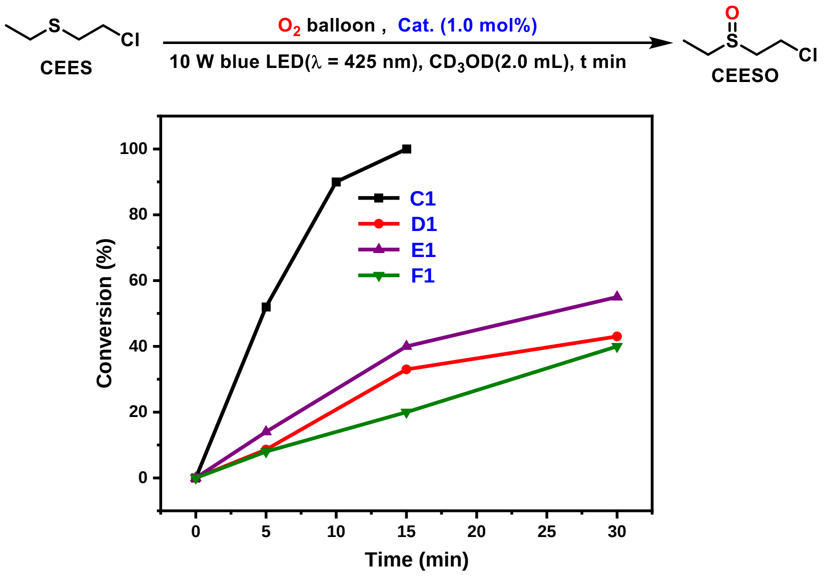


Figure S47. Photocatalytic conversion of thioanisole (CEES) under 1 atm of $O_2$ using 1 mol% Catalyst irradiated with blue LED (350 $mW{\bullet}cm^{-2}$) light in 2 mL of $CD_3OD$.

**Table S2**. Time-depended conversion of photo-oxidized CEES experiments by using **C1** as catalyst under the irradiation of 425 nm

| Catalyst | C1 | D1 | E1 | F1 |
|---|---|---|---|---|
| 0 s | 0 | 0 | 0 | 0 |
| 5 min | 50% | 8.6% | 14% | 8% |
| 10 min | 92% | 25% | 30% | 15% |
| 15 min | > 99% | 33% | 40% | 20% |
| 30 min | / | 43% | 55% | 30% |

**Table S3:** Control experiments of photocatalytic CEES oxidation with **C1**

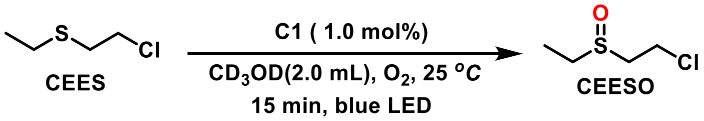


| entry[a] | charge from the standard conditions | Yield [%][b] |
|---|---|---|
| *1* | *none* | *> 99* |
| 2 | no C1 | trace |
| 3 | $N_2$ instead of $O_2$ | trace |
| 4 | Air instead of $O_2$ | 50 |
| 5 | no light | ND[d] |
| 6[c] | add DABCO | ND[d] |
| 7 | 5 min instead of 15 min | 50 |
| 8 | $CH_3OH$ instead of $CD_3OD$ | 95 |
| 9 | $CH_3CN$ instead of $CD_3OD$ | 97 |
| 10 | 50 °C instead of 25 °C | 90 |
| 11 | no solvent | 94 |
| 12 | red LED instead of blue LED | trace |
| 13 | green LED instead of blue LED | trace |
| 14 | D1 instead of C1 | 42 |
| 15 | E1 instead of C1 | 55 |
| 16 | F1 instead of C1 | 30 |

[a] Reaction Conditions: **C1** (1.0 mmol%), CEES (0.1 mmol), $CD_3OD$ (2.0 mL), r.t., 30 min, 10 W blue LED (λ = 425 nm), $O_2$ balloon. [b] $^1H$ NMR yield with $CH_2Br_2$ as the internal standard. [c] DABCO (0.5 mmol) as $^1O_2$ sacrificial agent.[d] No Detected.

**Table S4:** Control experiments of photocatalytic CEES oxidation without **C1**

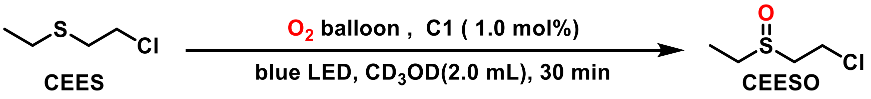


| Entry[a] | Deviation | Yield[b] (%) |
|---|---|---|
| *1* | *none* | *> 99* |
| 2 | tpp instead of C1 | ND[c] |
| 3 | Icz instead of C1 | ND[c] |

[a] Reaction Conditions: **C1** (1.0 mmol%), CEES (0.1 mmol) $CD_3OD$ (2.0 mL), r.t., 30 min, 10 W blue LED (λ = 425 nm), $O_2$ balloon. [b] $^1H$ NMR yield with $CH_2Br_2$ as the internal standard. [c] Not Detected.

**Table S5:** Solvent screening of the photocatalytic CEES oxidation with **C1**

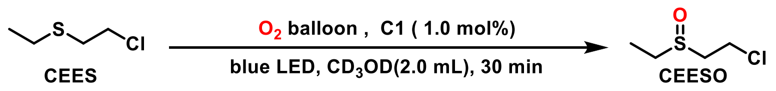


| Entry | Deviation | Yield[a] (%) |
|---|---|---|
| *1* | *none* | *> 99* |
| 2 | $CH_3OH$ instead of $CD_3OD$ | 95 |
| 3 | $CH_3CN$ instead of $CD_3OD$ | 97 |
| 4 | $H_2O$ instead of $CD_3OD$ | 80 |
| 5 | $CH_2Cl_2$ instead of $CD_3OD$ | 85 |

[a] Reactions were run on 0.1 mmol scale.Yields were determined by crude $^1$H NMR using $CH_2Br_2$ as internal standard.

**Table S6:** Temperature screening of the photocatalytic CEES oxidation with **B1**.

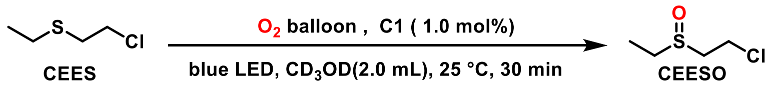


| Entry | Deviation | Yield[a] (%) |
|---|---|---|
| *1* | *none* | *> 99* |
| 2 | 5 °C instead of 25 °C | > 99 |
| 3 | 15 °C instead of 25 °C | > 99 |
| 4 | 35 °C instead of 25 °C | 95 |
| 5 | 50 °C instead of 25 °C | 90 |

[a] Reactions were run on 0.1 mmol scale.Yields were determined by crude $^1$H NMR using $CH_2Br_2$ as internal standard.

**Table S7.** Comparison of the CEES detoxification performance of various PCs

| catalyst | catalyst/ CEES ratio | LED (mW cm$^{-2}$) | T (min) | conv. (%) | sel. (%) | ref. |
|---|---|---|---|---|---|---|
| **PCN-57-S** | 0.1 | UV (865) | 25 | ＞99 | ＞99 | S2 |
| **PCN-57-Se** | 0.1 | purple (865) | 12 | ＞99 | ＞99 | S2 |
| **NU-1000** | 1 | UV (450) | 15 | ＞99 | ＞99 | S3 |
| **NU-1000-PCBA** | 1 | UV (450) | 7.1 | ＞99 | ＞99 | S3 |
| **PCN-222** | 0.5 | Blue (325) | 25 | ＞99 | ＞99 | S4 |
| **Br-BDP@NU-1000** | 0.2 | green (450) | ~5 | ＞99 | ＞99 | S5 |
| **Ag12TPyP** | 1 | White (80) | 4 | 98 | ＞99 | S6 |
| **UMCM-313** | 1 | UV (450) | >12 | ＞99 | ＞99 | S7 |
| **C1** | **1** | **Blue (350)** | **10-15** | **＞99** | **＞99** | **This work** |

## The calculation of apparent quantum yield

The AQY of **C1** was determined to be 12% at 425 nm (350 mW/cm$^{-2}$). The apparent quantum yield (AQY) of **C1** was determined using a similar procedure to that for the photocatalytic performance test: 0.1 mmol CEES, 10 mg **C1**, 2 mL $CD_3OD$, $O_2$ atmosphere, 25 ℃, 425 nm Blue LED. The intensity of the incident light was 350 W m$^{-2}$, the irradiated area was 0.785 ×10$^{-4}$ m$^2$, and the CEESO production rate of **C1** was 0.5 ×10$^{-3}$/15 ×60 mol s$^{-1}$ in 15 min. The AQY can be calculated as follows:

$$AQY\ (\%) = \frac{amounts\ of\ products\ formed}{amounts\ of\ photons\ irradiated} \times 100\%$$

$$= \frac{N_e}{N_p} = \frac{nN_A}{IS / h\nu t} = \frac{nhcN_A}{IS\lambda t} \times 100\%$$

in which, Ne represents the amounts of products formed; Np represents the amounts of photons irradiated; n is the molar amounts of CEESO formed ($0.1\times 10^{-3}$ mol); t is the reaction time ($15\times60$ s); NA is Avogadro constant ($6.022\times10^{23}$ $mol^{-1}$); I is the incident light intensity at certain wavelength ($350\times10$ W $m^{-2}$); S is the irradiated area ($0.785 \times 10^{-4}$ $m^2$); h is Planck constant ($6.626\times10^{-34}$ J s); c is the speed of light ($2.998\times10^8$ m $s^{-1}$); λ is the wavelength of incident light ($425\times10^{-9}$ m).

Under the similar condition as literature (*Angew. Chem. Int. Ed.* **2015**, *54*, 9001.), which is 0.5% **PCN-222** loading and blue LED irradiation, CEES can be completely oxidized to CEESO within 25 min. Therefore, we calculated AQY of **PCN-222** by the above formula in which t is $25\times60$ s; I is $350\times10$ W $m^{-2}$, n is $0.1\times 10^{-3}$ mol, S is $0.785 \times 10^{-4}$ $m^2$; λ is $425\times10^{-9}$ m. The AQY of **PCN-222** was determined to be 7.2% for the CEES oxidation reaction, which is lower than **C1**, indicates **C1** possesses higher efficiency in CEES oxidation than **PCN-222**.

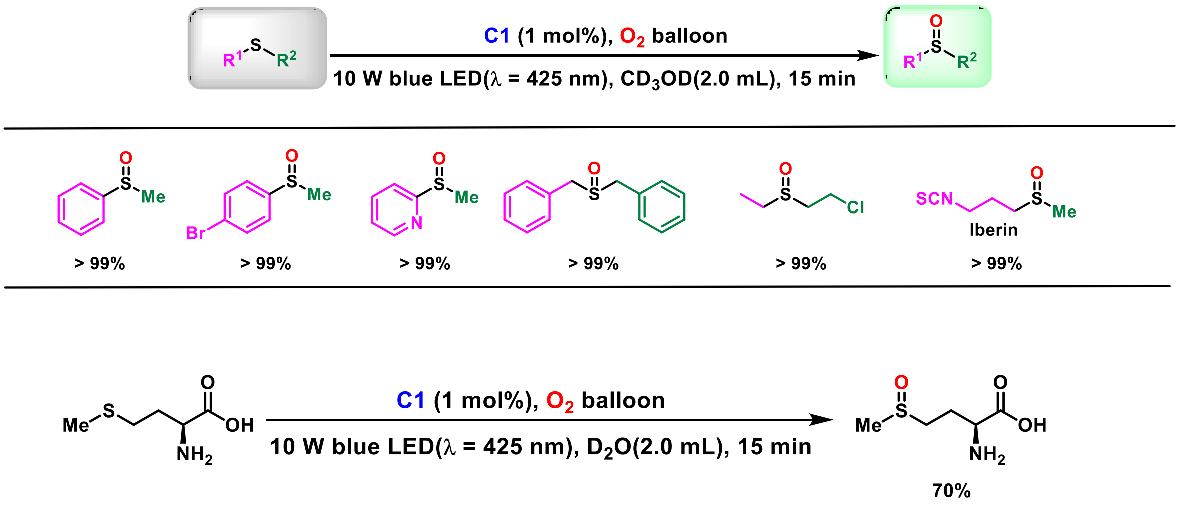


Figure S48. Scope of mustard-gas simulant oxidation and the extending of substrates.

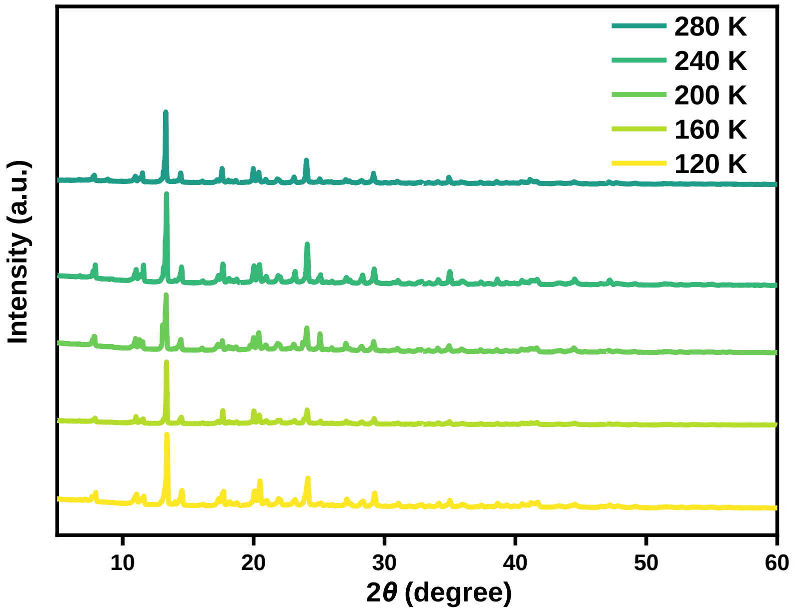


Figure S49. Powder X-ray diffraction (PXRD) of **C1** from 120 K to 280 K

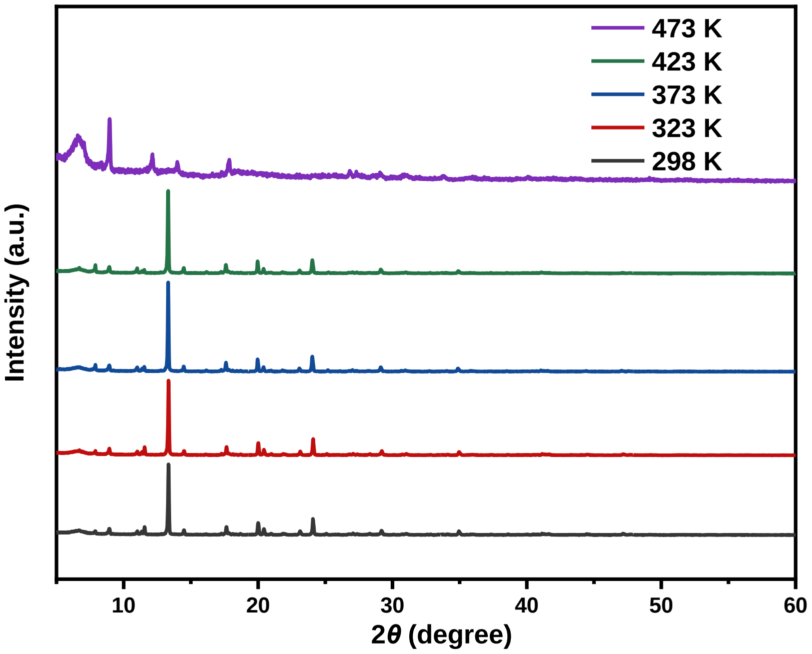


Figure S50. Variable temperature powder X-ray diffraction (PXRD) of **C1**

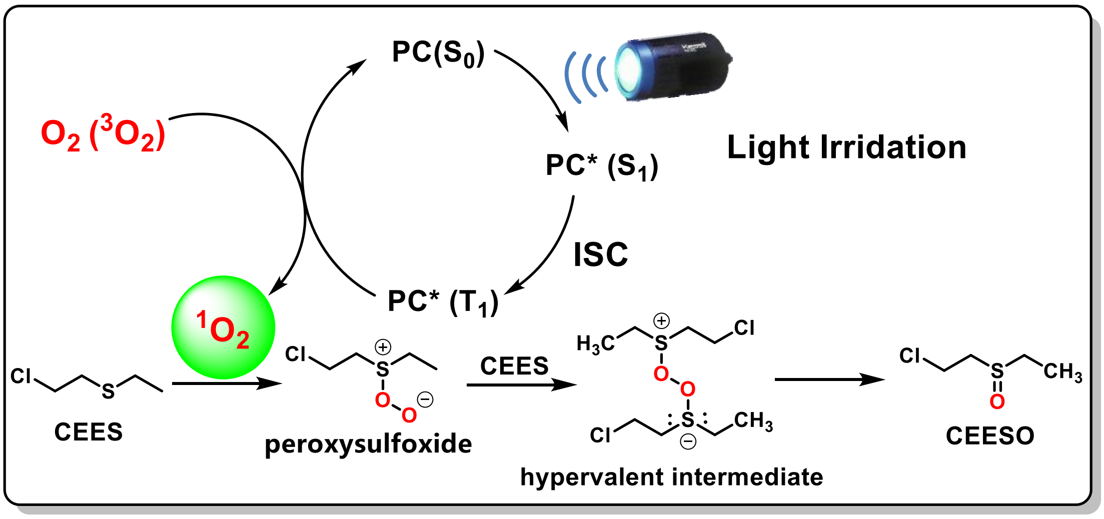


Figure S51. The oxidation mechanism of CEES by singlet oxygen($^1O_2$).

**Proposed Mechanism:** On the basis of above results, here we propose plausible reaction mechanism for CEES oxidations with **C1** as photocatalyst (Figure S60). Initially, electrons in the $S_0$ are excited into the $S_1$ upon visible light irradiation, generating free electrons and positively charged holes. Due to the nearly identical excited energy level of $S_1$ and $T_1$, the photo-induced electron could readily transfer to $T_1$ through ISC process. Then the EnT processes could readily occur between the triplet excited electron and the ground state $^3O_2$ to generate $^1O_2$, based on the charge separation and transport features of **C1**. Then the $^1O_2$ reacts with CEES to produce a diradical peroxysulfoxide intermediate, which reacts with another CEES molecule to produce the hypervalent intermediate, and then afford sulfoxide product CEESO.

To probe the reaction mechanisms, several control experiments have been conducted (Fig. S47 in the revised supplementary data). Specifically, the addition of the scavengers for holes (KI), electrons ($CuSO_4$) and $O_2^{\bullet-}$ (p-benzoquinone, p-BQ) slightly affect the yields of reactions. However, the addition of $^1O_2$ quencher (β-carotene) significantly reduce the yield of the CEESO product. The results indicate that $^1O_2$ via energy transfer process may play a more important role in oxidation.

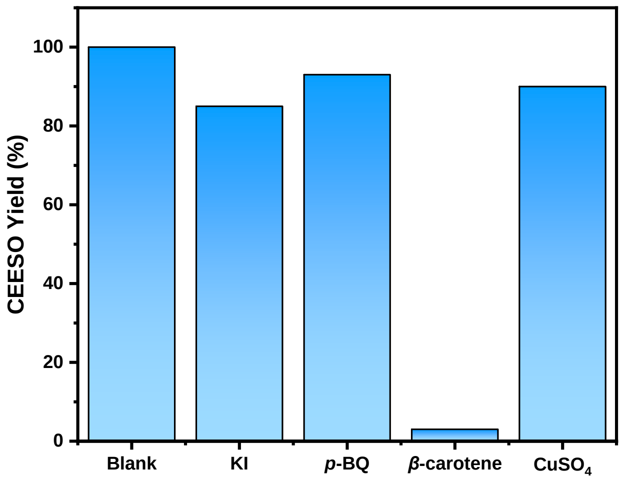


Figure S52. Effects of scavengers on the photocatalytic oxidation of dihydroartemisinic acid to artemisinin of **C1** under standard reaction conditions.

**Sequential Photochemical Synthesis of Artemisinin:** The synthesis of artemisinin was conducted using **C1** as photocatalyst under a 425 nm LED lamp (350 mW/cm$^2$) in the presence of $O_2$. In a typical reaction, dihydroartemisinic acid (25 mg, 0.105 mmol), Catalyst **C1** (10 mg, 1 μmol) was dispersed in $CD_3OD$ (2.0 mL) and slowly bubbled with $O_2$ under the irradiation of blue LED lamps at 25 ℃ for 3 hours. The resulting heterogeneous mixture was then centrifugated and filtered, washed with ethanol for recycling catalyst. Trifluoroacetic acid (8 μL) were added in filtrate and slowly bubbled with $O_2$ for 3 hours. Afterwards, the solid was filtered off and organic solution was concentrated. Pure artemisinin was isolated from the crude reaction mixture by flash column chromatography (silica, hexane/EtOAC, 5-20% gradient) as a white solid. Conversion and selectivity to artemisinin was measured by $^1$H NMR in $CD_3OD$ using biphenyl as an internal standard.

**Table S8.** Comparison of the ability for photochemical synthesis of artemisinin by using various photosensitizers with $^1O_2$

| catalyst | catalyst ratio % | LED (mW cm$^{-2}$) | T(h) | conv. (%) | sel. (%) | ref. |
|---|---|---|---|---|---|---|
| **PCN-134(Ni)-2D** | 2 | Blue (150) | 3 | 97 | 64 | S8 |
| **PCN-222(Ni)-0.005M** | 2 | Blue (150) | 5 | 98 | 71 | S9 |
| **TPFPP** | 0.1 | White (450) | 5 | 92 | 50 | S10 |
| **$[Ru(bpy)_3]Cl_2$** | 0.1 | White (450) | 6 | ＞98 | 87 | S10 |
| **PCN-808-BDBR** | 2 | Blue (150) | 3 | 92 | 51 | S11 |
| **MB/Yb-TCPP-$HSO_4$-0.005M** | 1 | Xe lamp (300 W) | 3 | 98 | 85 | S12 |
| **C1** | **1** | **Blue (350)** | **3** | **＞99** | **88** | **This work** |

**Table S9.** Control experiments of photocatalytic dihydroartemisinic acid oxidation with **C1**.

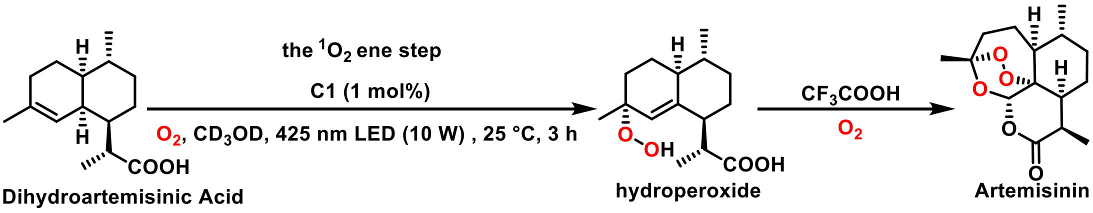


| entry[a] | charge from the standard conditions | Conversion[%][b] | Selectivity of the $^1O_2$ ene step[%][b] |
|---|---|---|---|
| 1 | *none* | > 99 | 88 |
| 2 | no C1 | trace | / |
| 3 | $N_2$ instead of $O_2$ | 0 | / |
| 4 | no light | 0 | / |
| 5 | 455 nm LED instead of 425 nm LED | 18 | 80 |
| 6 | 1 h instead of 3 h | 50 | 85 |
| 7 | $CDCl_3$ instead of $CD_3OD$ | 40 | 85 |
| 8 | 5 °C instead of 25 °C | 68 | 91 |
| 9 | 45 °C instead of 25 °C | 80 | 85 |

[a] Reactions were run on 0.1 mmol scale.[b] Conversion and selectivity were determined by crude $^1H$ NMR using biphenyl as internal standard.

**Table S10.** Solvent screening of the photocatalytic dihydroartemisinic acid oxidation with **C1**.

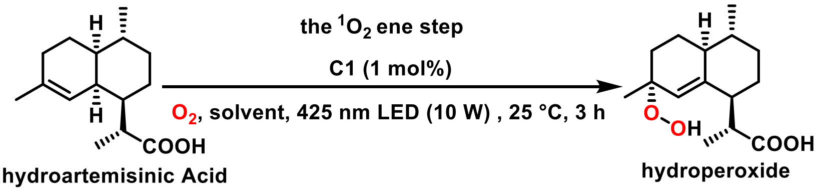


| entry[a] | solvent | Conversion[%][b] | Selectivity of the $^1O_2$ ene step[%][b] |
|---|---|---|---|
| 1 | *$CD_3OD$* | > 99 | 88 |
| 2 | $CDCl_3$ | 40 | 88 |
| 3 | $CH_3CN$ | 90 | 88 |
| 4 | $CH_3OH$ | 78 | 85 |
| 5 | $CH_3CH_2OH$ | 85 | 85 |
| 6 | $CH_2Cl_2$ | 75 | 85 |
| 7 | EA | 33 | 80 |
| 8 | THF | 40 | 85 |
| 9[c] | $CH_3CN$ | > 99 | 88 |
| 10[d] | $CH_3OH$ | > 95 | 88 |

[a] Reactions were run on 0.1 mmol scale. [b] Conversion and Selectivity were determined by crude $^1H$ NMR using biphenyl as internal standard. [c] 5 h instead of 3 h. [d] 8 h instead of 3 h.

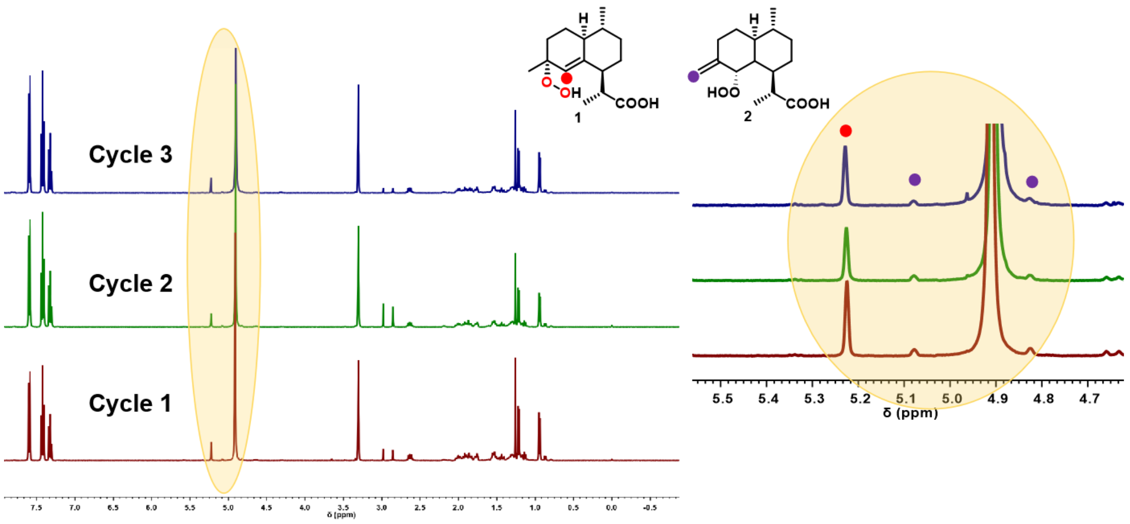


Figure S53. Reusability of **C1** over three consecutive cycles of dihydroartemisinic acid photooxidation. The reaction progress was monitored by $^{1}$H NMR under Blue LED at 25 ℃ with three recycling experiments. The catalytic activity for 3 runs.

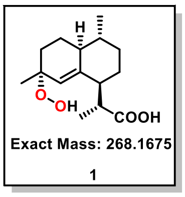


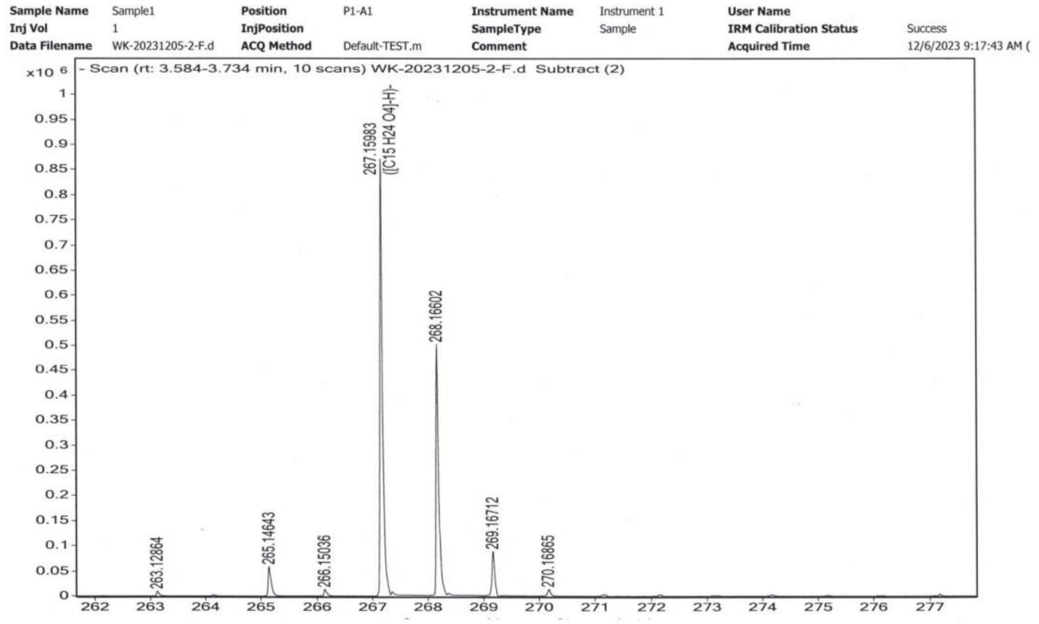


Figure S54. HRMS spectra of Intermediate **1**.

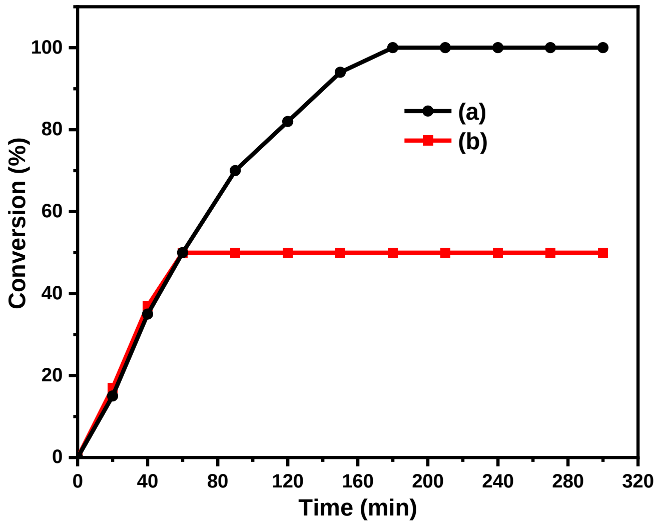


Figure S55. Leaching test for the photocatalytic oxidation of dihydroartemisinic acid to artemisinin over **C1** under optimized reaction conditions. After 60 min of the reaction, the catalyst was filtered out whereas the filtrate was further reacted under identical conditions: (a) the common catalytic process and (b) hot filtration test.

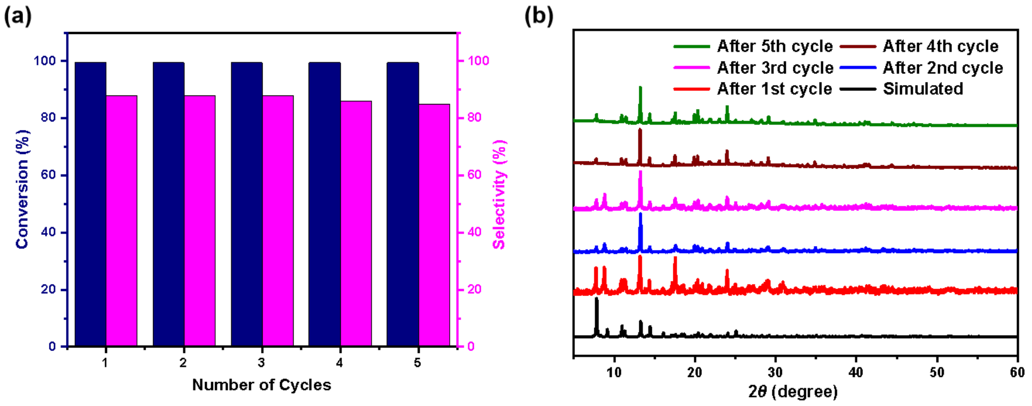


Figure S56. (a) The recycling experiments with **C1** as photocatalyst for the photocatalytic oxidation of dihydroartemisinic acid to artemisinin. (b) PXRD of **C1** before and after five recycling experiments in the $^{1}O_2$ ene step.

**Table S11**. Investigation of the photocatalytic oxidation of dihydroartemisinic acid to artemisinin using **C1** as the catalyst.

| Cycles | Conversion (%) | Selectivity (%) |
|---|---|---|
| 1 | ＞ 99 | 88 |
| 2 | ＞ 99 | 88 |
| 3 | ＞ 99 | 88 |
| 4 | ＞ 99 | 86 |
| 5 | ＞ 99 | 85 |

## 4) NMR Spectra

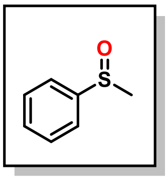

Yellow oil. $^{1}$H-NMR (400 MHz, $d^{4}$-MeOD) δ 7.73 -7.58 (m, 5H), 2.79 (s, 3H). $^{13}$C NMR (100 MHz, $d^{4}$-MeOD) δ 146.27, 132.64, 130.71, 124.93, 43.68. The spectroscopic data match a literature report.[13]

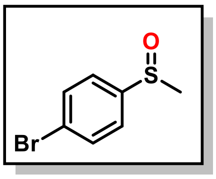

White solid; $^{1}$H-NMR (400 MHz, $d^{4}$-MeOD) δ 7.82 - 7.72 (m, 2H), 7.68 - 7.56 (m, 2H), 2.80 (s, 3H). $^{13}$C NMR (100 MHz, $d^{4}$-MeOD) δ 145.51, 133.79, 126.74, 126.67, 43.53. The spectroscopic data match a literature report.[14]

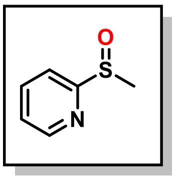

Yellow oil; $^{1}$H-NMR (400 MHz, $d^{4}$-MeOD) δ 8.79-8.54 (m, 1H), 8.10 (td, J = 7.7, 1.7 Hz, 1H), 7.96 (dt, J = 7.9, 1.1 Hz, 1H), 7.54 (ddd, J = 7.5, 4.8, 1.1 Hz, 1H), 2.88 (s, 3H).$^{13}$C NMR (100 MHz, $d^{4}$-MeOD) δ 166.01, 151.16, 140.01, 126.59, 120.45, 41.26. The spectroscopic data match a literature report.[14]

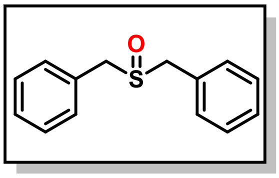

White solid; $^{1}$H-NMR (400 MHz, $d^{4}$-MeOD) δ 7.41-7.33 (m, 10H), 4.18 (d, J = 13.0 Hz, 2H), 3.96 (d, J = 13.0 Hz, 2H). $^{13}$C NMR (100 MHz, $d^{4}$-MeOD) δ 132.01, 131.48, 129.92, 129.45, 58.27. The spectroscopic data match a literature report.[13]

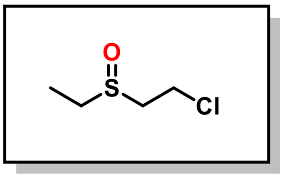

Colorless oil; $^{1}$H-NMR (400 MHz, $d^{4}$-MeOD) δ 4.09-3.82 (m, 2H), 3.27-3.07 (m, 2H), 3.01-2.71 (m, 2H), 1.35 (t, J = 7.5 Hz, 3H). $^{13}$C NMR (100 MHz, $d^{4}$-MeOD) δ 54.88, 46.45, 38.05, 7.05. The spectroscopic data match a literature report.[15]

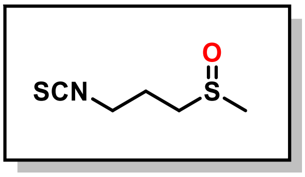


Colorless oil; $^{1}$H-NMR (400 MHz, d$^{4}$-MeOD) δ 3.78-3.52 (m, 2H), 2.97-2.73 (m, 2H), 2.65 (s, 3H), 2.03 (p, J = 7.2 Hz, 2H). $^{13}$C NMR (100 MHz, d$^{4}$-MeOD) δ 52.22, 43.71, 38.22, 23.86. The spectroscopic data match a literature report.[16]

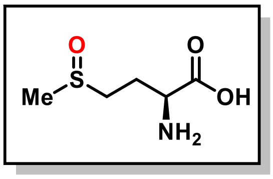


Yellow solid; $^{1}$H-NMR (400 MHz, $D_2O$) δ 3.87 (t, J = 6.4 Hz, 1H), 3.09-2.92 (m, 2H), 2.73 (s, 3H), 2.30 (q, J = 7.5 Hz, 2H).$^{13}$C NMR (100 MHz, $D_2O$) δ 173.18, 53.31, 48.24, 36.52, 23.74. The spectroscopic data match a literature report.[17]

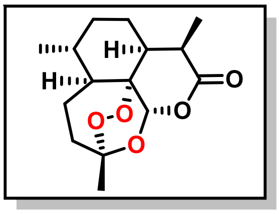


$^{1}$H NMR (400 MHz, $CDCl_3$) δ 5.85 (s, 1H), 3.38 (dd, *J* = 7.3, 5.4 Hz, 1H), 2.53-2.31 (m, 1H), 2.12-1.93 (m, 2H), 1.92-1.82 (m, 1H), 1.76 (ddt, *J* = 13.2, 9.8, 4.4 Hz, 2H), 1.52-1.32 (m, 6H), 1.19 (d, *J* = 7.3 Hz, 3H), 1.06 (dddd, *J* = 11.4, 9.1, 6.5, 2.1 Hz, 2H), 0.99 (d, *J* = 5.8 Hz, 3H).$^{13}$C NMR (100 MHz, $CDCl_3$) δ 172.03, 105.31, 93.64, 79.43, 49.96, 44.86, 37.45, 35.81, 33.51, 32.82, 25.13, 24.77, 23.32, 19.77, 12.50. The spectroscopic data match a literature report.[18]

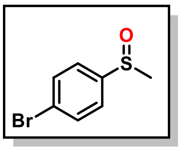
O
S
Br

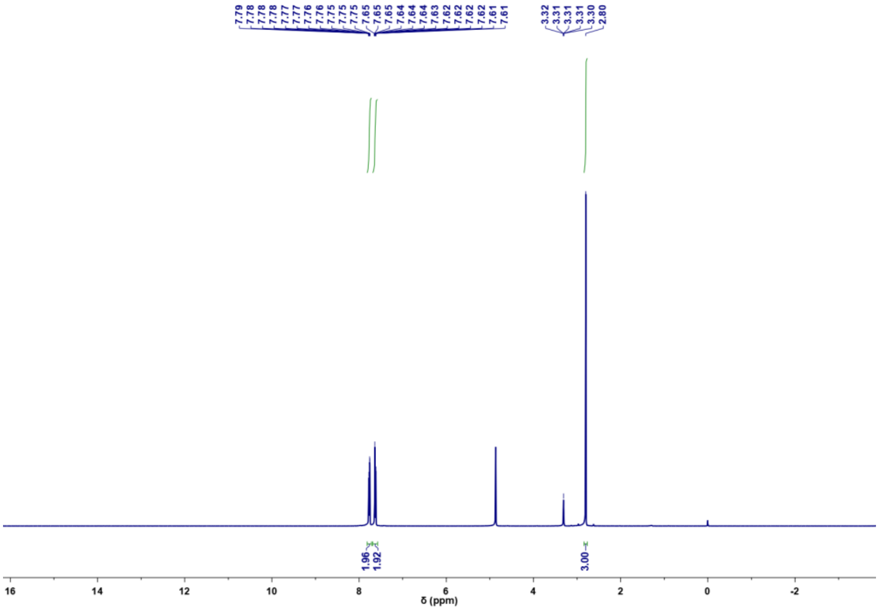
7.79
7.78
7.78
7.77
7.77
7.76
7.76
7.75
7.75
7.75
7.65
7.65
7.65
7.64
7.64
7.64
7.63
7.62
7.62
7.62
7.62
7.61
7.61
3.32
3.31
3.31
3.31
3.30
2.80
1.96
1.92
3.00
δ (ppm)

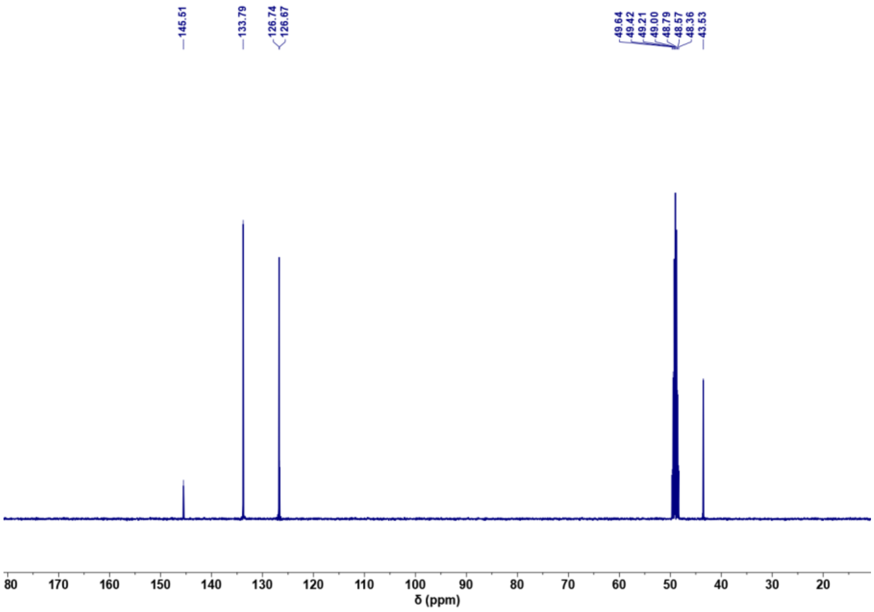
145.51
133.79
126.74
126.67
49.64
49.42
49.21
49.00
48.79
48.57
48.36
43.53
δ (ppm)

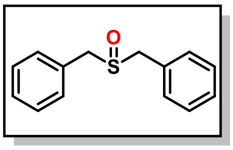
O
S

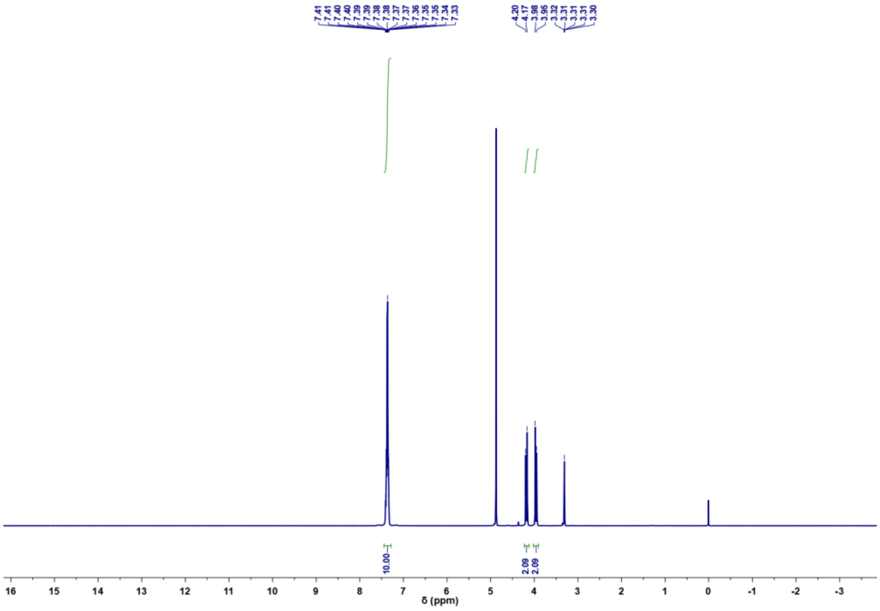
7.41
7.41
7.40
7.39
7.39
7.38
7.38
7.37
7.37
7.36
7.35
7.35
7.34
7.33
4.20
4.17
3.98
3.95
3.32
3.31
3.31
3.31
3.30
10.00
2.09
2.09
δ (ppm)

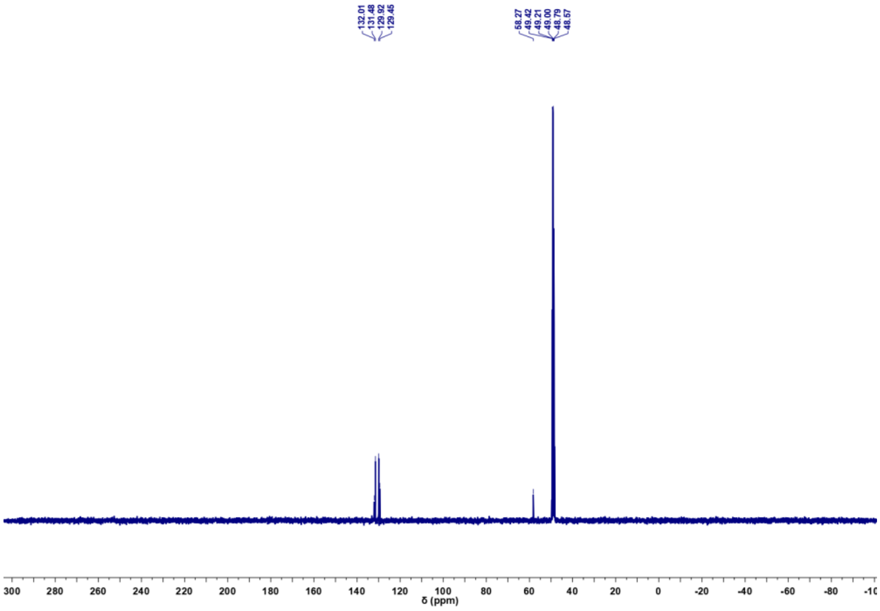
132.01
131.48
129.92
129.45
58.27
49.42
49.21
49.00
48.79
48.57
δ (ppm)

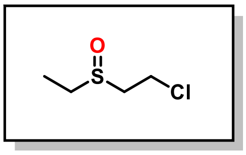
O
S
Cl

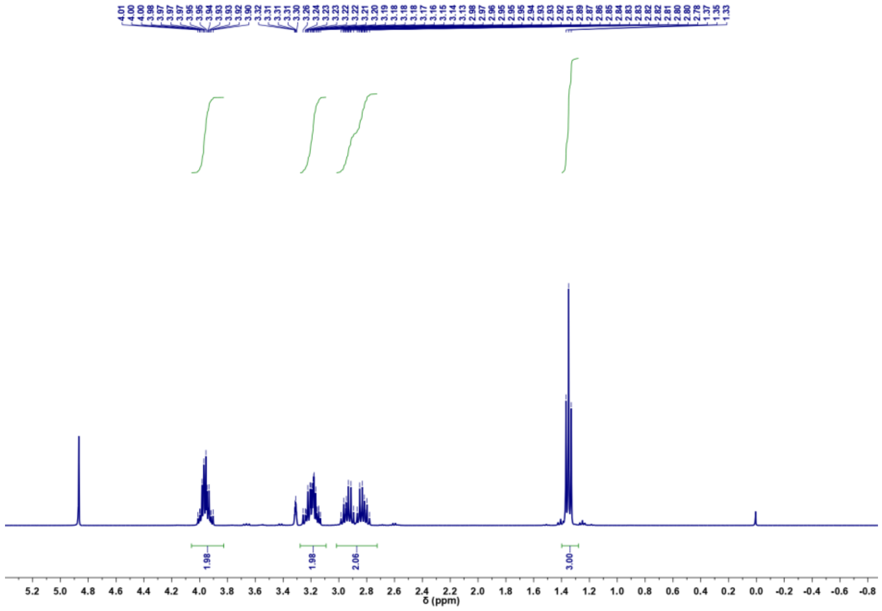
1.98
1.98
2.06
3.00
5.2 5.0 4.8 4.6 4.4 4.2 4.0 3.8 3.6 3.4 3.2 3.0 2.8 2.6 2.4 2.2 2.0 1.8 1.6 1.4 1.2 1.0 0.8 0.6 0.4 0.2 0.0 -0.2 -0.4 -0.6 -0.8
δ (ppm)

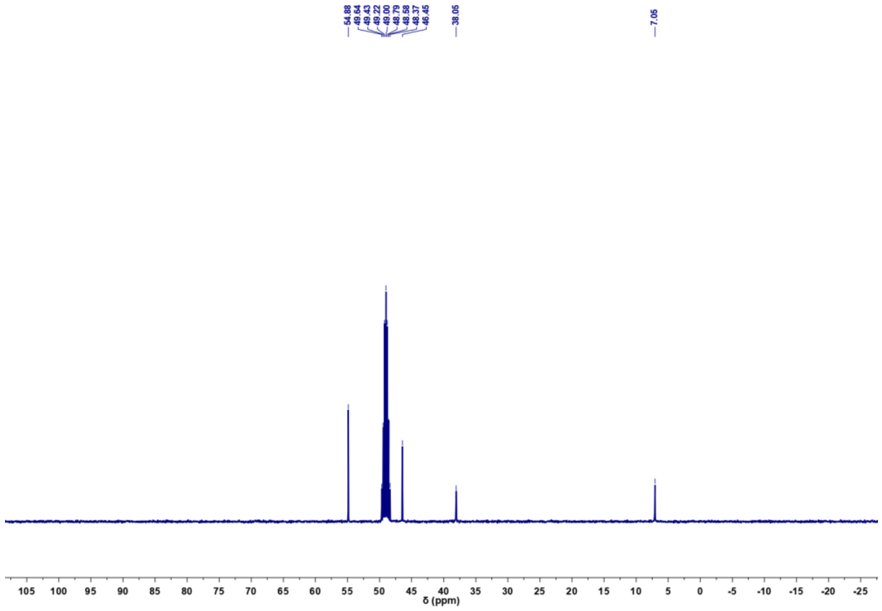
54.88
49.64
49.43
49.22
49.00
48.79
48.58
48.37
46.45
38.05
7.05
105 100 95 90 85 80 75 70 65 60 55 50 45 40 35 30 25 20 15 10 5 0 -5 -10 -15 -20 -25
δ (ppm)

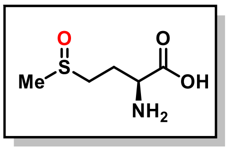

Me
S
O
O
OH
NH2


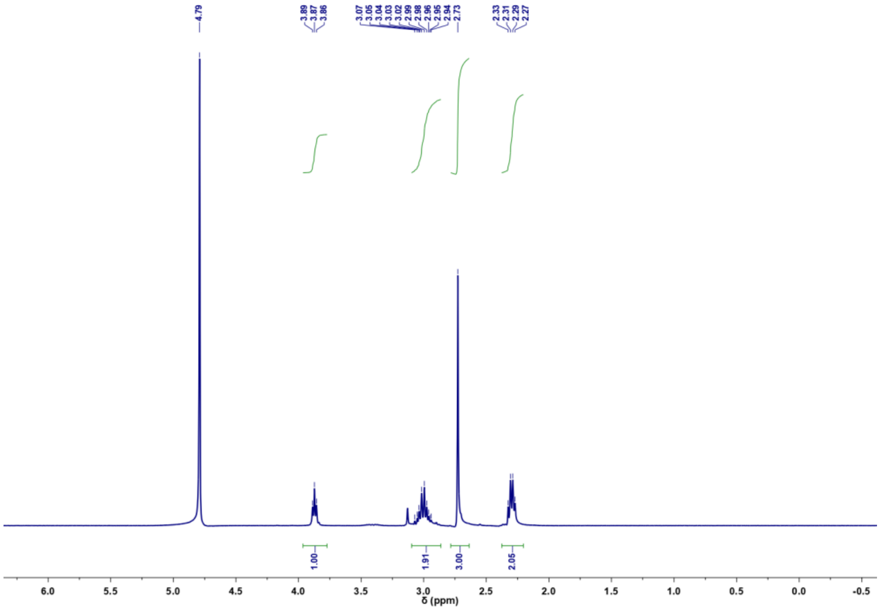

4.79
3.89
3.87
3.86
3.07
3.05
3.04
3.03
3.02
2.99
2.98
2.96
2.95
2.94
2.73
2.33
2.31
2.29
2.27
1.00
1.91
3.00
2.05
δ (ppm)


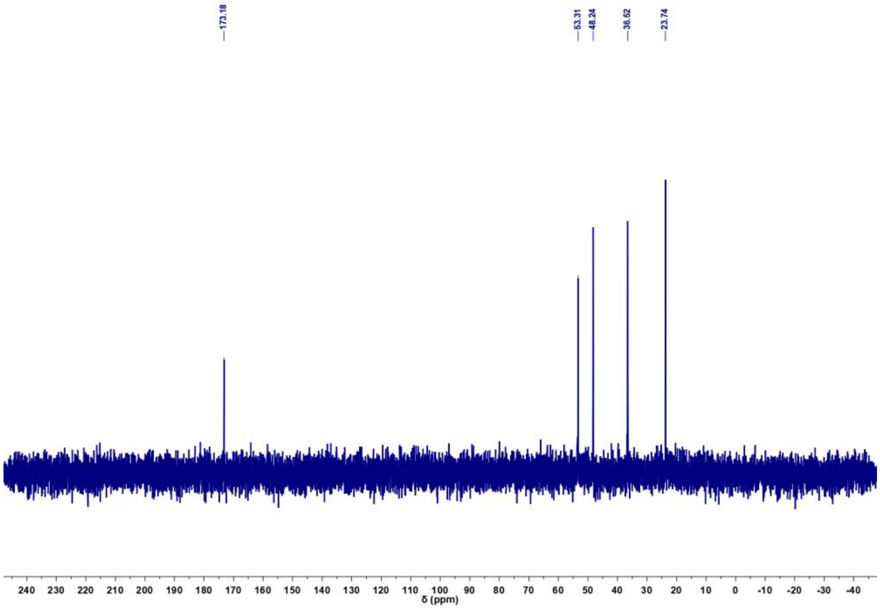

173.18
53.31
48.24
36.52
23.74
δ (ppm)

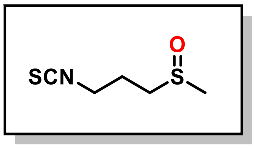
SCN
O
S

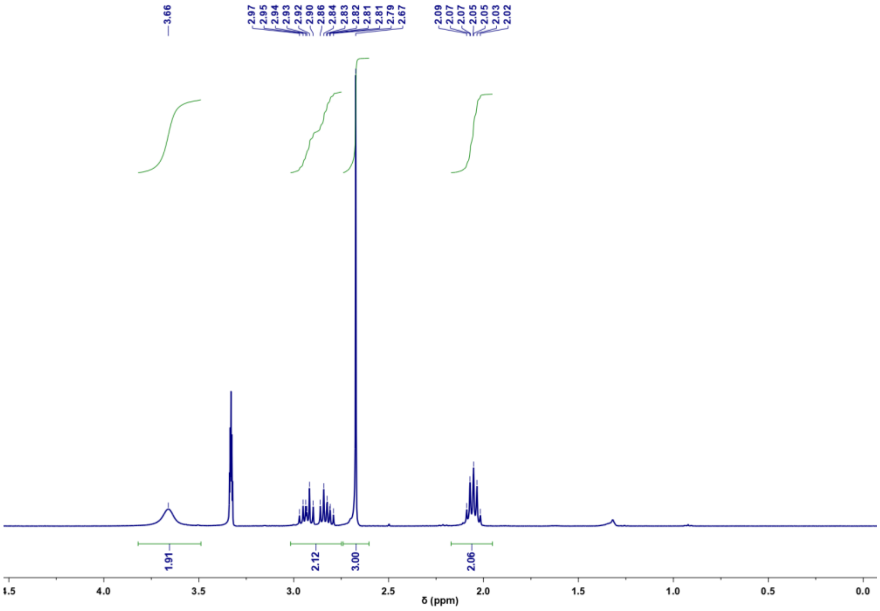
3.66
2.97 2.95 2.94 2.93 2.92 2.90 2.86 2.84 2.83 2.82 2.81 2.81 2.79 2.67
2.09 2.07 2.07 2.05 2.05 2.03 2.02
1.91
2.12
3.00
2.06
δ (ppm)

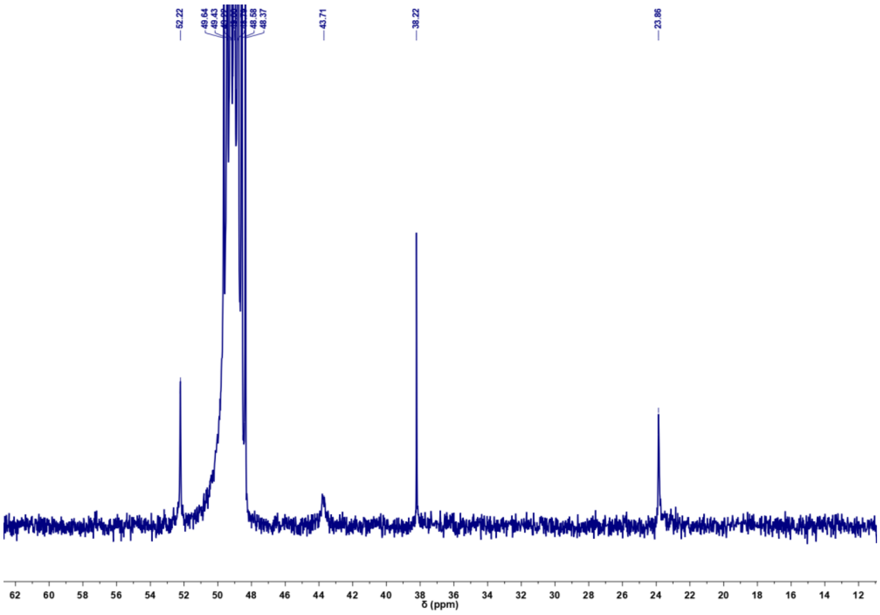
52.22
49.64 49.43
48.58 48.37
43.71
38.22
23.86
δ (ppm)

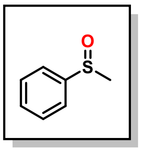
O
S

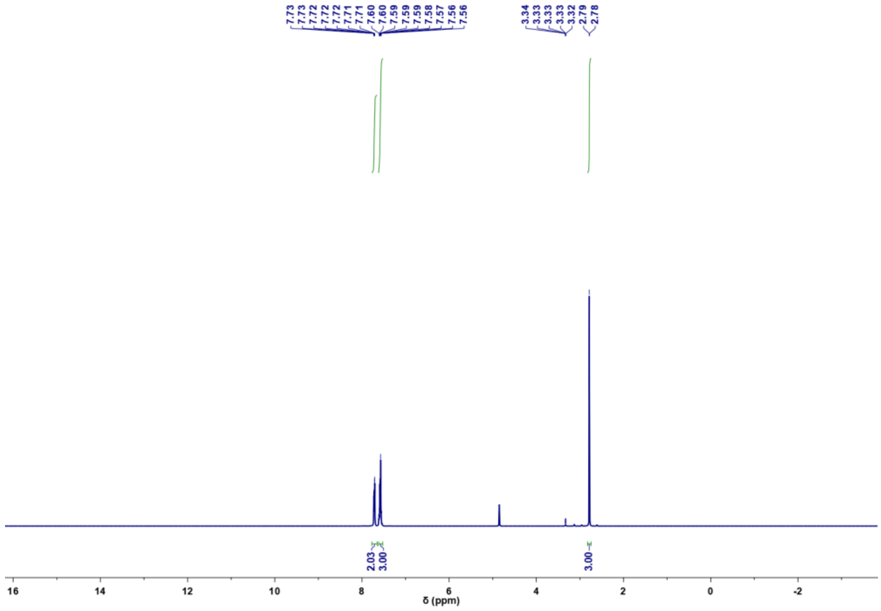
7.73
7.73
7.72
7.72
7.72
7.71
7.71
7.60
7.60
7.59
7.59
7.59
7.58
7.57
7.56
7.56
3.34
3.33
3.33
3.33
3.32
2.79
2.78
2.03
3.00
3.00
δ (ppm)

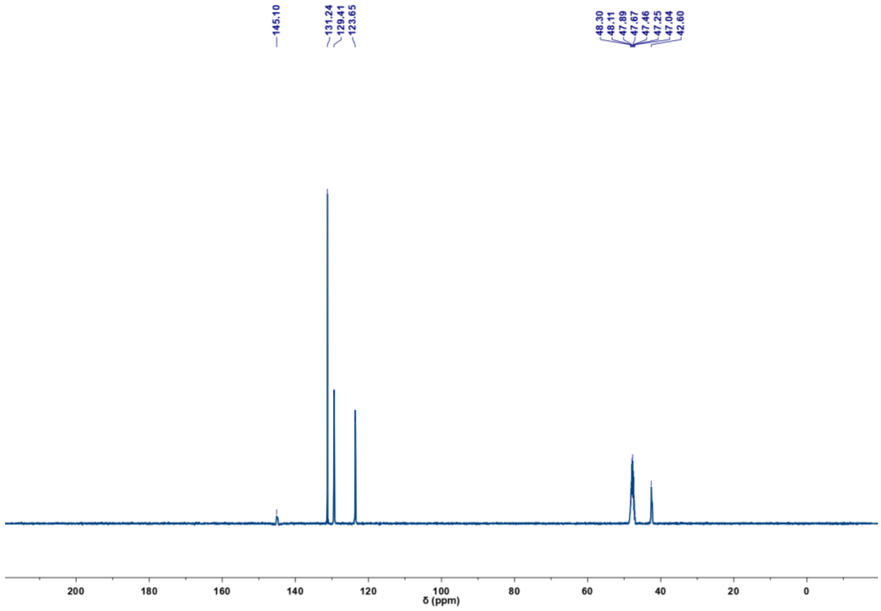
145.10
131.24
129.41
123.65
48.30
48.11
47.89
47.67
47.46
47.25
47.04
42.60
δ (ppm)

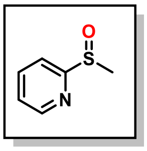

O
S
N


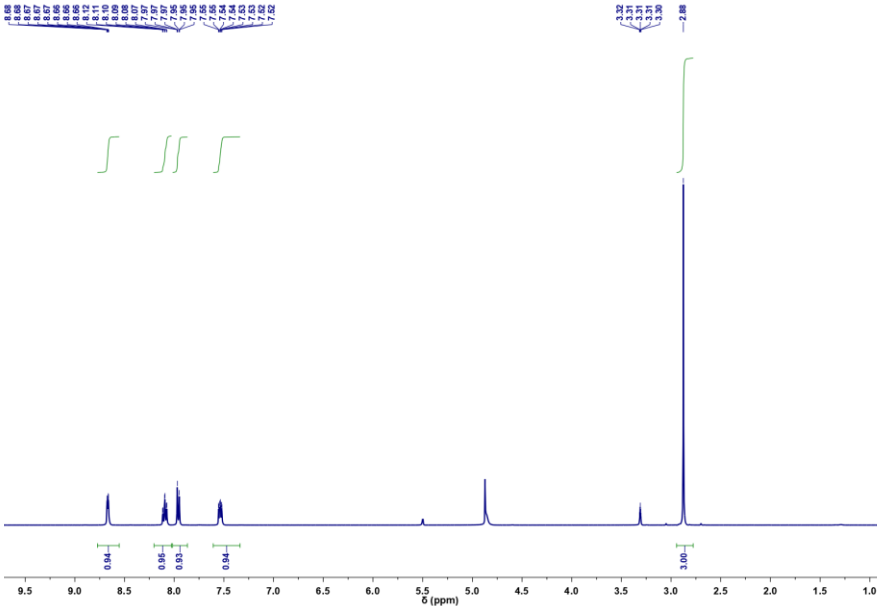

8.68 8.68 8.67 8.67 8.67 8.66 8.66 8.66 8.12 8.11 8.10 8.09 8.08 8.07 7.97 7.97 7.97 7.95 7.95 7.95 7.55 7.55 7.54 7.54 7.53 7.53 7.52 7.52
3.32 3.31 3.31 3.31 3.30
2.88
0.94
0.95
0.93
0.94
3.00
9.5 9.0 8.5 8.0 7.5 7.0 6.5 6.0 5.5 5.0 4.5 4.0 3.5 3.0 2.5 2.0 1.5 1.0
δ (ppm)


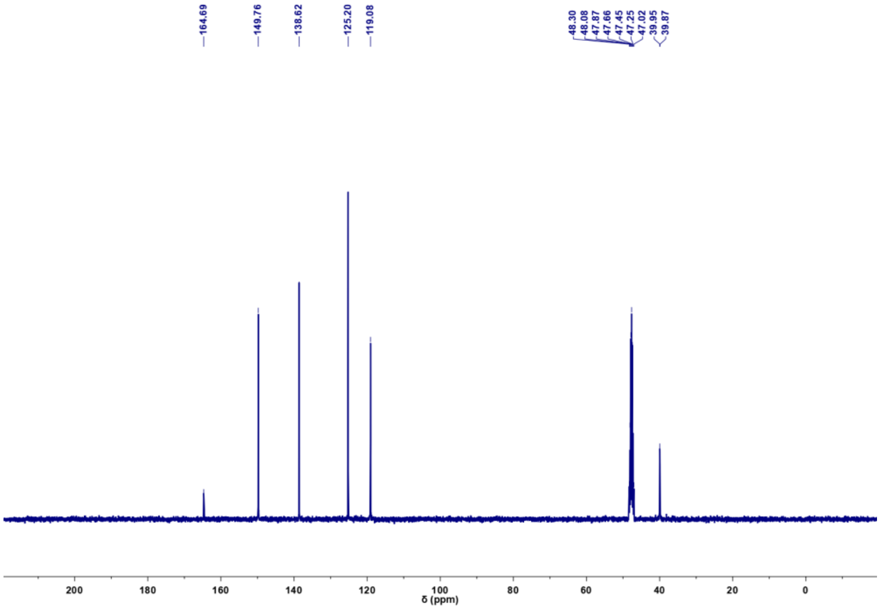

164.69
149.76
138.62
125.20
119.08
48.30 48.08 47.87 47.66 47.45 47.25 47.02
39.95 39.87
200 180 160 140 120 100 80 60 40 20 0
δ (ppm)

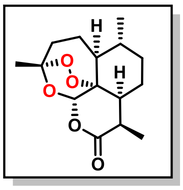

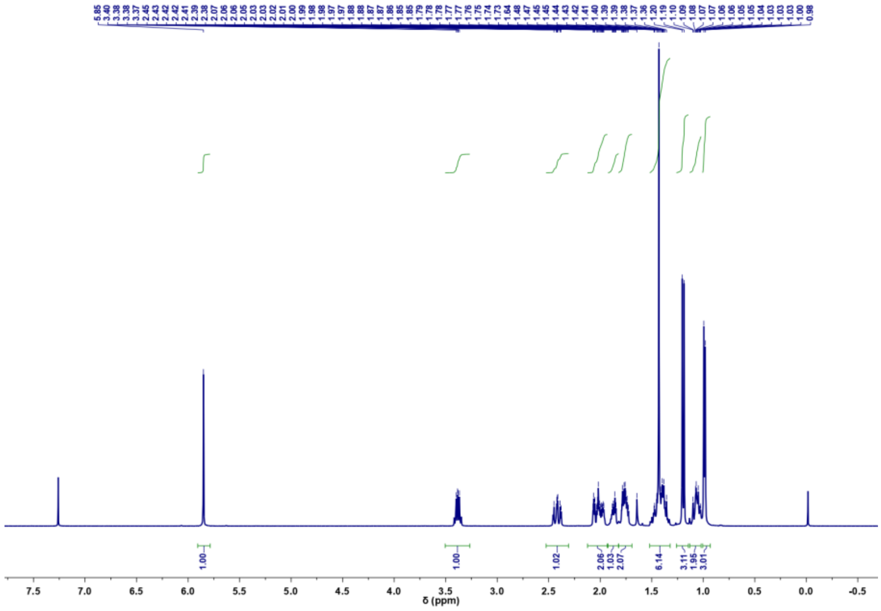

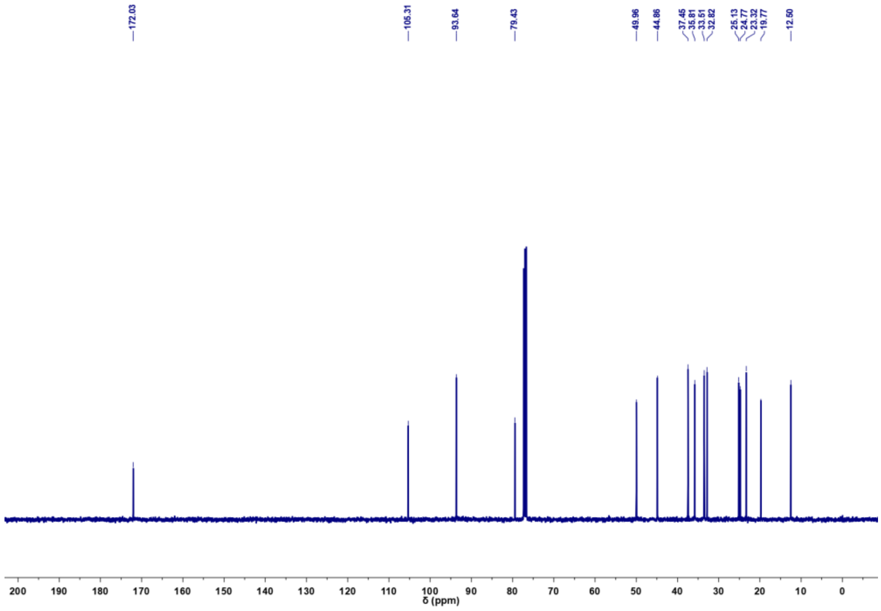

## 5) References


[1] Constable, E. C.; Zhang, G.; Housecroft, C. E.; Zampese, J. A. *CrystEngComm* **2011,** *13*, 6864-6870.

[2] Goswami, S.; Miller, C. E.; Logsdon, J. L.; Buru, C. T.; Wu, Y. L.; Bowman, D. N.; Islamoglu, T.; Asiri, A. M.; Cramer, C. J.; Wasielewski, M. R.; Hupp, J. T.; Farha, O. K. *ACS Appl. Mater. Interfaces* **2017**, *9*, 19535.

[3] Howarth, A. J.; Buru, C. T.; Liu, Y.; Ploskonka, A. M.; Hartlieb, K. J.; McEntee, M.; Mahle, J. J.; Buchanan, J. H.; Durke, E. M.; Al-Juaid, S. S.; Stoddart, J. F.; DeCoste, J. B.; Hupp, J. T.; Farha, O. K. *Chem.-Eur. J.* **2017**, *23*, 214.

[4] Liu, Y.; Howarth, A. J.; Hupp, J. T.; Farha, O. K. *Angew. Chem. Int. Ed.* **2015**, *54*, 9001.

[5] Atilgan, A.; Islamoglu, T.; Howarth, A. J.; Hupp, J. T.; Farha, O. K. *ACS Appl. Mater. Interfaces* **2017**, *9*, 24555.

[6] Cao, M.; Pang, R.; Wang, Q. Y.; Han, Z.; Wang, Z. Y.; Dong, X. Y.; Li, S. F.; Zang, S. Q.; Mak, T. C. W. *J. Am. Chem. Soc.* **2019**, *141*, 14505.

[7] Buru, C. T.; Majewski, M. B.; Howarth, A. J.; Lavroff, R. H.; Kung, C. W.; Peters, A. W.; Goswami, S.; Farha, O. K. *ACS Appl. Mater. Interfaces* **2018**, *10*, 23802.

[8] Wang, Y.; Feng, L.; Pang, J.; Li, J.; Huang, N.; Day, G. S.; Cheng, L.; Drake, H. F.; Wang, Y.; Lollar, C.; Qin, J.; Gu, Z.; Lu, T.; Yuan, S.; Zhou, H.-C. *Adv. Sci.* **2019**, *6*, 1802059.

[9] Feng, L.; Wang, Y.; Yuan, S.; Wang, K.-Y.; Li, J.-L.; Day, G. S.; Qiu, D.; Cheng, L.; Chen, W.-M.; Madrahimov, S. T. ; Zhou, H.-C. *ACS Catal.* **2019**, *9*, 5111-5118.

[10] Amara, Z.; Bellamy, J. F. B.; Horvath, R.; Miller, S. J.; Beeby, A.; Burgard, A.; Rossen, K.; Poliakoff, M.; George, M. W. *Nat. Chem.* **2015**, *7*, 489-495.

[11] Pang, J.; Di, Z.; Qin, J.; Yuan, S.; Lollar, C. T.; Li, J.; Zhang, P.; Wu, M.; Yuan, D.; Hong, M.; Zhou, H.-C. *J. Am. Chem. Soc.* **2020**, *142*, 15020-15026.

[12] Jiang, Z. W.; Zhao, T. T.; Zhen, S. J.; Li, C. M.; Li, Y. F.; Huang, C. Z. *J. Mater. Chem. A* **2021**, *9*, 9301-9306.

[13] Dören, R.; Hartmann, J.; Leibauer, B.; Panthöfer, M.; Mondeshki, M.; Tremel, W. *Dalton Trans.* **2021**, *50*, 14027-14037.

[14] Hendriks, C. M. M.; Lamers, P.; Engel, J.; Bolm, C. *Adv. Synth. Catal.* **2013**, *355*, 3363-3368.

[15] Bulman Page, P. C.; Buckley, B. R.; Elliott, C.; Chan, Y.; Dreyfus, N.; Marken, F. *Synlett* **2016,** *27*, 80-82.

[16] Yuan, J.-P.; Guan, Z.-J.; Lin, H.-Y.; Yan, B.; Liu, K.-K.; Zhou, H.-C.; Fang, Y. *Angew. Chem. Int. Ed.* **2023**, *62*, e202303896.

[17] Xu, X.; Yan, L.; Wang, S.; Wang, P.; Yang, A. X.; Li, X.; Lu, H.; Cao, Z.-Y. *Org. Biomol. Chem.* **2021,** *19*, 8691-8695.

[18] Elsherbini, M.; Allemann, R. K.; Wirth, T. *Chem.-Eur. J.* **2019**, *25*, 12486-12490.
